# In Situ Observation of Polyoxometalate Formation by Vibrational Spectroscopy

**DOI:** 10.1002/chem.202502893

**Published:** 2025-11-17

**Authors:** Jan‐Dominik H. Krueger, Jan‐Christian Raabe, Zainab Yusufzadeh, Andreas Berger, Jakob Albert, Maximilian J. Poller

**Affiliations:** ^1^ Institute of Technical and Macromolecular Chemistry University of Hamburg 20146 Hamburg Germany; ^2^ Institute of Inorganic Chemistry University of Hamburg Hamburg 20146 Germany; ^3^ Mettler‐Toledo Sales & Marketing GmbH 35396 Gießen Germany

**Keywords:** in situ spectroscopy, molybdates, polyoxometalates, synthesis, tungstates

## Abstract

Polyoxometalates (POMs) are multinuclear clusters of transition metals and oxo‐ligands. They have a long history and are still gaining an increased relevance for diverse applications, such as sustainable catalysis. Although the first examples were discovered over a century ago, little is known about the process of their formation. We have now employed modern in situ IR and Raman techniques to gain insights into the processes of the formation of different POM structures in aqueous solution. Thereby, we were able to identify intermediates and optimize reaction conditions. We found that POM formation proceeds very rapidly even at low temperatures, making it unnecessary to reflux the synthesis solutions for multiple hours. With this knowledge, we developed an improved synthetic method for vanadium‐substituted phosphomolybdates, which are of high relevance for sustainable catalysis.

## Graphical abstract

In‐situ IR and Raman spectroscopy were employed to observe the formation of polyoxometalates (POMs) in aqueous solution. Thereby key intermediates were identified, and better understanding of POM formation enabled the development of an improved method for the synthesis of vanadium‐substituted phosphomolybdates, which are important catalysts for sustainable applications. The findings streamline POM synthesis procedures by reducing energy input and shortening reaction times.

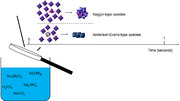

## 1 Introduction

Polyoxometalates (POMs) are anionic polynuclear metal‐oxo complexes formed by elements of group 5 (vanadium, niobium, and tantalum) and group 6 (molybdenum and tungsten) of the periodic table, mostly in their highest oxidation states. Although the field of polyoxometalate chemistry is fairly old—first examples of such compounds have been reported in the 19^th[^ ^1^ ^]^ and early 20^th^ ^[2^ ^]^ century—it is a highly relevant field of modern chemistry. In recent developments, polyoxometalates have found applications, for example as antibacterial agents,^[^ ^3^, ^4^ ^]^ as anti‐amyloid agents,^[5^ ^]^ as chemical memory materials,^[^ ^6^, ^7^ ^]^ and very prominently as catalysts for sustainable chemical processes.^[^ ^8^, ^9^, ^10^, ^11^, ^12^, ^13^ ^]^

**Figure 1:**
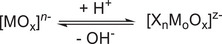
General reaction equation for POM formation in acidic aqueous solution.

POMs are formed in acidic aqueous solution, by polycondensation of the monomeric metalate anions (Figure 1). Their fundamental building block is usually a MO_6_ octahedron. A large variety of POM structures can be constituted by linking such octahedra via their edges or corners. In addition, it is possible to incorporate oxo anions of hetero elements, often main group elements, such as PO_4_^3−^, into the POM structure. Such structure‐types are then called heteropolyanions (HPAs) in contrast to isopolyanions (IPAs), which contain only one framework element.^[^ ^14^, ^15^, ^16^, ^17^, ^18^, ^19^ ^]^ Some of the most well‐known HPA structure‐types are the Keggin‐type [XM_12_O_40_]*^n^* ^−^ (with M = Mo, W; X = P, Si), the Wells‐Dawson‐type [X_2_W_18_O_62_]^6−^ (X = P, As, …; M = W, Mo), and the Anderson‐Evans‐type anions of general formula [XM_6_O_24_]*^n^* ^−^ (M = Mo, W, …; X = p‐/d‐block heteroatom; *n* varies with X oxidation state and protonation; α/β isomers).^[^ ^11^, ^20^ ^]^ The most important IPA structure is the Lindqvist‐type structure with the general stoichiometric formula [M_6_O_19_]*^n^* ^−^ (with M = Mo, W, V, Nb, or Ta).^[14^ ^]^

These POM structures can further be modified by partial substitution of the framework metal with different transition elements, such as vanadium, manganese, cobalt, etc., to introduce the desired catalytic properties for example, redox activity.^[^ ^11^, ^21^, ^22^, ^23^, ^24^, ^25^ ^]^ These so‐called transition‐metal substituted POMs (TMSPOMs), can be obtained either by *ab‐initio* self‐assembly ^[^ ^26^, ^27^, ^28^ ^]^ or via the intermediate formation of a lacunary POM structure.^[^ ^29^, ^30^ ^]^

In the former approach, all precursors are mixed in aqueous solution in the desired stoichiometric ratio, and the POM structure is formed by self‐assembly, induced by carefully adjusting the pH value and temperature.^[^ ^31^, ^32^, ^33^ ^]^ In contrast, the latter approach utilizes a so‐called lacunary POM structure, which is a defect version of a POM structure, in which one or more metal‐oxygen‐octahedra are missing from the framework. The resulting vacancies are highly reactive and can easily be filled with transition‐metal ions to form a TMSPOM.^[^ ^34^, ^35^ ^]^

Although some POMs can be synthesized in fairly short time, e.g. the ammonium salt of phosphomolybdic acid, the formation of which is commonly used as a test for phosphate,^[36^ ^]^ many synthetic procedures demand boiling the solution up to several hours to form the desired POM,^[^ ^37^, ^38^, ^39^ ^]^ an extreme example is the [α‐1,2,3‐PV_3_W_9_O_40_]^6−^, for which a reaction time of 48 hours has been reported.^[40^ ^]^ The increasing relevance of POMs in catalysis, which leads to researchers in the field of chemical engineering and ultimately the chemical industry taking an interest in such compounds, leads to an increasing demand for simple and efficient synthetic procedures.^[^ ^41^, ^42^, ^43^, ^44^, ^45^ ^]^ A prerequisite for this is a greater understanding of POM formation than we currently have.

By applying modern methods that earlier POM chemists like Scheele, Bercelius, Keggin, and Dawson did not have at their disposal, we are now able to shed light on the mechanisms that lead to the assembly of the POM structures. The group of Cronin has applied UV–Vis spectroscopy and ESI‐MS to elucidate the autocatalytic self‐assembly of Keggin‐type POMs and large Mo‐based clusters. They have shown that these clusters, ranging from small Keggin ions to giant Mo154 wheels, can form through autocatalytic networks where smaller clusters template and catalyze the assembly of larger structures.^[^ ^46^, ^47^ ^]^ This has led to some fundamental insights into POM formation. However, the required instrumentation and technology are not open to everyone, and more common approaches should be developed. An option is the nondestructive use of in situ IR and Raman spectroscopy, mostly used for solid‐state analysis of synthesized products. Both technologies offer positive effects, for example, IR is highly sensitive in identifying polar functional groups and metal‐oxygen bonds, whereas Raman measures polarizability and therefore symmetric and nonpolar vibrations, enabling a complementary investigation of POMs. However, IR is limited by water interference and overlapping bands, which are especially in key regions for dissolved metals. Raman is unaffected by this, but fluorescence under laser excitation can limit the measurability by decreasing signal intensity. Both techniques offer fast measurement and cycle times. Combined with up‐to‐date data processing and curation software, they form a highly suitable setup for in situ investigations.

We have now applied such methods to investigate the formation of different POM structures, including V‐substituted phosphomolybdates, which are of high relevance in catalytic applications. For this purpose, we employed a Mettler‐Toledo EasyMax 102 Advanced system for highly controlled reaction conditions together with Mettler‐Toledo's ReactIR and ReactRaman spectrometers and probes. With this research, we aim to optimize the synthetic procedures for POM‐based catalyst materials to facilitate their widespread application. We also want to encourage other researchers to try things outside the regular synthesis rules in order to create greater awareness of optimizations, particularly with regard to sustainability.

## 2 Results and Discussion

### 2.1 Investigation of the Formation of Different Polyoxometalate Structures

To gain insights into the formation of different POM structures, we used in situ IR spectroscopy to observe the formation of Anderson‐Evans, Wells‐Dawson, and Keggin polytungstates under the reaction conditions reported for their respective syntheses. These structures represent the most common types of POMs, of which the unsubstituted tungstates are the most fundamental form.

Our synthesis station ensured an extremely stable reaction environment for maximum isothermy. The precise temperature change of less than 0.5 °C during the reaction, combined with the high dilution of the synthesis mixture and minimal addition quantities, allows the refractive index (RI) to be assumed constant in terms of measurement accuracy (∼1%). The optical path length depends on the wavelength and the ratio of the RI between the reaction mixture and the diamond ATR crystal, so that it can be assumed to be constant for a given wavelength. The extinction coefficient is dependent on wavelength, temperature, and chemical environment. Using the same reasoning, the extinction coefficient can be assumed to be constant within the measurement accuracy. According to Lambert‐Beer's law, changes in peak intensity (fixed wavelength) can be attributed to changes in concentration. Kinetic calculations determine the rate constants and activation energies based on the change in concentration over time. The peak intensities and their changes over time are directly proportional to these concentrations. Thus, the peak intensities themselves can be used to determine the rate constants at a specific temperature.

**Figure 2:**
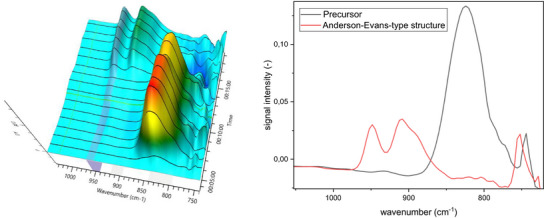
IR spectra for Anderson‐Evans‐type structure formation at 25°C (left). Solvent‐subtraction performed before starting reaction by solvation of precursor (4 minutes, pH 9.66) and formation of Anderson‐Evans‐type structure after addition of telluric acid (15 minutes, pH 8.85, after HCl addition pH 2.86); Extracted IR spectra for the Anderson‐Evans‐type formation at 25°C (right).

For the Anderson‐Evans‐type [TeW_6_O_24_]^6−^, we chose experimental conditions based on our previous work.^[48^ ^]^ Starting with an aqueous solution of sodium tungstate, we initially observed an intense signal at 826 cm^−1^, which we attribute to the *ṽ*(W = O) vibration of the WO_4_^2−^ anion.^[49^ ^]^ Upon addition of telluric acid, this signal vanishes rapidly, and the characteristic vibrational bands of an Anderson‐Evans‐type anion appear instead (Table 1, Figure 2).^[^ ^20^, ^50^ ^]^

**Table 1:** Typical vibrational modes in an Anderson‐Evans‐type structure.

Vibrational mode	Wavenumber [cm^−1^]
W = O_t_	950–830
W‐μ_2_O	825–670
Te‐μ_3_O	650–580

Anderson‐Evans‐type POMs have three different types of oxo ligands: terminal oxo‐ligands attached to the addenda element pointing outwards, μ_2_‐O bridging two addenda metals, and μ_3_‐O bringing two addenda metals and the central heteroelement.^[51^ ^]^ The vibrational bands associated with the W = O_t_ motif are observed in the range of 950–830 cm^−1^, whereby the vibrational bands associated with the bridging μ_2_O and μ_3_O motifs are in the regions of 825–670 cm^−1^ and 650–580 cm^−1^, respectively. Whereby the latter were not within our measurement range. These observed spectra are in line with previous reports.^[^ ^48^, ^50^ ^]^

The Wells‐Dawson‐type synthesis has already presented many scientists with significant challenges, as it is obviously very difficult to find the optimal synthetic parameters.^[^ ^18^, ^52^, ^53^ ^]^ The now generally accepted synthetic procedure of the Wells‐Dawson‐type structure [P_2_W_18_O_62_]^6−^ is the one reported by L. Nadjo et al.,^[53^ ^]^ which includes in one step reflux for 24 hours. This is followed by recrystallization, which extends the overall synthesis time to more than one week. According to L. Nadjo and R. Finke, this synthesis proceeds via the intermediate formation of [W_7_O_24_]^6−^.They also suggest that other intermediates, such as the Preyssler‐type phosphotungstate might be formed.^[50^ ^]^

**Figure 3:**
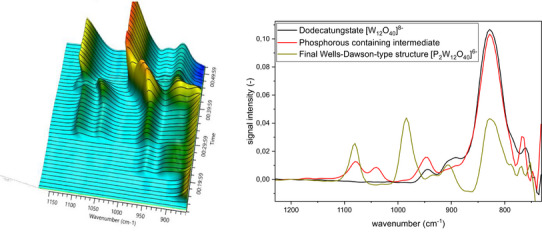
IR spectra for Wells‐Dawson‐type structure formation at 100°C (left). Solvent‐subtraction is performed before starting reaction by solvation of precursor. Extracted IR spectra for the Wells‐Dawson‐type formation at 100°C.

For our experiment, we followed in principle the synthetic procedure described above, whereby an aqueous solution of Na_2_WO_4_ was acidified with HCl before H_3_PO_4_ was added. In the IR spectra (Figure 3), we initially observed the precursor at 826 cm^−1^ (pH 8.75), upon acidification with HCl (to pH 6.63), a different structure was formed, exhibiting weak signals at around 950 cm^−1^ and 900 cm^−1^ as well as a very intense signal at 830 cm^−1^. This matches the chemical equilibrium between dodecatungstate [H_2_W_12_O_40_]^6−^ and heptatungstate [W_7_O_24_]^6−^.^[54^ ^]^

Upon addition of the phosphoric acid (to pH 6.62), we observed that this species transforms to a different intermediate with signals at 1070 cm^−1^ (w), 1040 cm^−1^ (w), 950 cm^−1^ (w), 840 cm^−1^ (s), 760 cm^−1^ (w).^[^ ^20^, ^52^, ^53^ ^]^ Over the course of approx. 10 minutes after the addition of HCl (to pH 1.15), this transforms into the desired final product: Na_6_[P_2_W_18_O_62_], which we identified by its characteristic IR bands at 1080 and 984 cm^−1^.^[53^ ^]^ Overall, the formation of the Wells‐Dawson phosphotungstate was completed in less than 1 hour, indicating that heating for a prolonged time is unnecessary. The endpoint was defined as the point at which no further change in signal intensity was observed, corresponding to a derivative of zero.

**Figure 4:**
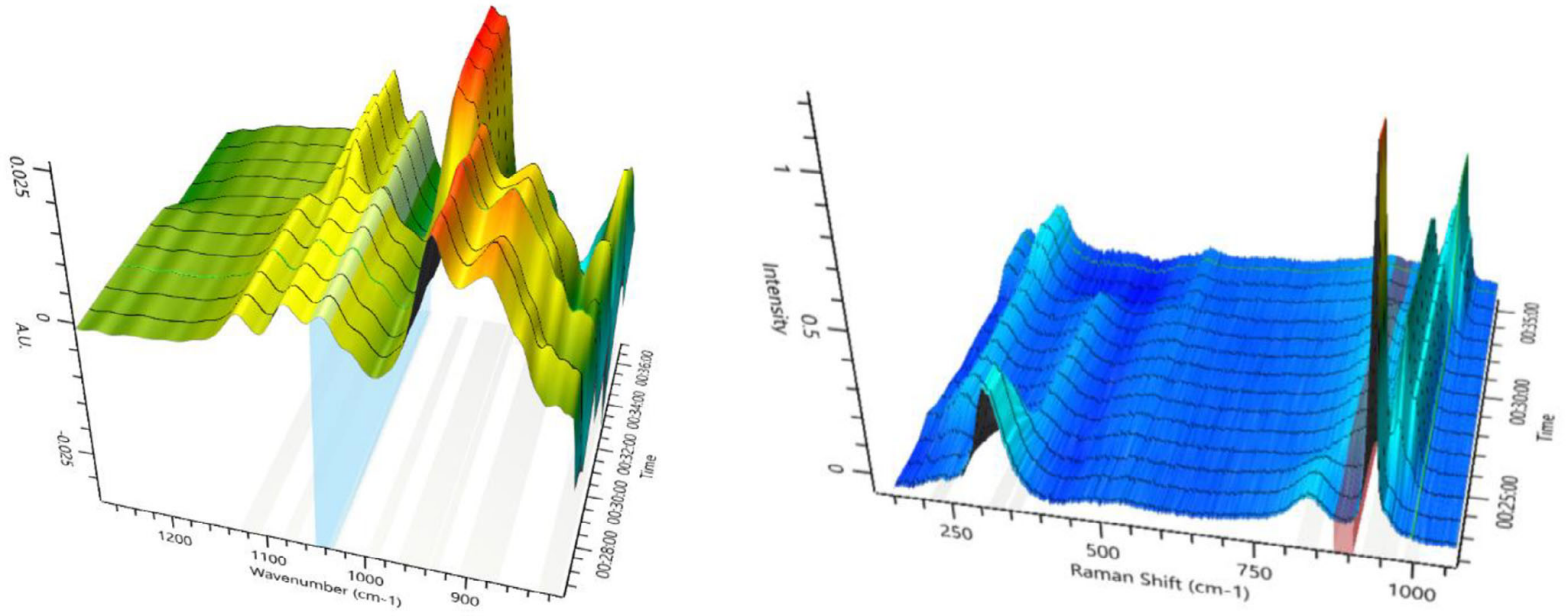
IR (left) and Raman spectra (right) for the Keggin‐type [PW_12_O_40_]^3−^ structure formation at 10 °C. Solvent‐subtraction is performed before starting the reaction by solvation of precursor.

Compared to the Wells‐Dawson synthesis, the synthesis of Keggin‐type [PM_12_O_40_]^3−^ heteropolyacids is a simple and well‐established procedure.^[^ ^25^, ^34^, ^55^, ^56^ ^]^ The usual method involves dissolving the MO_4_^2−^ precursor (M  =  Mo or W) and H_3_PO_4_ in water and adjusting the pH to a highly acidic value.^[16^ ^]^ In a previous study, we observed a species with the composition [P_4_W_14_O_58_]^12−^ at higher pH values around 5, which indicates that this might be an intermediate in the formation of Keggin POMs.^[20^ ^]^ On the other hand, Cronin et al. found that the formation of Keggin POMs proceeds via smaller trinuclear clusters.^[46^ ^]^ For our investigation, we dissolved Na_2_WO_4_ and H_3_PO_4_ in water and then acidified the solution with HCl to a pH of 1. In addition to the in situ IR spectroscopy, we also employed in situ Raman spectroscopy to observe this experiment (Figure 4).

**Figure 5:**
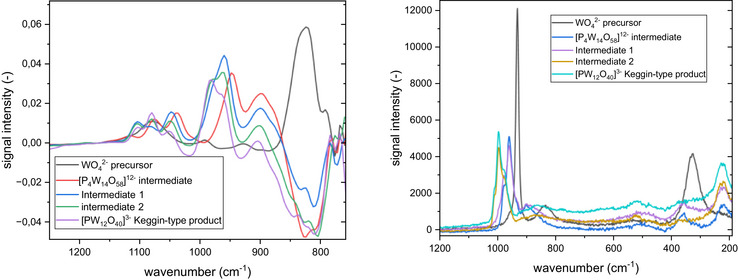
Extracted IR (left) and Raman (right) spectra for the [P_4_W_14_O_58_]^12−^ and Keggin‐type [PW_12_O_40_]^3−^ formation at 10 °C.

Upon dissolution of the precursors at a pH of 8.24 and acidification with HCl (to pH 6.30), we observed the formation of the [P_4_W_14_O_58_]^12−^ intermediate, identified by its characteristic bands at 1082, 1037, 945, 890, 855, and 565 cm^−1^.^[20^ ^]^ Subsequently, while acidifying the solution (to pH 1.08), two other intermediates were observed with signals at 960 cm^−1^ (Intermediate 1) and 963 cm^−1^ and 978 cm^−1^ (Intermediate 2). Negative signals within extracted IR spectra (Figure 5 left) can be led back solely to solvent subtraction (details for data processing for IR and Raman data can be found in the Supporting Information) and low analyte concentrations. Although we are unable to identify these intermediates, we believe it is likely that they are smaller polynuclear complexes as reported by Cronin et al. Finally, after 2 minutes, we observed the spectra matching the characteristic pattern of a Keggin‐type structure.

Comparing both extracted IR and Raman spectra (Figure 5), the signal intensity differences are significant, as Raman shows superior intensities. Even though signal widths are comparably broad (for Raman investigation) and similar to IR spectra, the influence of the required data processing is much smaller, enabling direct observation of precursor conversion or formation of intermediates and products. However, the automated possibility for solvent subtraction within the IR system enables fast analysis options as well. It is precisely this difference in signal intensity that makes it necessary to plan experiments carefully in order to generate evaluable measurements using both methods. While high concentrations are detrimental for Raman experiments, IR measurements would profit from them. In all experiments, the final products were isolated and characterized with IR spectroscopy and elemental analysis (see Supporting Information).

Overall, we have observed the formation of three different POMs, while the Anderson‐Evans POM seems to directly self‐assemble, both the Keggin‐type and Wells‐Dawson‐type POMs form different intermediate species first, before, under the correct pH conditions the target structure is formed.

### 2.2 Kinetic Investigation of POM Synthesis

For a detailed kinetic investigation, we repeated each reaction at two additional temperatures and analyzed the change in concentration of different species over time. Based on the change in the intensity of different peaks, we calculated the relative rate constants.

For the Anderson‐Evans synthesis, we observed the decreasing peak of the WO_4_^2−^ ion and the increasing peak of the [TeW_6_O_24_]^6−^ POM. In the investigation of the Wells‐Dawson synthesis, we observed the peak at 945 cm^−1^ for the phosphorous‐containing intermediate and 984 cm^−1^ for the final Wells‐Dawson‐type product. During the synthesis of the Keggin‐type POM, we evaluated first the formation and then the decline of the [P_4_W_14_O_58_]^12−^ intermediate as well as the formation of the final product. Since the change in intensities appeared mostly linear, we assumed an approximate zero‐order reaction, higher order kinetic models did not yield a better interpretation of the data. The identification of the characteristic bands of the anion [P_4_W_14_O_58_]^12−^ was based on our previous work, in which we isolated and analyzed the anion accordingly. Our measured IR/Raman spectra were compared with the DFT‐simulated spectra to confirm the assignment of the bands.^[57^ ^]^ The analysis of the different intermediates during Wells‐Dawson‐type synthesis proves to be very challenging.^[58^ ^]^ Although ^31^P NMR has provided valuable information for the characterization of phosphorus‐containing POM species (Keggin‐, Wells‐Dawson, Preyssler‐type)^[^ ^59^, ^60^ ^]^ in the past, ^31^P NMR, especially time‐resolved ^31^P NMR spectroscopy, is not helpful due to insufficient time resolution. Even optimized ^31^P NMR experiments require too long measurement times (>3 minutes) for a sufficient analysis of intermediates that appear and disappear within seconds.^[9^ ^]^

From the corresponding Arrhenius plots, we determined the effective activation energies (Table 2).

**Table 2:** Summary of kinetic data for the formation of different polytungstates.

Species	Temperature [°C]	Rate constant k_R_ [ΔI/min]^[a]^	Effective Activation Energy [kJ/mol]
Anderson‐Evans			
WO_4_^2−^ (826 cm^−1^)	0	77 ∙ 10^−3^	8.5
WO_4_^2−^ (826 cm^−1^)	10	96 ∙ 10^−3^	
WO_4_^2−^ (826 cm^−1^)	25	11 ∙ 10^−2^	
[TeW_6_O_24_]^6−^ (948 cm^−1^)	0	16 ∙ 10^−3^	8.7
[TeW_6_O_24_]^6−^ (948 cm^−1^)	10	20 ∙ 10^−3^	
[TeW_6_O_24_]^6−^ (948 cm^−1^)	25	22 ∙ 10^−3^	
Wells‐Dawson			
P‐containing intermediate (945, 946, 953 cm^−1^)	25	8.2 ∙ 10^−3^	−6.1
P‐containing intermediate (945, 946, 953 cm^−1^)	50	8.3 ∙ 10^−3^	
P‐containing intermediate (945, 946, 953 cm^−1^)	80	5.6 ∙ 10^−3^	
P‐containing intermediate (984 cm^−1^)	25	22 ∙ 10^−3^	2.3
P‐containing intermediate (984 cm^−1^)	50	29 ∙ 10^−3^	
P‐containing intermediate (984 cm^−1^)	80	26 ∙ 10^−3^	
[P_4_W_14_O_58_] (intermediate in Keggin Synthesis			
[P_4_W_14_O_58_]^12−^ (819, 810, 827 cm^−1^)	0	13 ∙ 10^−2^	−30
[P_4_W_14_O_58_]^12−^ (819, 810, 827 cm^−1^)	10	49 ∙ 10^−3^	
[P_4_W_14_O_58_]^12−^ (819, 810, 827 cm^−1^)	25	42 ∙ 10^−3^	
[P_4_W_14_O_58_]^12−^ (943, 944, 945 cm^−1^)	0	6.8 ∙ 10^−3^	0.3
[P_4_W_14_O_58_]^12−^ (943, 944, 945 cm^−1^)	10	2.2 ∙ 10^−3^	
[P_4_W_14_O_58_]^12−^ (943, 944, 945 cm^−1^)	25	6.1 ∙ 10^−3^	
Keggin			
[P_4_W_14_O_58_]^12−^ (943, 944, 945 cm^−1^)	0	8.5 ∙ 10^−3^	−7.9
[P_4_W_14_O_58_]^12−^ (943, 944, 945 cm^−1^)	10	1.9 ∙ 10^−3^	
[P_4_W_14_O_58_]^12−^ (943, 944, 945 cm^−1^)	25	5.9 ∙ 10^−3^	
[PW_12_O_40_]^3−^ (979, 980 cm^−1^)	0	5.5 ∙ 10^−3^	−6.2
[PW_12_O_40_]^3−^ (979, 980 cm^−1^)	10	15 ∙ 10^−3^	
[PW_12_O_40_]^3−^ (979, 980 cm^−1^)	25	4.6 ∙ 10^−3^	

^[a]^ ΔI = difference in signal intensity.

While it appears that the rate constants in some cases decrease with increasing temperature, we suspect that they are not strongly temperature dependent, and the apparent decrease is within the error of our measurement. Similarly, all of the effective activation energies are very small values (mostly <10 kJ/mol), some in the negative range. From this we conclude, that the actual activation energies are smaller than the error of our measurement. This finding is in line with the common conception, that POMs spontaneously self‐assemble in aqueous solution under the right conditions. It also shows that high temperatures (i.e., reflux in water) are not necessary for the POM self‐assembly reaction, although elevated temperatures might be beneficial for dissolution of the precursor materials.

### 2.3 Comparison of Keggin‐Type Molybdate and Tungstate Formation

**Figure 6:**
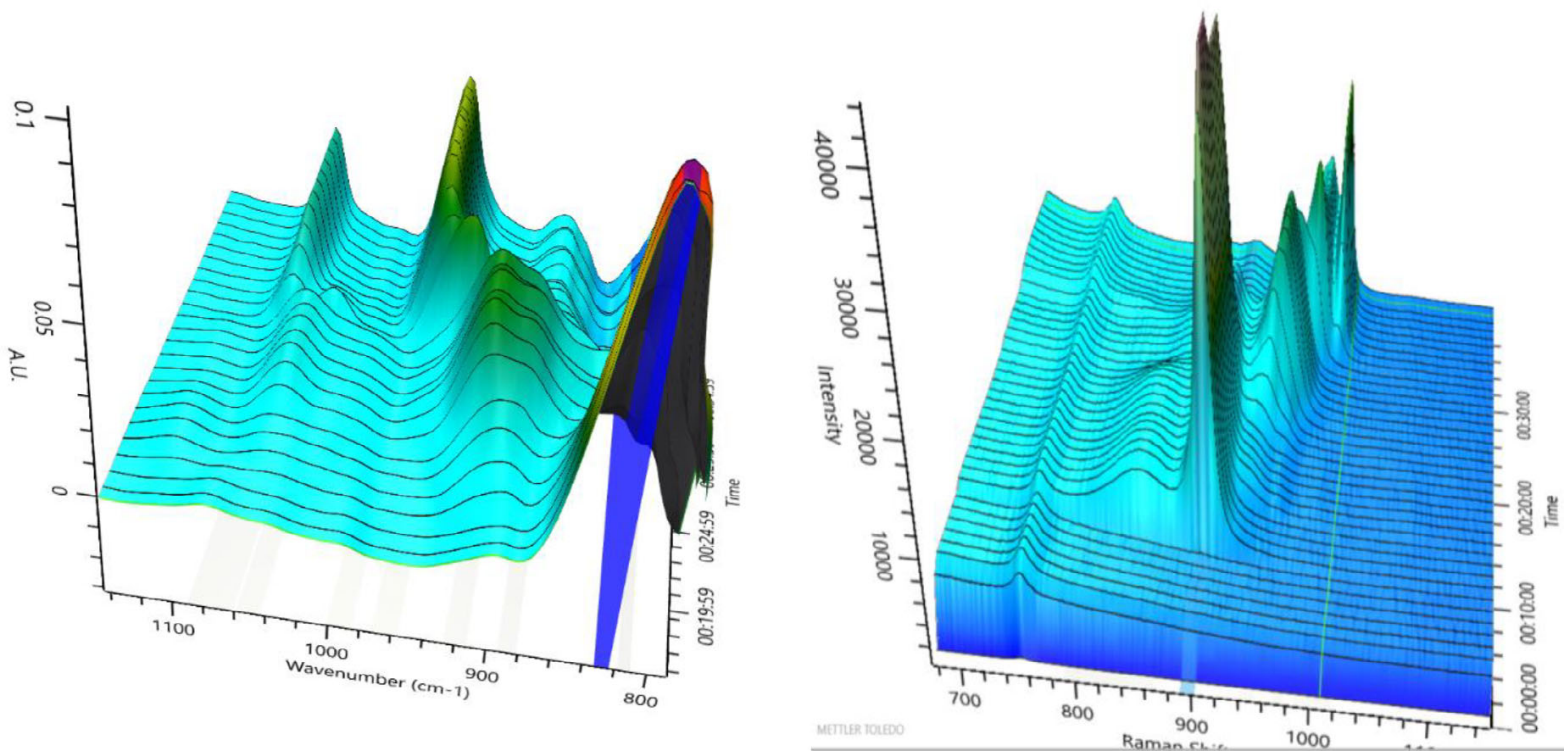
IR spectra (left) and Raman spectra (right) for [PMo_12_O_40_]^3−^ Keggin‐type structure formation at 25°C. Solvent‐subtraction is performed before starting reaction by solvation of precursor.

**Figure 7:**
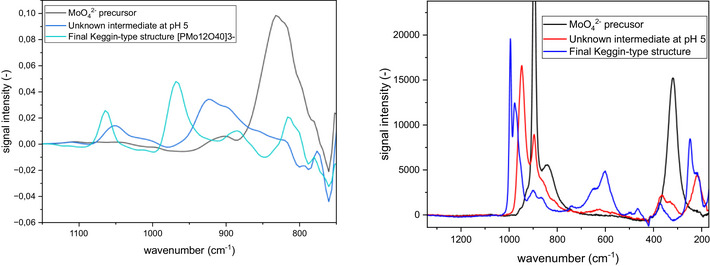
Extracted IR (left) and Raman (right) spectra for the Keggin‐type [PMo_12_O_40_]^3−^ formation at 25 °C.

After investigating the different tungstate structures, the next step in our study was to determine the transferability of our findings to molybdate POMs. To this end, we studied the synthesis of a Keggin‐type phosphomolybdate (Figure 4) in the same way, using in situ IR and Raman spectroscopy, and compared the results with the above‐described experiment on the Keggin‐type phosphotungstate (Figures 6 and 7).

Similar to the tungstate experiment, upon mixing the molybdate precursor with phosphoric acid (approx. pH 5), we observed an intermediate with multiple broad signals in the IR at around 940‐800 and 1050‐1000 cm^−1^ as well as Raman signals at 948, 897, 624, 366, and 220 cm^−1^. Although it is impossible to distinguish the broad signals sufficiently, the pattern seems similar to the signals of the above‐mentioned [P_4_W_14_O_58_]^12−^ species. Combined with the fact that it has been formed under similar conditions, we therefore propose the formation of [P_4_Mo_14_O_58_]^12−^ as a possible intermediate. The formation of polymolybdate species corresponding to the above‐mentioned intermediate 1 and intermediate 2 has not been observed. A possible reason for this might be the fact that polymolybdate species are less stable than the corresponding tungstates and undergo more rapid transformation, making it more difficult to observe intermediates. Upon acidification with hydrochloric acid, the formation of the Keggin phosphomolybdate, identified by its characteristic IR and Raman bands, was observed. The final product was isolated and characterized by IR spectroscopy (SI, Figure S15).

For the kinetic evaluation, we evaluated the precursor peak (MoO_4_^2−^) at 831 (30 °C), 826 (10 °C), and 816 cm^−1^ (0 °C), the intermediate with signals at 921 and 925 cm^−1^, and for the final Keggin‐type product the peaks at 966 and 964 cm^−1^ (Table 3).

**Table 3:** Summary of kinetic data for Keggin molybdate formation.

Species	Temperature	Rate constant k_R_ [ΔI/min]^[a]^	Effective Activation Energy
**Intermediate formation**			
MoO_4_^2−^ (831, 826, 816 cm^−1^)	0 °C	41 ∙ 10^−3^	−20 kJ/mol
MoO_4_^2−^ (831, 826, 816 cm^−1^)	10 °C	10 ∙ 10^−2^	
MoO_4_^2−^ (831, 826, 816 cm^−1^)	30 °C	21 ∙ 10^−3^	
[P_4_Mo_14_O_58_]^12−^ (921, 925 cm^−1^)	0 °C	10 ∙ 10^−3^	−4 kJ/mol
[P_4_Mo_14_O_58_]^12−^ (921, 925 cm^−1^)	10 °C	50 ∙ 10^−3^	
[P_4_Mo_14_O_58_]^12−^ (921, 925 cm^−1^)	30 °C	11 ∙ 10^−3^	
**Keggin formation**			
[P_4_Mo_14_O_58_]^12−^ (921, 925 cm^−1^)	0 °C	7.4 ∙ 10^−3^	7 kJ/mol
[P_4_Mo_14_O_58_]^12−^ (921, 925 cm^−1^)	10 °C	12 ∙ 10^−3^	
[P_4_Mo_14_O_58_]^12−^ (921, 925 cm^−1^)	30 °C	11 ∙ 10^−3^	
[PMo_12_O_40_]^3−^ (966, 964 cm^−1^)	0 °C	10 ∙ 10^−3^	22 kJ/mol
[PMo_12_O_40_]^3−^ (966, 964 cm^−1^)	10 °C	21 ∙ 10^−3^	
[PMo_12_O_40_]^3−^ (966, 964 cm^−1^)	30 °C	29 ∙ 10^−3^	

^[a]^ ΔI = difference in signal intensity.

Similar to the tungstates described above, the measurement error in the kinetic investigation of the molybdate seems to be significantly larger than the actual values of the activation energies. Again, this is in line with the idea, that POMs spontaneously self‐assemble without an activation barrier. It also shows that tungstates and molybdates are comparable in this aspect. The fact that for some tungstate intermediates the corresponding molybdates are not observed supports the idea that molybdates are more labile and thus transform more rapidly.

### 2.4 Transition‐Metal‐Substituted Keggin‐Type Phosphomolybdates

After studying the monometallic POMs, the next step of our investigation was the synthesis of bi‐metallic POMs. Especially, vanadium‐substituted phosphomolybdates are of increasing interest to researchers in the fields of catalysis and chemical engineering.^[^ ^59^, ^60^, ^61^ ^]^ Since this necessitates efficient synthesis of larger quantities, we decided to exemplarily study two synthetic pathways for [PV_2_Mo_10_O_40_]^5−^. The self‐assembly pathway is based on a procedure by V. Odyakov and E. Zhizhina, in which a vanadium precursor is prepared from V_2_O_5_, H_2_O_2+_, and H_3_PO_4_, which is then added to an aqueous solution of H_3_PO_4_ and MoO_3._ ^[62^ ^]^ In contrast, for the other approach, a trilacunary‐Keggin phosphomolybdate is created first, before two equivalents of V precursor and one equivalent of Mo precursor are added to complete the full Keggin structure.^[34^ ^]^ (For experimental details, see Section 4.6)

**Figure 8:**
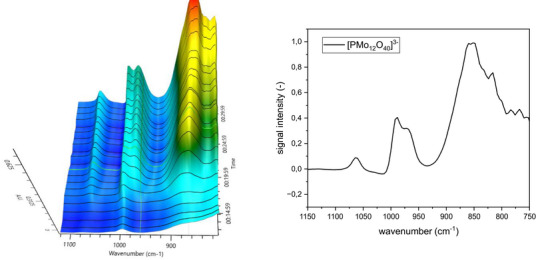
Formation of [PMo_12_O_40_]^3−^, IR spectra (left) and extracted spectrum at the end of reaction (right).

For our study of the self‐assembly procedure, we first produced the aqueous solution of H_3_PO_4_ and MoO_3_. Upon complete dissolution of the MoO_3_ we observed the formation of the characteristic IR bands of [PMo_12_O_40_]^3−^ (1064, 968, 884, and 816 cm^−1^, Figure 8). Due to the stoichiometric ratio of 1 P:10 Mo, we assume free phosphoric acid must also be present in this mixture.^[31^ ^]^

**Figure 9:**
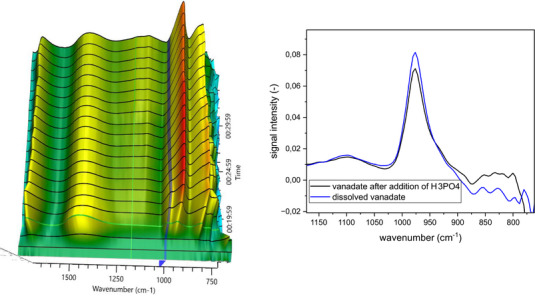
Solvation of V_2_O_5_ using H_2_O_2_, IR spectra (left) and extracted spectra (right) after solvation and after addition of H_3_PO_4_.

In parallel, V_2_O_5_ was dissolved in aqueous H_2_O_2_ solution (Figure 9) yielding an intermediate with a peak at 978 cm^−1^, which, according to Zhizhina's report, we attribute to the decavanadate species H_6_[V_10_O_28_]. According to Zhizhina, after addition of H_3_PO_4_ the formation of H_9_[PV_14_O_42_] should be visible. However, after addition of phosphoric acid, we weren't able to detect any changes. Therefore, we were not able to observe the [PV_14_O_42_]^9−^ species reported in the literature. The reason for this might be differences in procedure and reaction conditions or simply failure to detect it although it might have been formed.^[33^ ^]^

**Figure 10:**
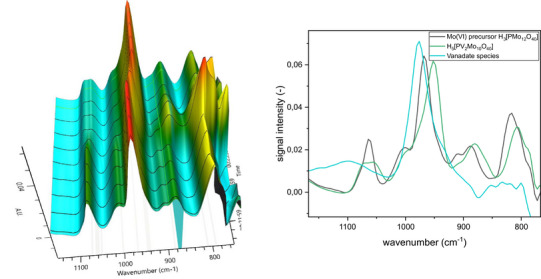
IR investigation of the H_5_[PV_2_Mo_10_O_40_] preparation (self‐assembly approach): formation of the final H_5_[PV_2_Mo_10_O_40_] Keggin‐type species (left) and extracted spectra (right).

Finally, both precursor solutions were combined in the correct stoichiometric ratio (P:V:Mo 1:2:10), by adding the V solution to the Mo solution. Thereby, we observed a slight shift in the signals of the [PMo_12_O_40_]^3−^ anion, indicating the formation of the final product [PV_2_Mo_10_O_40_]^5−^ (Figure 10), which was completed after 5 minutes.

In general, the IR bands of [PV_2_Mo_10_O_40_]^5−^ are shifted to lower wavenumbers compared to the unsubstituted Keggin‐type species [PMo_12_O_40_]^3−^. The reason for this is the lower mass of V compared with Mo, therefore the V‐O vibrations are excited at different energies than Mo‐O vibrational modes. This also leads to the splitting of the P‐O vibrational band at approx. 1070 cm^−1^.

For the lacunary approach, a solution of Na_2_MoO_4_ and H_3_PO_4_ was acidified to a pH value of approx. 1, whereby the IR signal for MoO_4_^2−^ at 825 cm^−1^ decreased, while the signals for the trilacunary [PMo_9_O_34_]^9−^ (1064, 1012, 948, 924 cm^−1^) increased. Subsequently, two equivalents of NaVO_3_ and one equivalent of Na_2_MoO_4_ were added in the form of aqueous solutions. This led to a decrease in the lacunary species and a corresponding increase in the product [PV_2_Mo_10_O_40_]^5−^ (964 cm^−1^).^[^ ^25^, ^34^ ^]^

**Figure 11:**
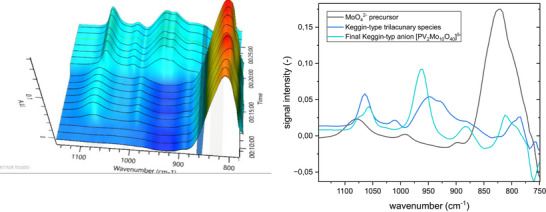
IR investigation of the Na_5_[PV_2_Mo_10_O_40_] preparation (lacunary approach): formation of the trilacunary species (left) and extracted spectra (right).

For the lacunary approach, the 3D plot and the extracted IR spectra over time are found in Figure 11.

For a kinetic analysis of the self‐assembly reaction (Table 4), we observed the increase of peak 968 cm^−1^ (H_3_[PMo_12_O_40_] formation, ṽ(Mo = O_t_)) and after addition of the V(V) species, we investigated the increase of peak 952 cm^−1^(Mo/V = O_t_).

**Table 4:** Summarized kinetic data for substitution experiments.

Species	Temperature [°C]	Rate constant k_R_ [ΔI/min]^[a]^	Effective Activation Energy [kJ/mol]
**Self‐Assembly of [PV_2_Mo_10_O_40_]^5−^**			
ṽ(Mo/V = O_t_) (948, 973 cm^−1^)	25	9.8 ∙ 10^−3^	−4.3
ṽ(Mo/V = O_t_) (948, 973 cm^−1^)	75	20 ∙ 10^−3^	
ṽ(Mo/V = O_t_) (948, 973 cm^−1^)	100	4.9 ∙ 10^−3^	
ṽ(Mo = O_t_) (995 cm^−1^)	25	2.9 ∙ 10^−3^	24
ṽ(Mo = O_t_) (995 cm^−1^)	75	22 ∙ 10^−3^	
ṽ(Mo = O_t_) (995 cm^−1^)	100	17 ∙ 10^−3^	
**Synthesis of [PMo_9_O_34_]^9−^**			
ṽ(Mo‐O‐Mo) (818, 820, 825 cm^−1^)	−5	78 ∙ 10^−3^	16
ṽ(Mo‐O‐Mo) (818, 820, 825 cm^−1^)	10	13 ∙ 10^−2^	
ṽ(Mo‐O‐Mo) (818, 820, 825 cm^−1^)	25	15 ∙ 10^−2^	
ṽ(P‐O) (1064 cm^−1^)	−5	13 ∙ 10^−3^	3.3
ṽ(P‐O) (1064 cm^−1^)	10	20 ∙ 10^−3^	
ṽ(P‐O) (1064 cm^−1^)	25	14 ∙ 10^−3^	
**Synthesis of [PV_2_Mo_10_O_40_]^5−^ via lacunary approach**			
ṽ(P‐O) (1064 cm^−1^)	−5 °C	11 ∙ 10^−3^	−12
ṽ(P‐O) (1064 cm^−1^)	10 °C	11 ∙ 10^−3^	
ṽ(P‐O) (1064 cm^−1^)	25 °C	6.4 ∙ 10^−3^	
ṽ(Mo/V = O_t_) (951, 961 cm^−1^)	−5 °C	25 ∙ 10^−3^	5.7
ṽ(Mo/V = O_t_) (951, 961 cm^−1^)	10 °C	29 ∙ 10^−3^	
ṽ(Mo/V = O_t_) (951, 961 cm^−1^)	25 °C	32 ∙ 10^−3^	

^a^ ΔI = difference in signal intensity.

As in the investigations of the formation of monometallic POMs above, the relative rate constants and resulting activation energies that we determined for the formation of [PV_2_Mo_10_O_40_]^5−^ appear to be very small and are exceeded by their error of measurement (Table 4). This is evident by the large spread of the results. As above, this indicates that there is no activation barrier for the POM formation and synthesis can be performed very quickly, even at low temperatures.

### 2.5 Optimization of Synthesis Procedure

Based on our findings, which indicate that those POM structures with the classic elemental compositions we investigated within this study form rapidly once the precursors are dissolved and the correct pH value is set, we propose the following optimization for the self‐assembly synthesis of vanadium‐substituted phosphomolybdates:

- Stabilization of the V precursor by the addition of H_3_PO_4_ appears unnecessary.
- Instead, the correct stoichiometric amount of H_3_PO_4_ is added to the MoO_3_ solution, this aids in the dissolution of MoO_3_ by the formation of [PMo_12_O_40_]^3−^
- The cold V precursor solution can be added rapidly to the hot [PMo_12_O_40_]^3−^ solution since high temperatures are not necessary, it is not important to maintain reflux throughout the synthesis

Based on these conclusions, we successfully performed the optimized synthesis of H_5_[PV_2_Mo_10_O_40_] (details see Section 4.8), with significantly less effort and time. We achieved yield and purity comparable to previous literature reports (Figures S40–S42 and Tables S18–S19).^[^ ^25^, ^62^ ^]^ The only limitation we encountered was excessive foaming when the peroxide‐rich V precursor solution was added to the hot [PMo_12_O_40_]^3−^ solution. To mitigate this, we advise a slow and cautious addition.

## 3 Conclusion

In summary, we were able to observe the formation of different POM structures by in situ vibrational spectroscopy and thereby gained unprecedented insights, including information about intermediates. Although the exact values we determined are subject to a large error, we found that activation energies for POM formation are very small or nearly nonexistent. Further studies with improved experimental design are necessary to determine more reliable values. Furthermore, we gained insights into the mechanisms of transition‐metal substitution in Keggin‐type phosphomolybdates, a class of compounds, which as high relevance for catalytic applications. Based on our observations, we were able to optimize the synthesis, saving significant amounts of time and effort. This will greatly enhance the applicability of these POMs on a larger scale.

## 4 Experimental Methods

### General experimental set‐up

**Figure 12:**
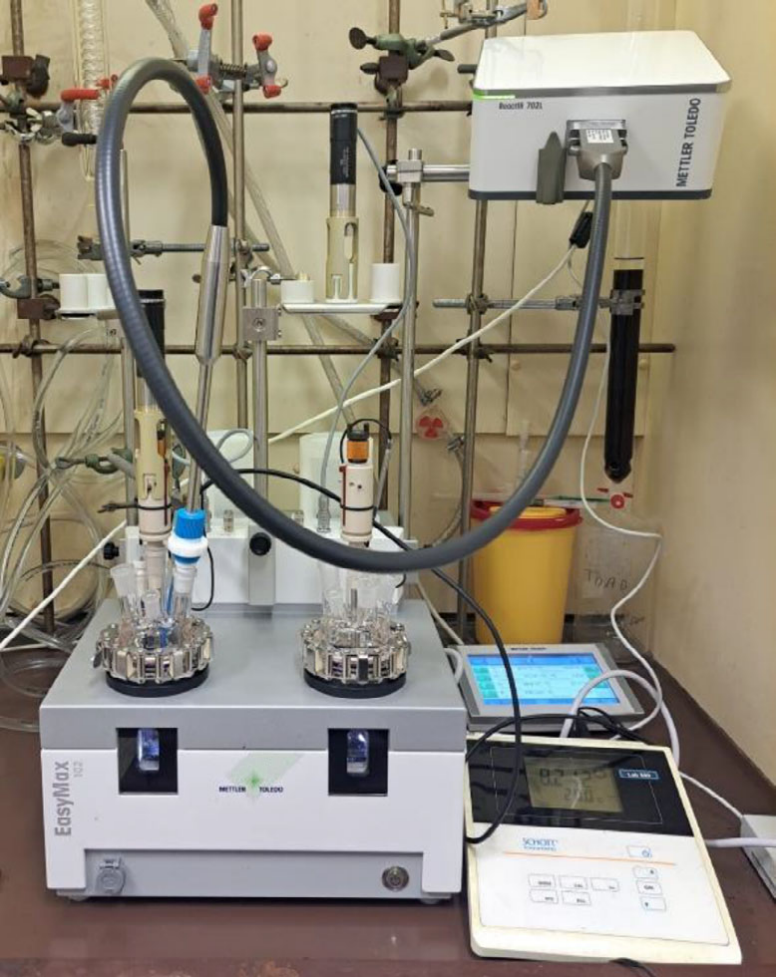
Setup for in situ investigations with ReactIR 702L probe within EasyMax 102.

Syntheses were carried out in EasyMax 102 Advanced thermostat, which stabilizes temperature through electric heating and cooling (Figure 12). Integration of pitched blade propeller stirring ensures quick homogenization, especially following reagent additions. This is an advantage for maintaining isothermal conditions. The modular setup can be assembled to include automated dosing and pH control.

In situ mid infrared spectra were continuously collected with ReactIR 702L with a flexible fiber probe DST 9.5 mm × 305 mm × 1.5m DiComp. This setup follows the attenuated total reflectance (ATR) method using a broad band SiC light source and a diamond ATR crystal. Spectra were collected at 8 cm^−1^ spectral resolution and the AutoSelect function in software iC IR 7.1 coaveraged continuously collected scans in a one‐minute time interval. Peak intensities *A* are directly proportional to the concentration *c* of observed functional group, as described in Lambert‐Beer's law (see Equation 1). Molar extinction coefficient $\varepsilon_{M}$ is dependent on temperature *T* and wavenumber $\tilde{\nu }$ and can be assumed constant for a given wavenumber in isothermal conditions. Optical pathlength *d* in ATR‐type measurements depends on the refractive index of sample and ATR crystal, which can be assumed constant for the described experiments due to the high degree of dilution in the aqueous solution.

(1) $$A=\varepsilon_{M}\;\cdot \;d\;\cdot \;c$$

A ReactRaman 802L instrument equipped with an immersion probe was used for in situ Raman spectroscopy. Spectra were continuously collected at 400 mW laser power at the probe tip with a 785 nm excitation wavelength and 3.1 seconds exposure time. To monitor the syntheses, 17 scans are coaveraged to a spectrum every minute using AutoSelect function in its software iC Raman 8.0.

Peak intensity in Raman spectra can similarly be correlated to the concentration of the observed functional group. Aside from instrument‐specific parameters affecting the sensitivity, signal intensity depends on laser output power and exposure time for the individual spectra. Both spectroscopic tools were used within their specifications, enabling a standard deviation of signals < 1 %.

Our approach to data evaluation, processing, and curation can be found in the first chapter of Supporting Information. A detailed procedure is shown for the synthesis of an Anderson‐Evans structure. The procedure was performed for all other experiments.

All synthetic procedures for the different POM structures were tested before investigating kinetic behavior. As POM formation is highly dependent on pH values, we performed each synthesis step, while observing spectra with the ReactIR by slowly adjusting the pH value and noting the required amount. Within the kinetic experiment, the defined amount needed was added instantaneously. The specific amounts for each synthesis are provided in the following sections.

Synthesized structures were purified with a nanofiltration membrane setup described in a previous publication^[63^ ^]^

### Equations for the kinetic evaluation

For our evaluation, we assumed a zero‐order reaction, with a constant reaction rate k_R_. The reaction rate is therefore independent of the reactant/product concentration, see Equation 2:

(2) $$\frac{d[\mathrm{Product}]}{dt}=-\frac{d[\mathrm{Educt}]}{dt}=k_{R}$$

The data were used for creating the Arrhenius plot, see Equation 3:

(3a) $$k_{R}=A\;\cdot \;e^{-\;\frac{E_{A}}{RT}}\;$$

(3b) $$\ln\left(k_{R}\right)=\ln\left(A\right)\;-\;\frac{E_{A}}{RT}$$

By logarithmically plotting the Arrhenius equation (3a), a plot of the logarithmic reaction rate (constant) ln(*k*_R_) against the reciprocal temperature 1/*T* is obtained. The *y*‐axis intercept corresponds to the logarithmic frequency factor ln(*A*) of the reaction, *E*_A_ to the activation energy, R the ideal gas constant (8.314 J/K mol), and *T* to temperature (3b).

The slope of the linear fits is called the Arrhenius parameter of the reaction, see Equation 4:

(4) $$\mathrm{Arrhenius}\;\mathrm{parameter}=-\frac{E_{A}}{R}$$

### Synthesis of Anderson‐Evans

To Na_2_WO_4_ (5.001 g, 15.16 mmol, 5.98 equivalents) dissolved in deionized water (100 mL), Te(OH)_6_ (0.5821 g, 2.53 mmol, 1 equivalent) was added at 20 °C. Then, to adjust the pH to 5, a 37 % HCl solution in water (1.8 mL) was added.

Subsequently, the synthesis of Anderson‐Evans tungstate was done at 30 °C, 25 °C, 10 °C, and −5 °C. The formation of desired products aforementioned temperatures was confirmed via IR‐ and ICP‐OES‐Analysis (see Supporting Information Figure S26 and Table S14).

### Synthesis of Wells‐Dawson

For the synthesis of Wells‐Dawson‐type tungstate, Na_2_WO_4_ (5.001 g, 15.16 mmol, 8.92 equivalents) was dissolved in deionized water (100 mL) at 100 °C. Then, a 2 M solution of H_3_PO_4_ in water (0.85 mL, 1.70 mmol, 1 equivalent) was added. To adjust the pH to 1, a 4 M HCl solution in water (20.8 mL) was added at room temperature.

Subsequently, the synthesis of Wells Dawson phosphate tungstate was done at 30°C, 50°C, and 80°C. The formation of Wells‐Dawson‐type structures at aforementioned temperature was confirmed via IR‐ and ICP‐OES‐Analysis (see Supporting Information Figures S27 and Table S15).

### Synthesis of Keggin molybdate and tungstate

For the synthesis of the Keggin‐type phosphomolybdate, Na_2_MoO_4_ (3.668 g, 15.15 mmol, 12 eqv.) was dissolved in deionised water. At 0°C/10°C/25°C, 85% H_3_PO_4_ (0.148 g, 1.29 mmol, 1 eqv.) was added, forming a clear yellow solution. Afterwards, adjusting pH to 1, 2.113 g of a 37% HCl solution was added. The reaction solution turned orange.

For synthesis of Keggin tungstate, Na_2_WO_4_ x H_2_O (5.002 g, 15.2 mmol, 12 eqv.) was dissolved in deionised water. At 0°C/10°C/25°C, 0.149 g (1.28 mmol, 1 eqv.) of a 85 % H_3_PO_4_ solution was added. Afterwards, to adjust pH to 1, 2.199 g of a 37 % HCl solution was added.

The Purification is carried out using a membrane system, which is described in previous publications.

The Formation of desired products could be observed and characterized via IR spectroscopy and ICP‐OES‐Analysis before and after the purification (see Supporting Information Tables S16 and S17).

### Synthesis of Self‐Assembly vanadium substitution

One of the pathways to synthesize transition metal element‐substituted Keggin‐type POMs is self‐Assembly.

For the synthesis of HPA‐2, the self‐assembly method was used. For this purpose, two parent solutions were prepared.

Solution 1: MoO_3_ (5.001 g, 15.16 mmol, 4.36 equivalents) was dissolved in deionized water (42 mL). Then, an 85 % solution of H_3_PO_4_ in water (0.4013 g, 3.48 mmol, 1 equivalent) was added, and the suspension was refluxed at 110°C until a clear solution was obtained.

Solution 2: V_2_O_5_ (1.2616 g, 6.94 mmol, 2 equivalents) was added to deionized water (48 mL) and cooled to 4 °C. After reaching a stable temperature, an aqueous solution of H_2_O_2_ in water (7 mL) was added to the solution, and the residue was washed with deionized water (5 mL). This solution was stirred for 1 h and then warmed to 20°C. Finally, an aqueous solution of 85 % H_3_PO_4_ (0.074 g, 0.64 mmol, 0.18 equivalent) was added.

In a 100 mL bottle, deionized water (55 mL) was added. 1/3 of solution 1 was added to this flask and refluxed at 100°C. 1/3 of solution 2 was added and stirred for a few minutes. The reaction solution turned orange. The reaction was done at 50°C and 25°C. The formation of desired products aforementioned temperatures was confirmed via IR‐ und ICP‐OES‐Analysis (see Supporting Information Table S18).

### Synthesis of Lacunary Vanadium‐substitution

The formation of a Lacunary‐type structure is used for the synthesis of element‐substituted Keggin‐type POMs. To synthesize the Keggin‐Lacunary‐type structure in situ, parent solutions were prepared. NaVO_3_ (1.679 g, 13.77 mmol, 2 equivalents) was dissolved in deionized water (30 mL) (solution 1). Na_2_MoO_4_ (1.667 g, 6.89 mmol, 1 equivalents) was dissolved in water (30 mL) (solution 2).

First, Na_2_MoO_4_ (5.0378 g, 20.82 mmol, 7.37 equivalents) was dissolved in deionized water (90 mL). Then, an aqueous solution of H_3_PO_4_ (0.3258 g, 2.82 mmol, 1 equivalents) was added. To determine the amount of HCl to adjust the pH to 1 at 25°C, aqueous HCl (5.2063 mL) was added. For other investigations at 25 °C, 10 °C and ‐5 °C, the same amount of HCl (5.2063 mL) was added to adjust the pH to 1.

Solution 1 (10 mL) was added to the in situ synthesized Keggin‐Lacunary‐type solution at the desired temperature. The solution turned orange. Afterwards, solution 2 (10 mL) was added. The formation of desired products at the aforementioned temperatures was confirmed via IR‐ and ICP‐OES analysis (see Supporting Information Table S19).

### Optimized synthesis method for vanadium‐substituted phosphomolybdates

In this work, we present a general optimized method for synthesizing vanadium‐substituted phosphomolybdates, based on new knowledge gained during our experiments. In contrast to previous synthesis methods, no phosphoric acid is needed to stabilize the dissolved vanadate species.

Solution 1: To a cooled suspension of V_2_O_5_ (6.367 g, 0.035 mol, 1 eqv.) in 200 mL of water (0‐5°C), H_2_O_2_ (37 mL of 30 % H_2_O_2_ aqueous solution) was added dropwise to obtain a clear solution.

Solution 2: In another flask, a stoichiometric amount of MoO_3_ (50.340 g, 0.350 mol,10 eqv.) and H_3_PO_4_ (85 % aqueous solution, 4.046 g, 0.035 mol,1 eqv.) were dissolved in deionized water and refluxed.

Solution 1 was slowly added to solution 2. A significant gas evolution was observed. The mixture was stirred until a homogeneous solution was obtained (about 5 min). Then, the mixture was filtered, and the solvent was removed under vacuum. The formation of desired products (59.5 g, 0.034 mol, 97.8 % yield) was confirmed via IR‐ (Figure S40) and ICP‐OES‐analysis (P/V/Mo ratio of 1.0/2.0/10.0) and as well as ^31^P‐ and ^51^V‐NMR spectroscopy (Figure S41 and S42).

## Author Contributions

The manuscript was written through the contributions of all authors. Jan‐Dominik H. Krueger: Conceptualization, data curation, formal analysis, investigation, methodology, visualization, writing. Jan‐Christian Raabe: Conceptualization, data curation, formal analysis, investigation, methodology, visualization, writing. Zainab Yusufzadeh: Investigation, writing (review and editing). Andreas Berger: Methodology, Resources, writing (review and editing). Jakob Albert: Resources, writing (review and editing). Maximilian J. Poller: Conceptualization, project administration, supervision, methodology, writing.

## Conflicts of Interest

A. Berger works for Mettler‐Toledo Sales & Marketing Services GmbH. The other authors declare no conflict of interest.

## Supporting information



Supporting Information

## Data Availability

Data is provided in the electronic supplementary information file. Additional data will be made available on request.
