# Supplementary material for: In Situ Observation of Polyoxometalate Formation by Vibrational Spectroscopy
Source: Chemistry. 2025 Nov 17;31(72):e02893. doi: 10.1002/chem.202502893 (PMC12731537; doi:10.1002/chem.202502893)
Supplement: Supplementary file 1 — Supporting Information [file CHEM-31-e02893-s001.pdf]

In-situ observation of Polyoxometalate formation by vibrational spectroscopy – Supporting Information

Jan-Dominik H. Krueger[1⊥], Jan-Christian Raabe[1⊥], Zainab Yusufzadeh[2], Andreas Berger[3], Jakob Albert[1], Maximilian J. Poller[1*]

[1] Institute of Technical and Macromolecular Chemistry, University of Hamburg, Bundesstraße 45, 20146 Hamburg, Germany

[2] Institute of Inorganic Chemistry, University of Hamburg, Martin-Luther-King-Platz 6, 20146 Hamburg, Germany

[3] Mettler-Toledo Sales & Marketing GmbH, Ockerweg 3A, 35396 Gießen, Germany

⊥Shared first-authorship

*Corresponding author: maximilian.poller@uni-hamburg.de

Table of Contents

Explanation of Data Processing 2

Kinetic Data 10

- Anderson-Evans Structure 10
- Wells-Dawson Structure 11
- Keggin Structure 13
- Synthesis of $[PV_2Mo_{10}O_{40}]^{5-}$ via lacunary route 19
- Synthesis of $[PV_2Mo_{10}O_{40}]^{5-}$ via self-assembly 21

Spectroscopic Investigations (solid state) 23

Spectroscopic Investigations (liquid state) 27

Elemental Analysis 31

Spectroscopic data of $H_5PV_2Mo_{10}O_{40}$ (HPA-2) synthesized by optimized method 33

Chemicals 34

Procedures, times and pH values for Anderson-Evans synthesis 35

Procedures, times and pH values for Wells-Dawson synthesis 37

Procedures, times and pH values for Keggin tungstate synthesis 39

Procedures, times and pH values for Keggin molybdate synthesis 42

Procedures, times and pH values for synthesis of $[PV_2Mo_{10}O_{40}]^{5-}$ via lacunary route 44

Explanation of Data Processing

For data collection, reaction control and data processing software supplied by Mettler Toledo was used. For data processing of IR spectra iC IR 7.2. and for raman iC Raman 8.0. were used. The reaction control of the EasyMax 102 Advanced thermostat was performed using iC Control 6.2. For easier understanding, we'll show an exemplarily investigation of data sets for the synthesis of a Keggin tungstate structure at 10 °C. Further information on used instrumentation is shown in Figure S 1.

Info

Experiment

Status	Date	Time	Elapsed	Samples
Started	1/26/2024	11:23:51 AM	00:00:00	
Completed	1/26/2024	12:05:50 PM	00:41:58	41

Instrument

Name	ReactRaman 802L-4146
	Instrument: ReactRaman802L, Serial Number: C219014146, Sampling from 3400 to 150 cm-1; Probe Type: ReactRaman probe, Probe SN: 000-000-000, Probe Tube SN: Immersion, Cosmic Ray Removal: Enabled; Laser Power: 400 mW, Scans: 17, Exposure Time: 3.1 sec, Gain: High, Dark Correction: Auto, White Light Correction: Disabled, Apply Instrument Normalization: Disabled

Info

Experiment

Status	Date	Time	Elapsed	Samples
Started	1/26/2024	11:23:51 AM	00:00:00	
Completed	1/26/2024	12:05:52 PM	00:42:00	41

Instrument

Name	ReactIR 700
	ReactIR 700; SN: C107148360; Detector: TEMCT; Apodization: Norton-Beer Medium; Probe: DiComp (Diamond); SN: C119744399; Interface: AgX 9.5mm x 1.5m Fiber (Silver Halide); Sampling: 3000 to 650 cm-1; Resolution: 8; Scan option: AutoSelect; Gain: Low;
Last Background	1/26/2024 11:17:41 AM

Figure S 1: Information of used measurement files, left of iC IR and right for iC Raman with specific experiment and instrument data.

Figure S 2 shows uncorrected data for Keggin tungsten at 10 °C synthesis and chosen reference spectrum of water under reaction conditions for a suitable solvent subtraction.

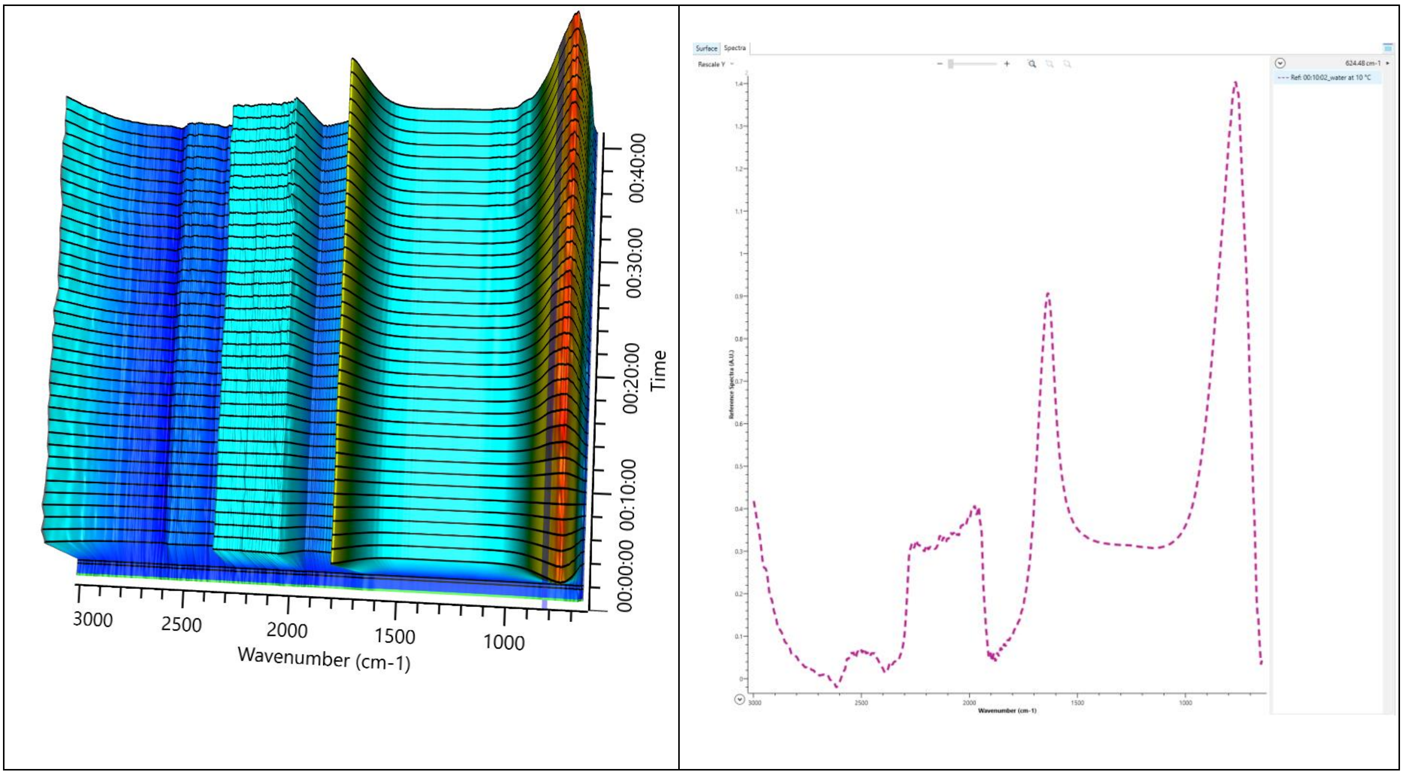

Figure S 2: Uncorrected surface plot for the whole experiment measured by IR (left) and chosen reference spectrum of water at reaction conditions at t = 10 min and T = 10 °C.

Solvent subtraction, or general subtraction can be performed using selected spectra. These can be chosen freely, but work best by choosing spectra, where the reaction conditions are set and no other compounds except the solvent are within the reaction solution (see Figure S 3).

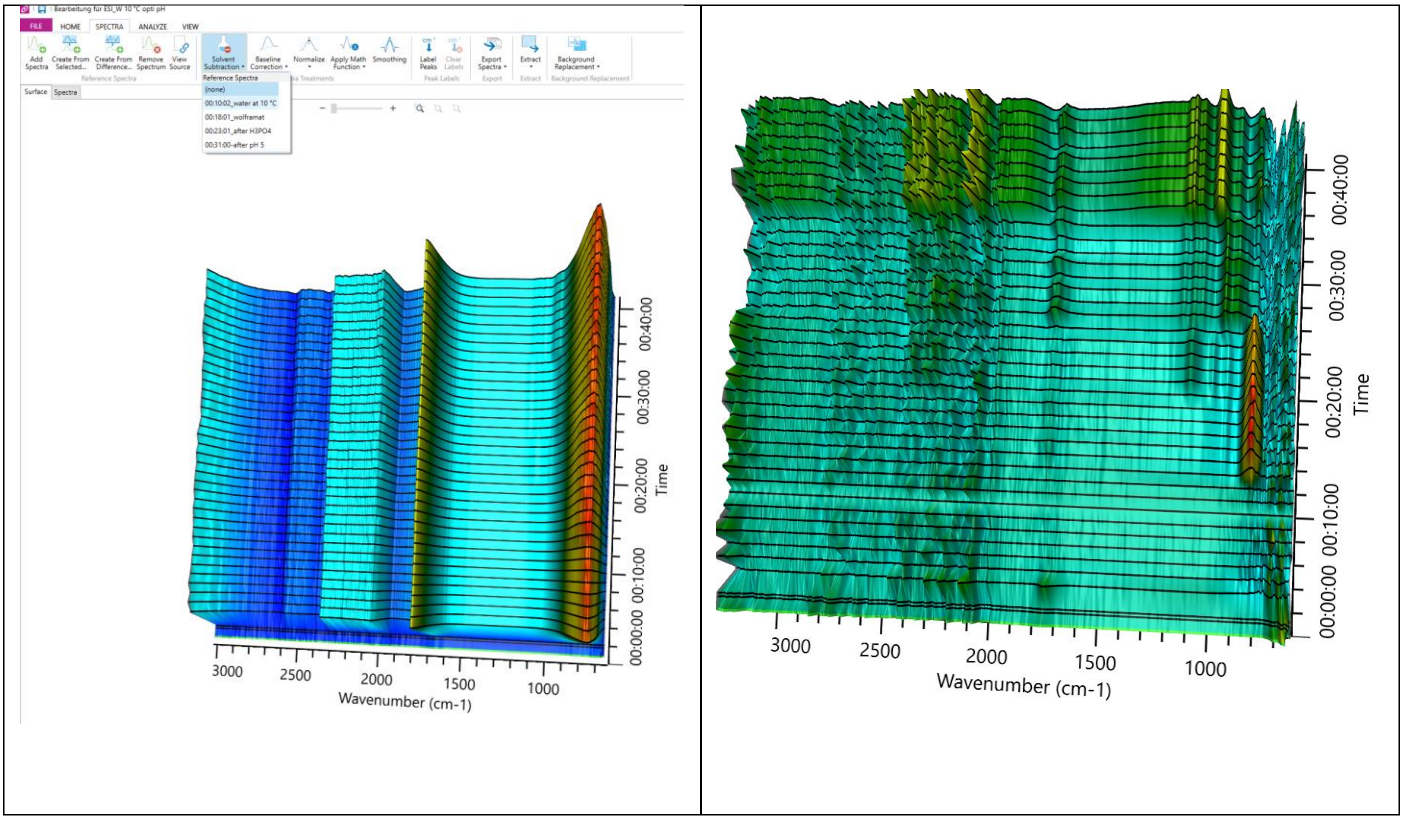

Figure S 3: Implementation of solvent subtraction (left) and resulting surface plot (right).

It is visible, that for this synthesis using inorganic materials the region above 1500 cm^{-1} is not relevant. However, the region below 800 cm^{-1} is not resolved perfectly leading to negative signals. Water is the main enemy in IR spectroscopic investigation, especially in the lower wavenumber region. Especially below 800 cm^{-1} (Figure S 4) and depending on related concentration negative effects can be found. The used algorithm “solvent subtraction” adjusts concentration of the subtracted spectrum to minimize negative peaks. However, the strong absorption of water in this region causes overestimation of its concentration leading to negative peaks. Since water is just an overlapping signal, with the actually relevant signal still present, iC IR software is still capable to measure the peak intensity for the relevant peaks through referencing a baseline within the peak definition (type height vs area, start/stop wavenumber for integration, left/right baseline points).

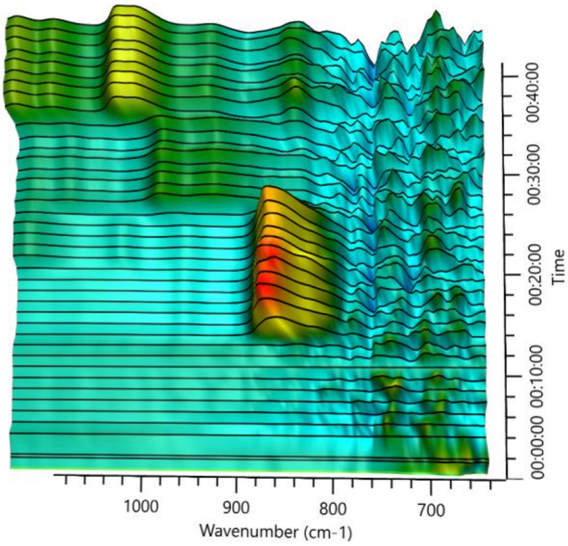

Figure S 4: Zoom in into solvent subtracted surface plot.

A way to optimize this, is using the baseline correction. Here, a baseline offset can be chosen from a specific spectrum, the procedure can be seen in Figure S 5.

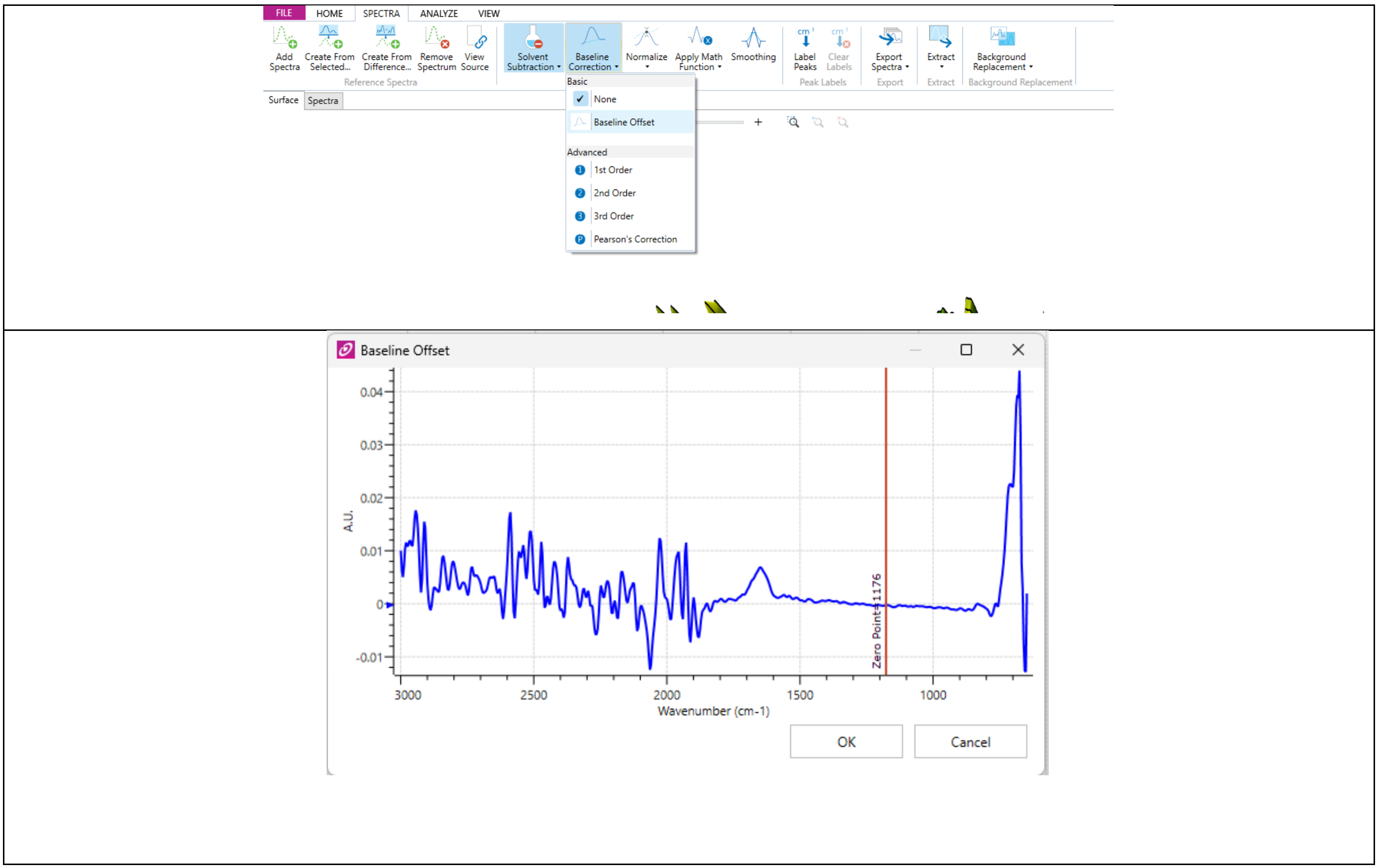

Figure S 5: Procedure for setting baseline offset in iC IR software.

The resulting spectra can be found Figure S 6.

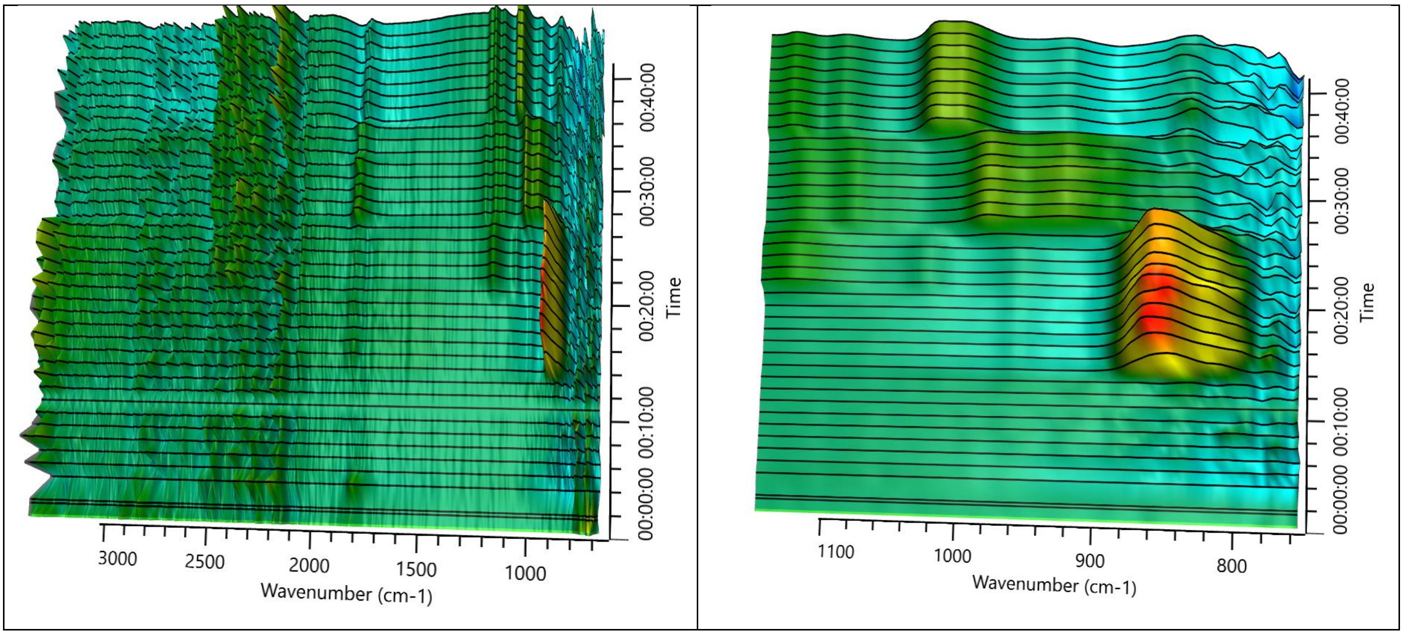

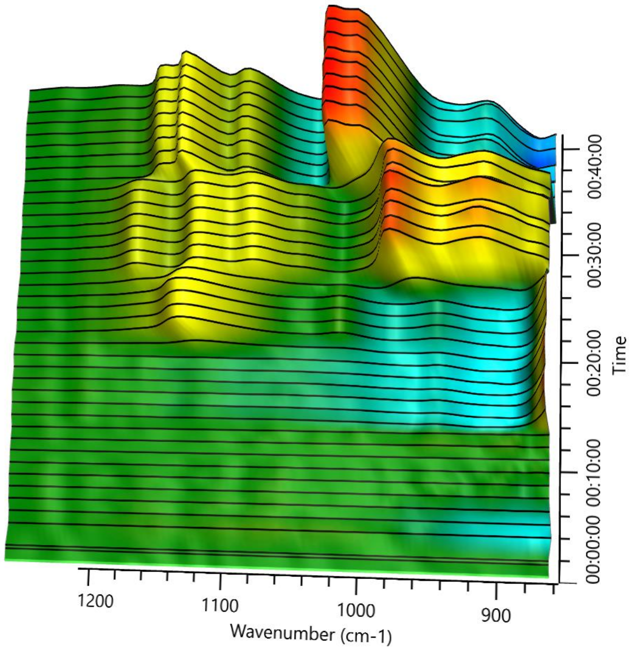

Figure S 6: Resulting spectra of baseline correction (top left) and zoom-in into specific regions of interest (top right and bottom). The software scales signals automatically within surface graphs for easier observation.

For further data processing, we chose important peaks for time-resolved investigations. In the following an exemplary selection of one peak is shown (see Figure S 7).

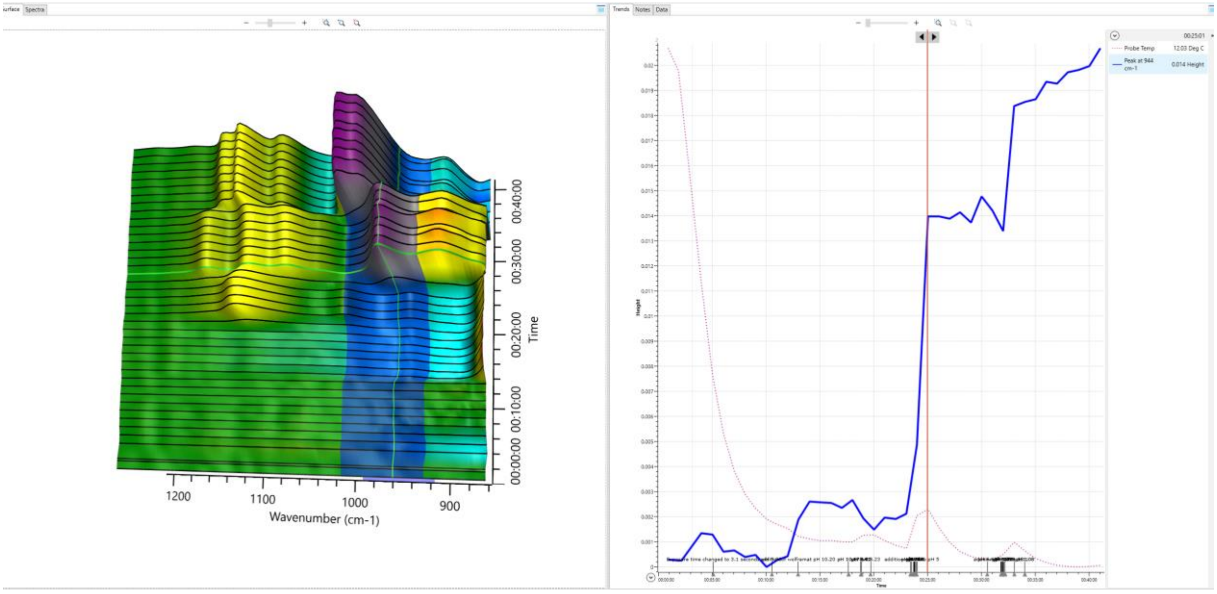

Figure S 7: Choosing of a signal and automated time-resolved plot for chosen signal.

As this signal is way too broad incorporating another signal of interest, the signal width was adjusted. For this, the spectra view was chosen (Figure S 8):

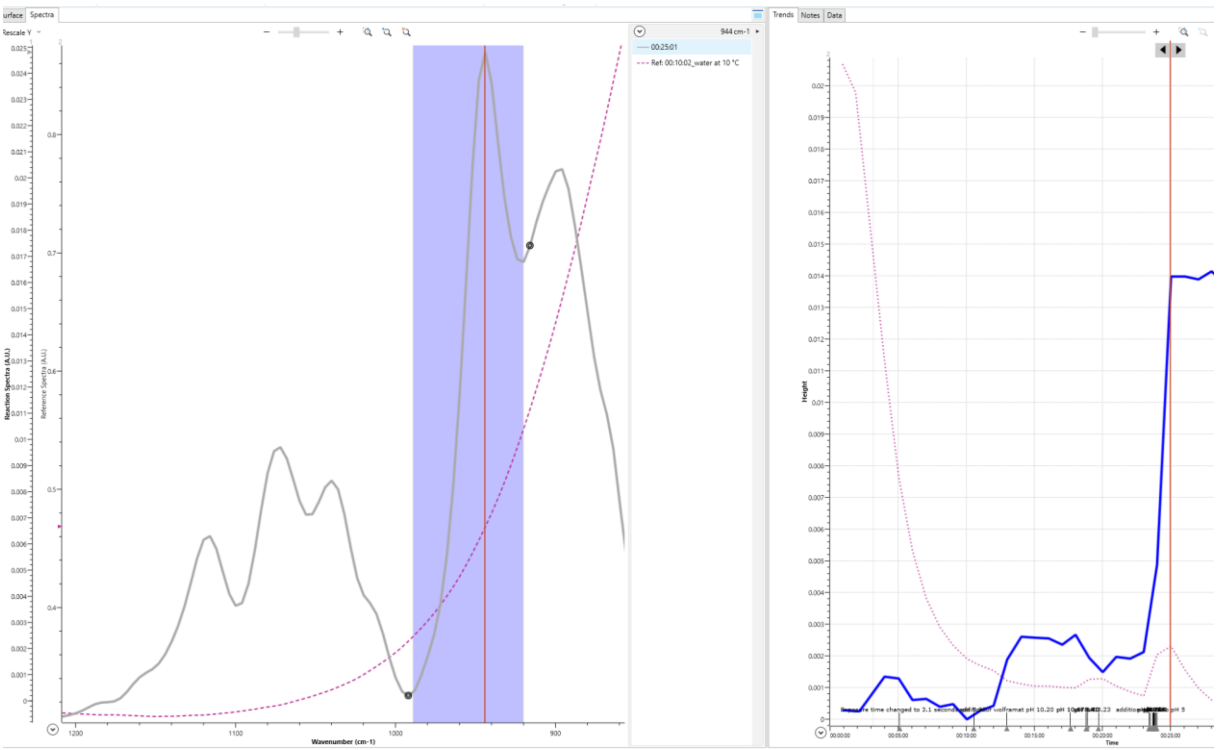

Figure S 8: Single spectra overview and resulting time-resolved plot.

For better interpretation, signal width (purple field) for automated trend observation needs to be adjusted by tuning selection either manually or automated (Figure S 9):

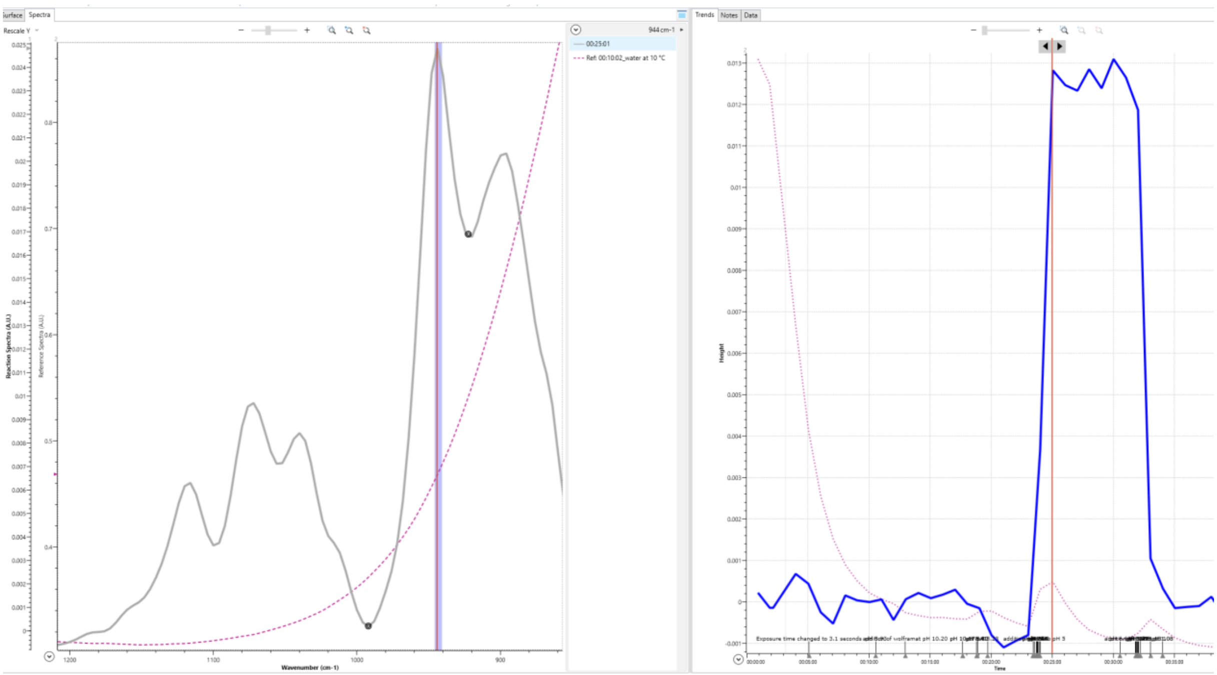

Figure S 9: Selected peak is specified and defined, resulting time-resolved plot shows an intermediate behaviour during synthesis.

This procedure was performed for all peaks within this study. Kinetic data was extracted from timed plots and calculated using the software Origin 2024 and Microsoft Excel.

Another important factor for POM synthesis is a careful investigation of pH values. Therefore, pH values were measured using a Lab 855 table device and a BlueLine 14 pH probe by SI Analytics®. Values were noted at various time intervals, depending on e.g. addition of acid or precursor. This can be seen exemplarily in

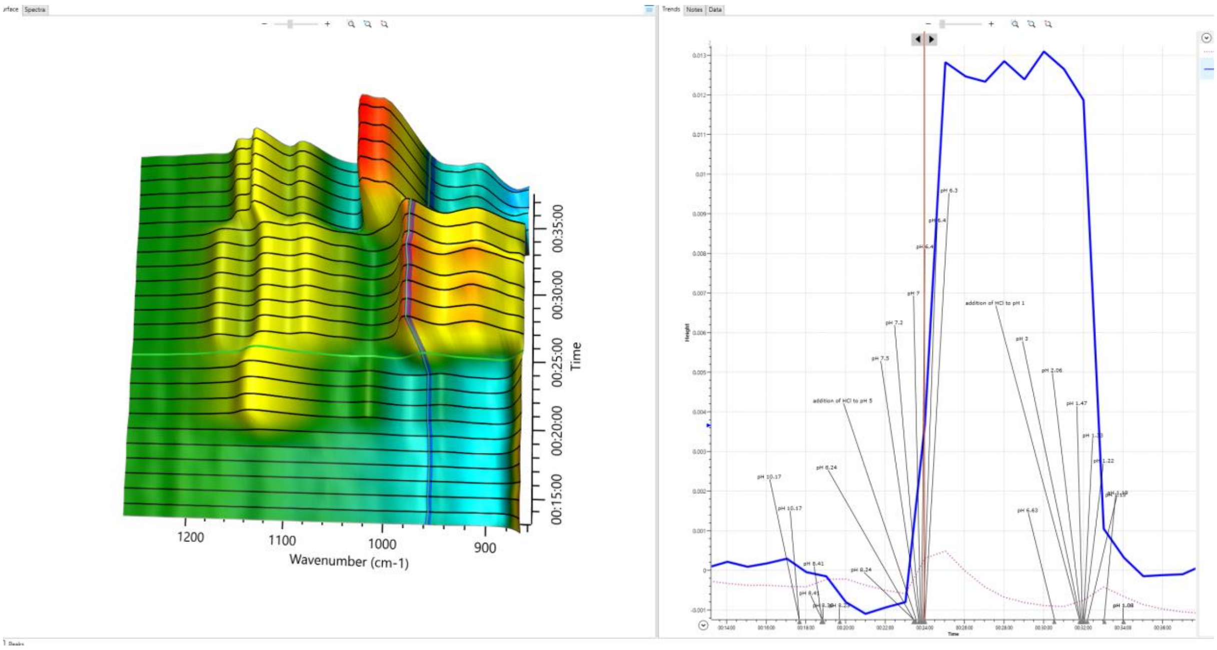

Figure S 10: Resulting surface and time-resolved plot including pH annotations.

Additionally, pH values were annotated directly at measured times into the raw data of the experiment (iC Control). The system automatically adds those annotations into the files of iC IR and iC Raman.

For Raman spectra, the procedure is quite similar. The raw, non-treated surface can be found in Figure S 11.

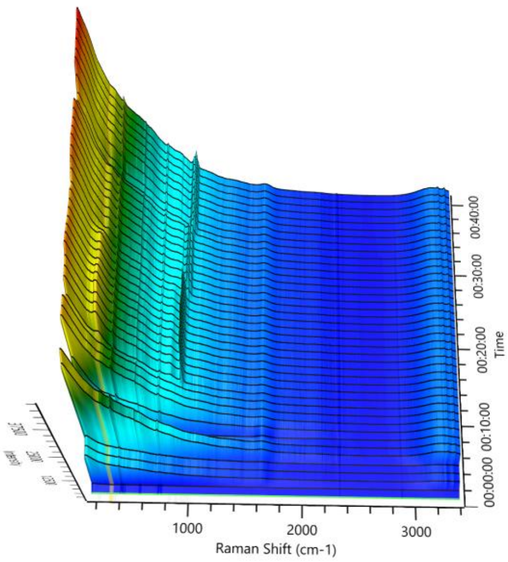

Figure S 11: Uncorrected Raman data for Keggin tungstate synthesis at 10 °C.

Firstly, the scatter correction was set to SNV (Figure S 12) resulting in a surface plot shown below.

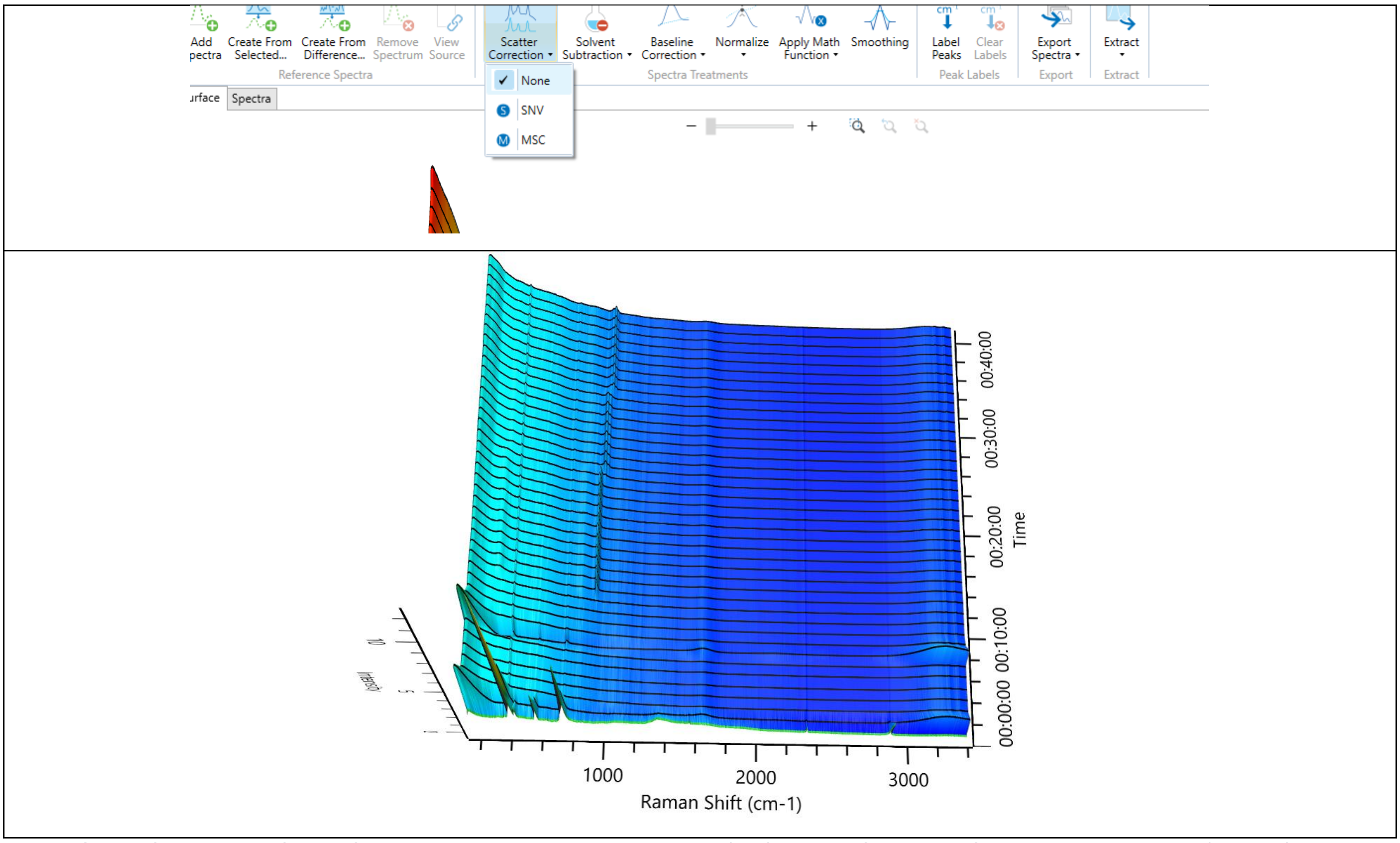

Figure S 12: iC Raman software for implementing scatter correction (top) and surface plot after scatter correction (bottom)

Further processing was performed similar to IR spectra by solvent subtraction of water at chosen reaction conditions (T = 10 °C). Procedure, water spectrum and resulting surface are shown in Figure S 13.

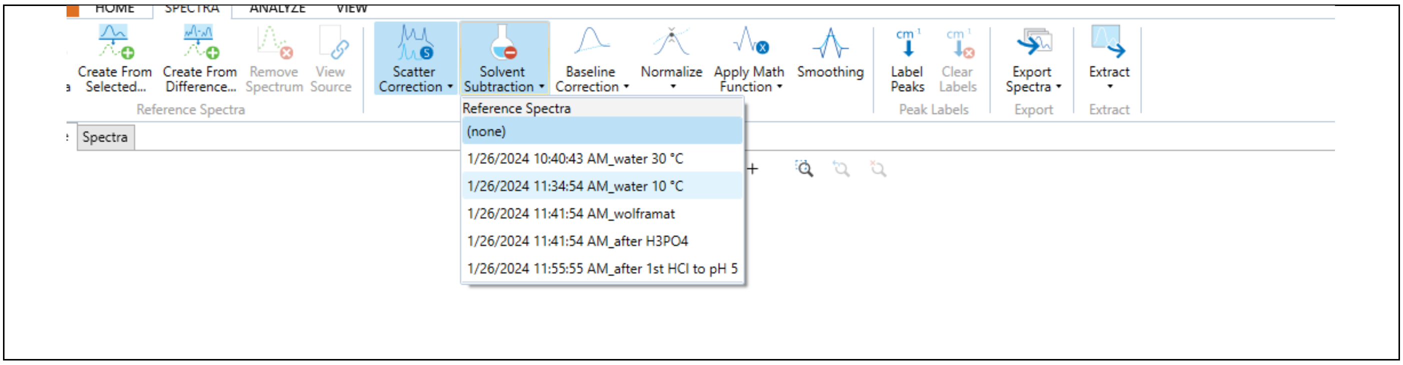

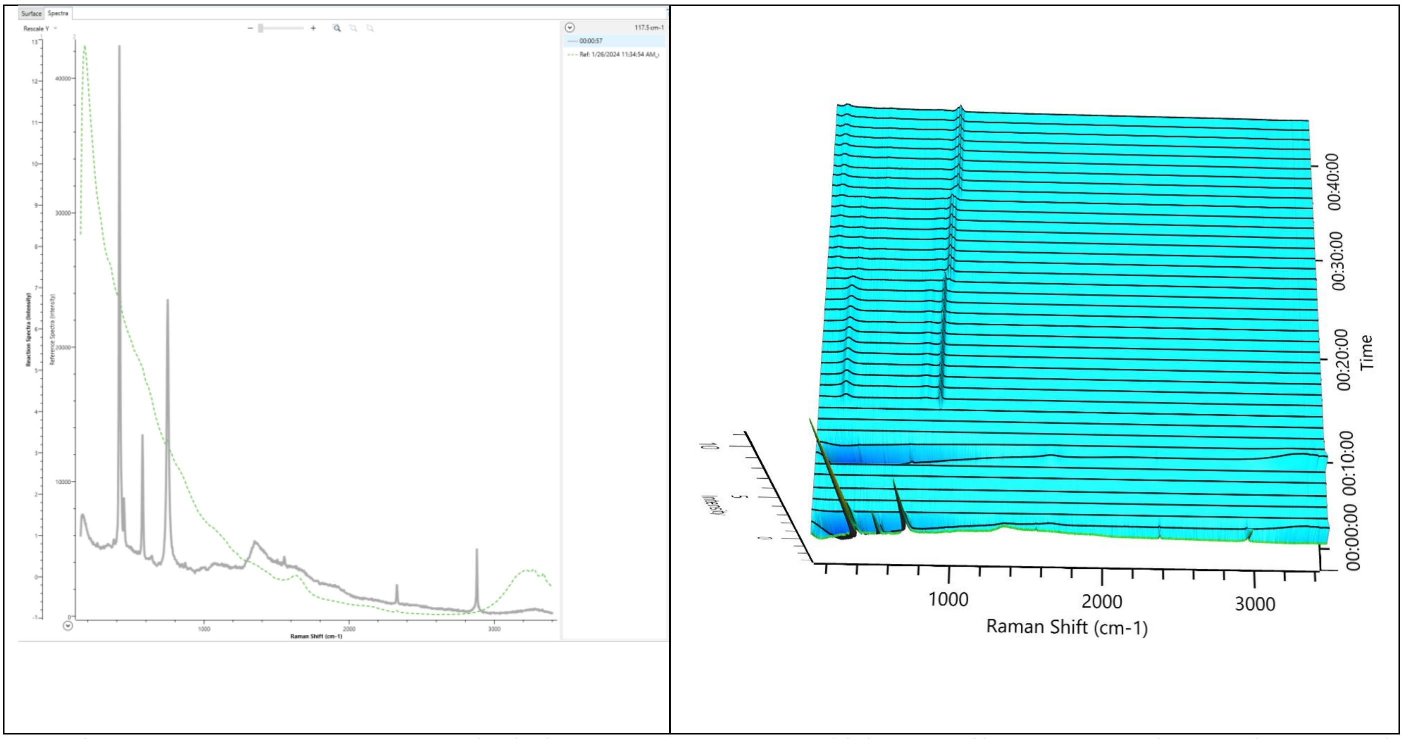

Figure S 13: Processing solvent subtraction (top) of water spectrum at T = 10 °C (bottom left) resulting in surface plot (bottom right).

Lastly, baseline correction was performed by choosing the active baseline correction treated method shown in Figure S 16.

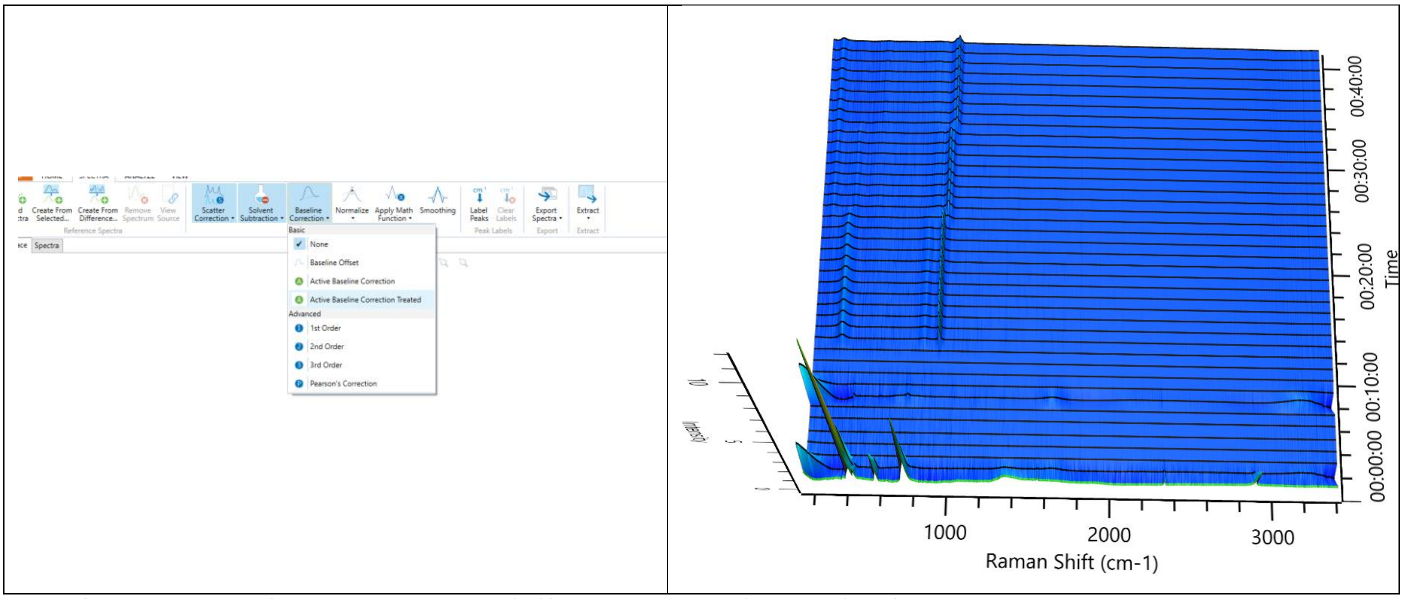

Figure S 14: Processing for baseline correction (left) and resulting surface plot (right).

Kinetic Data

Anderson-Evans Structure

Table S 1: Results of the ARRHENIUS analysis – based on the IR data – for the Anderson-Evans-type POM formation in aqueous solution.

Temperature [°C]	25	10	0
Temperature [K]	298.15	283.15	273.15
Reciprocal Temperature [1/K]	0.00335	0.00353	0.00366
ln(kR Peak 826 cm-1)			
kR Peak 826 cm-1 [mol/L min]	0.10745	0.09647	0.07724
ln(kR Peak 948 cm-1)			
kR Peak 948 cm-1 [mol/L min]	0.02200	0.01950	0.01597
Arrhenius parameter Peak 826 cm-1 [K]	-1047.1		
Arrhenius parameter Peak 948 cm-1 [K]	-1021.5		
Arrhenius parameter mean value [K]	-1034.3		
Activation energy Peak 826 cm-1 [kJ/mol]	8.4928		
Activation energy Peak 948 cm-1 [kJ/mol]	8.7056		
Activation energy (mean value) [kJ/mol]	8.5992		
ln(A) Peak 826 cm-1	1.3044		
A Peak 826 cm-1 [mol/L min]	3.6855		
ln(A) Peak 948 cm-1	-0.37251		
A Peak 948 cm-1 [mol/L min]	0.68900		

Wells-Dawson Structure

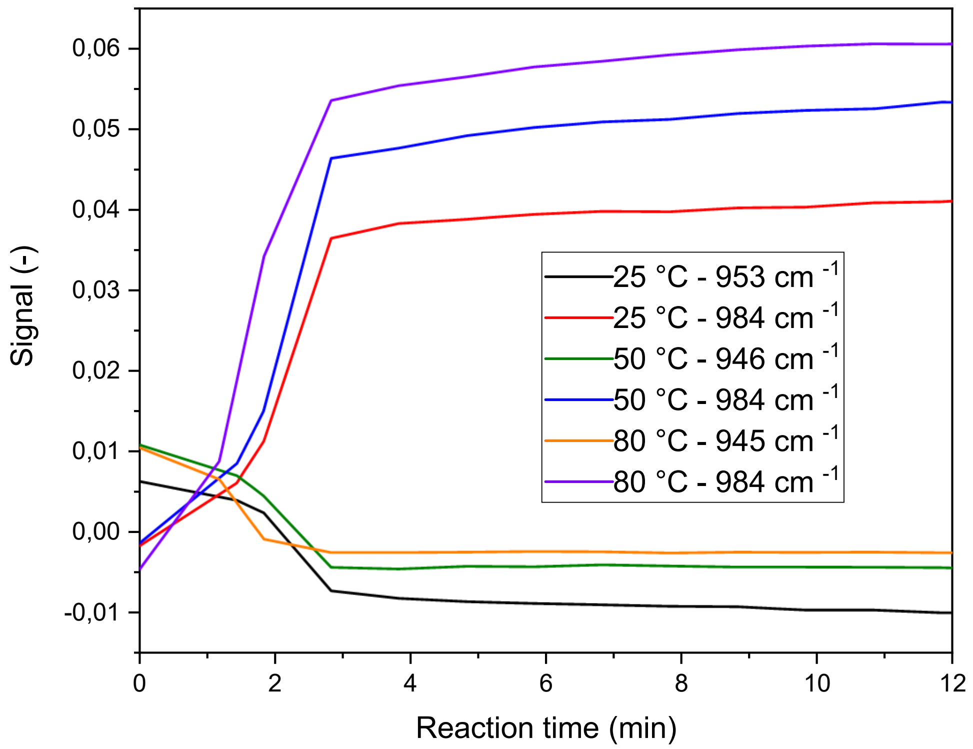

Figure S 15: Kinetic Data for the formation of Wells-Dawson structure @different tested reaction conditions.

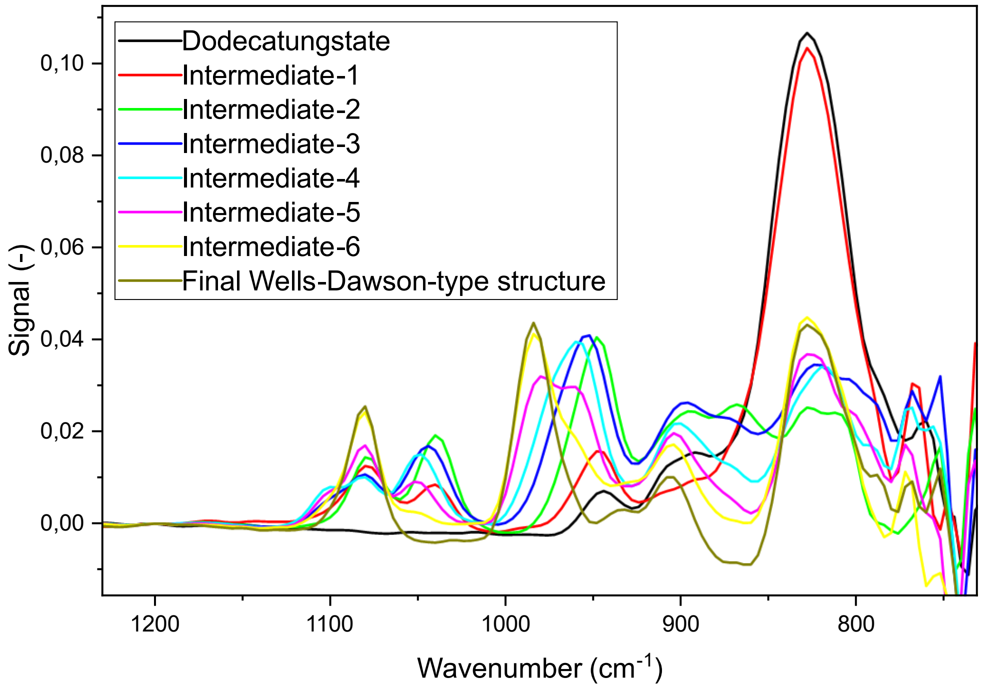

Figure S 16: Detailed IR spectra of Wells-Dawson structure formation @80 °C.

Table S 2: Results of the ARRHENIUS analysis – based on the IR data – for the Wells-Dawson-type POM formation in aqueous solution.

Temperature [°C]	80	50	25
Temperature [K]	353.15	323.15	298.15
Reciprocal Temperature [1/K]	0.00283	0.00309	0.00335
ln(k_R Peak 945, 946, 953 cm^{-1})	-5.1814	-4.7867	-4.8000
k_R Peak 945, 946, 953 cm^{-1} [ΔI/min]*	0.00562	0.00834	0.00823
ln(k_R Peak 984 cm^{-1})	-3.6687	-3.5353	-3.8117
k_R Peak 984 cm^{-1} [ΔI/min]*	0.02551	0.02915	0.02211
ARRHENIUS parameter Peak 945, 946, 953 cm^{-1} [K]	731.94		
ARRHENIUS parameter Peak 984 cm^{-1} [K]	-272.13		
ARRHENIUS parameter mean value [K]			
Activation energy Peak 945, 946, 953 cm^{-1} [kJ/mol]	-6.0853		
Activation energy Peak 984 cm^{-1} [kJ/mol]	2.2625		
Activation energy (mean value) [kJ/mol]			
ln(*A*) Peak 945, 946, 953 cm^{-1}	-7.1869		
A Peak 945, 946, 953 cm^{-1} [mol/L min]	0.0008		
ln(*A*) Peak 984 cm^{-1}	-2.8301		
A Peak 984 cm^{-1} [mol/L min]	0.0590		

* ΔI = difference in signal intensity

Keggin Structure

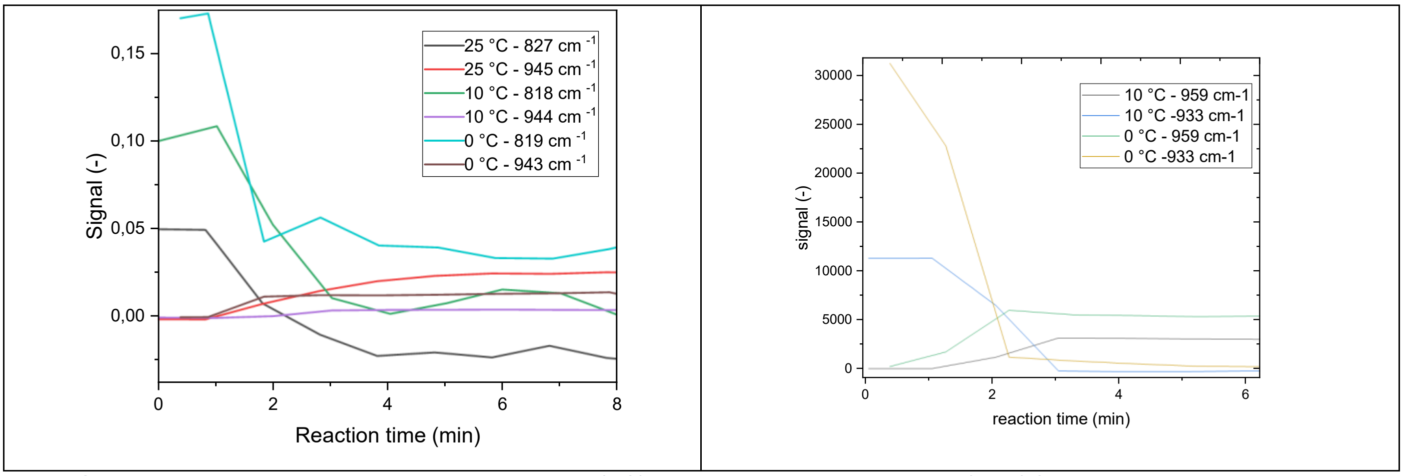

Figure S 17: Kinetic data measured by IR-spectroscopy (left) and by Raman-spectroscopy (right) for Keggin-tungstate **intermediate** (pH 5) formation.

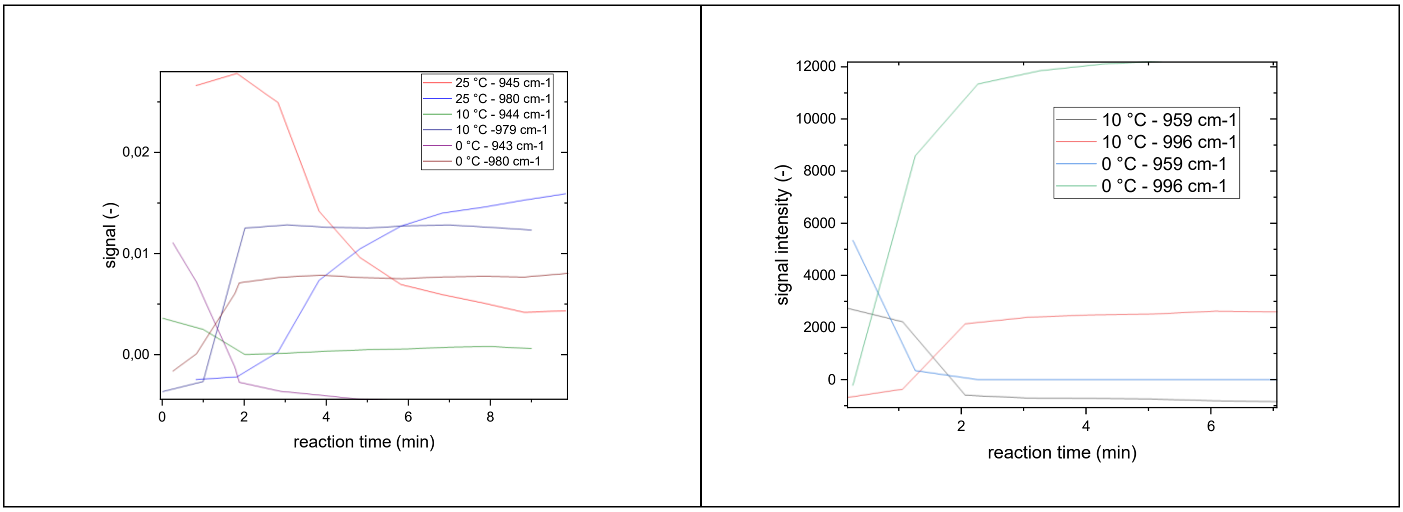

Figure S 18: Kinetic data measured by IR-spectroscopy (left) and by Raman-spectroscopy (right) for Keggin tungstate formation. (Starting from intermediate)

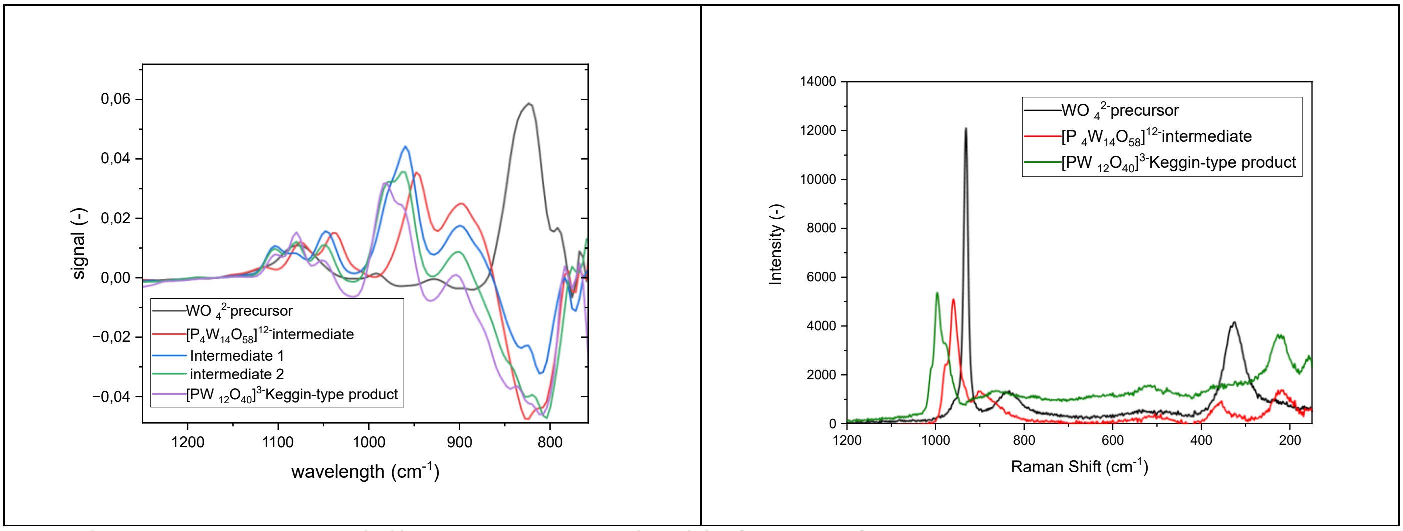

Figure S 19: Detailed IR-spectra (left) and Raman-spectra (right) for formation of Keggin structure tungstate

Table S 3: Results of the Arrhenius analysis – based on the IR data – for the $[P_4W_{14}O_{58}]^{12-}$ POM formation in aqueous solution.

Temperature [°C]	25	10	0
Temperature [K]	298.15	283.15	273.15
Reciprocal Temperature [1/K]	0.00335	0.00353	0.00366
ln(k_R Peak 819, 810, 827 cm^{-1})	-3.1734	-3.0239	-2.01335
k_R Peak 819, 810, 827 cm^{-1} [ΔI/min]*	0.04186	0.04861	0.13354
ln(k_R Peak 943, 944, 945 cm^{-1})	-5.09619	-6.13765	-4.99378
k_R Peak 943, 944, 945 cm^{-1} [ΔI/min]*	0.00612	0.00216	0.00678
ARRHENIUS parameter Peak 819, 810, 827 cm^{-1} [K]	3601.8		
ARRHENIUS parameter Peak 943, 944, 945 cm^{-1} [K]	-40.067		
ARRHENIUS parameter mean value [K]			
Activation energy Peak 819, 810, 827 cm^{-1} [kJ/mol]	-29.945		
Activation energy Peak 943, 944, 945 cm^{-1} [kJ/mol]	0.33312		
Activation energy (mean value) [kJ/mol]			
ln(*A*) Peak 819, 810, 827 cm^{-1}	-15.399		
A Peak 819, 810, 827 cm^{-1} [ΔI/min]*	$2.0526 \cdot 10^{-7}$		
ln(*A*) Peak 943, 944, 945 cm^{-1}	-5.2684		
A Peak 943, 944, 945 cm^{-1} [ΔI/min]*	0.00515		

Table S 4: Results of the Arrhenius analysis – based on the IR data – for the Keggin-type $[PW_{12}O_{40}]^{3-}$ POM formation in aqueous solution.

Temperature [°C]	25	10	0
Temperature [K]	298.15	283.15	273.15
Reciprocal Temperature [1/K]	0.00335	0.00353	0.00366
ln(k_R Peak 943, 944, 945 cm^{-1})	-5.1362	-6.2554	-4.7665
k_R Peak 943, 944, 945 cm^{-1} [ΔI/min]*	0.00588	0.00192	0.00851
ln(k_R Peak 979, 980 cm^{-1})	-5.3861	-4.2295	-5.2048
k_R Peak 979, 980 cm^{-1} [ΔI/min]*	0.00458	0.01456	0.00549
ARRHENIUS parameter Peak 943, 944, 945 cm^{-1} [K]	947.41		
ARRHENIUS parameter Peak 979, 980 cm^{-1} [K]	751.66		
ARRHENIUS parameter mean value [K]			
Activation energy Peak 943, 944, 945 cm^{-1} [kJ/mol]	-7.8768		
Activation energy Peak 979, 980 cm^{-1} [kJ/mol]	-6.2493		
Activation energy (mean value) [kJ/mol]			
ln(*A*) Peak 943, 944, 945 cm^{-1}	-8.2708		
A Peak 943, 944, 945 cm^{-1} [ΔI/min]*	0.00026		
ln(*A*) Peak 979, 980 cm^{-1}	-8.0286		
A Peak 979, 980 cm^{-1} [[ΔI/min]*	0.00033		

Table S 5: Results of the Arrhenius analysis – based on the Raman data – for the $[P_4W_{14}O_{58}]^{12-}$ intermediate formation in aqueous solution.

Temperature [°C]	10	0
Temperature [K]	283.15	273.15
Reciprocal Temperature [1/K]	0.00353	0.00366
ln(k_R Peak 931 cm^{-1})	8.6569	9.7065
k_R Peak 931 cm^{-1} [ΔI/min]*	5749.7	16424
ln(k_R Peak 959 cm^{-1})	7.3515	8.0578
k_R Peak 959 cm^{-1} [ΔI/min]*	1558.6	3158.4
ARRHENIUS parameter Peak 931 cm^{-1} [K]	8117.8	
ARRHENIUS parameter Peak 959 cm^{-1} [K]	5462.5	
ARRHENIUS parameter mean value [K]		
Activation energy Peak 931 cm^{-1} [kJ/mol]	-67.491	
Activation energy Peak 959 cm^{-1} [kJ/mol]	-45.415	
Activation energy (mean value) [kJ/mol]		
ln(*A*) Peak 931 cm^{-1}	-20.013	
A Peak 931 cm^{-1} [ΔI/min]*	$2.03 \cdot 10^{-9}$	
ln(*A*) Peak 959 cm^{-1}	-11.940	
A Peak 959 cm^{-1} [ΔI/min]*	$6.52 \cdot 10^{-6}$	

Table S 6: Results of the Arrhenius analysis – based on the Raman data – for the $[PW_{12}O_{40}]^{3-}$ Keggin-type POM formation in aqueous solution.

Temperature [°C]	10	0
Temperature [K]	283.15	273.15
Reciprocal Temperature [1/K]	0.00353	0.00366
ln(k_R Peak 996 cm^{-1})	8.6569	9.7065
k_R Peak 996 cm^{-1} [ΔI/min]*	5749.7	16424
ARRHENIUS parameter Peak 996 cm^{-1} [K]	79104	
Activation energy Peak 996 cm^{-1} [kJ/mol]	-657.67	
ln(*A*) Peak 996 cm^{-1}	-280.87	
A Peak 996 cm^{-1} [ΔI/min]*	$1.05 \cdot 10^{-122}$	

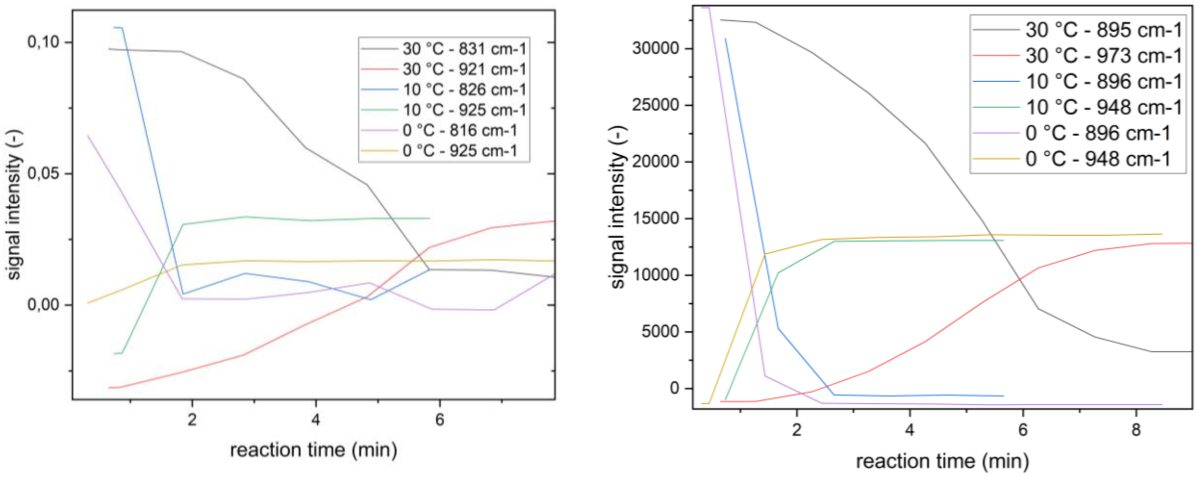

Figure S 21: Kinetic data measured by IR-spectroscopy (left) and by Raman-spectroscopy (right) for Keggin-molybdate **intermediate** (pH 5) formation.

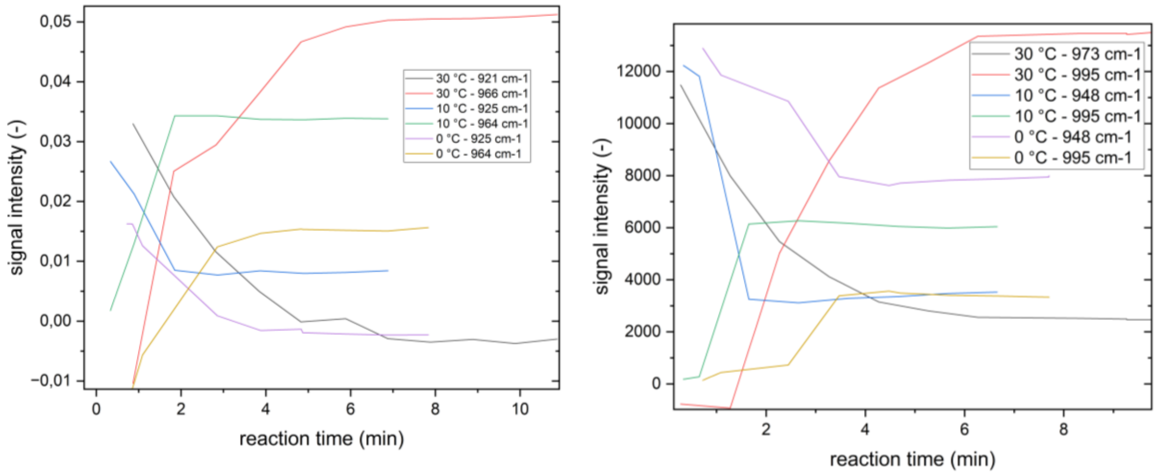

Figure S 20: Kinetic data measured by IR-spectroscopy (left) and by Raman-spectroscopy (right) for Keggin-molybdate formation. (Starting from intermediate)

Table S 7: Results of the Arrhenius analysis – based on the IR data – for the molybdenum based intermediate at pH 5 in aqueous solution.

Temperature [°C]	30	10	0
Temperature [K]	303.15	283.15	273.15
Reciprocal Temperature [1/K]	0.00330	0.00353	0.00366
ln(kR Peak 831, 826, 816 cm-1)	-3.8561	-2.2714	-3.2001
kR Peak 831, 826, 816 cm-1 [ΔI/min]*	0.02115	0.10317	0.04076
ln(kR Peak 921, 925 cm-1)	-4.5310	-2.9973	-4.6523
kR Peak 921, 925 cm-1 [ΔI/min]*	0.01077	0.04992	0.00954
Arrhenius parameter Peak 819, 810, 827 cm-1 [K]	2407.0		
Arrhenius parameter Peak 943, 944, 945 cm-1 [K]	491.60		
Arrhenius parameter mean value [K]			
Activation energy Peak 819, 810, 827 cm-1 [kJ/mol]	-20.012		
Activation energy Peak 943, 944, 945 cm-1 [kJ/mol]	-4.0872		
Activation energy (mean value) [kJ/mol]			
ln(A) Peak 819, 810, 827 cm-1	-11.527		
A Peak 819, 810, 827 cm-1 [ΔI/min]*	9.86 · 10-6		
ln(A) Peak 943, 944, 945 cm-1	-5.7794		
A Peak 943, 944, 945 cm-1 [ΔI/min]*	0.00309		

Table S 8: Results of the Arrhenius analysis – based on the Raman data – for the $[PMo_{12}O_{40}]^{3-}$ Keggin-type formation at pH 1 in aqueous solution.

Temperature [°C]	30	10	0
Temperature [K]	303.15	283.15	273.15
Reciprocal Temperature [1/K]	0.00330	0.00353	0.00366
ln(kR Peak 921, 925 cm-1)	-4.5347	-4.4363	-4.9036
kR Peak 921, 925 cm-1 [ΔI/min]*	0.01073	0.01184	0.00742
ln(kR Peak 966, 964 cm-1)	-3.5453	-3.8476	-4.5592
kR Peak 966, 964 cm-1 [ΔI/min]*	0.02886	0.02133	0.01047
Arrhenius parameter Peak 921, 925 cm-1 [K]	-846.03		
Arrhenius parameter Peak 966, 964 cm-1 [K]	-2619.3		
Arrhenius parameter mean value [K]			
Activation energy Peak 921, 925 cm-1 [kJ/mol]	7.0340		
Activation energy Peak 966, 964 cm-1 [kJ/mol]	21.777		
Activation energy (mean value) [kJ/mol]			
ln(A) Peak 921, 925 cm-1	-1.6662		
A Peak 921, 925 cm-1 [ΔI/min]*	0.18900		
ln(A) Peak 966, 964 cm-1	-5.1761		
A Peak 966, 964 cm-1 [ΔI/min]*	0.00565		

Table S 9: Results of the Arrhenius analysis – based on the Raman data – for the molybdenum based intermediate at pH 5 in aqueous solution.

Temperature [°C]	30	10	0
Temperature [K]	303.15	283.15	273.15
Reciprocal Temperature [1/K]	0.00330	0.00353	0.00366
ln(k_R Peak 896 cm^{-1})	8.8934	10.217	10.395
k_R Peak 896 cm^{-1} [ΔI/min]*	7283.6	27372	32708
ln(k_R Peak 948, 973 cm^{-1})	7.9428	9.3905	9.4938
k_R Peak 948, 973 cm^{-1} [ΔI/min]*	2815.1	11974	13277
ARRHENIUS parameter Peak 896 cm^{-1} [K]	4329.3		
ARRHENIUS parameter Peak 948, 973 cm^{-1} [K]	4511.9		
ARRHENIUS parameter mean value [K]			
Activation energy Peak 896 cm^{-1} [kJ/mol]	-35.993		
Activation energy Peak 948, 973 cm^{-1} [kJ/mol]	-37.512		
Activation energy (mean value) [kJ/mol]			
ln(A) Peak 896 cm^{-1}	-5.3046		
A Peak 896 cm^{-1} [ΔI/min]*	0.00500		
ln(A) Peak 948, 973 cm^{-1}	-6.8364		
A Peak 948, 973 cm^{-1} [ΔI/min]*	0.00107		

Table S 10: Results of the Arrhenius analysis – based on the Raman data – for the $[PMo_{12}O_{40}]^{3-}$ Keggin-type formation at pH 1 in aqueous solution.

Temperature [°C]	30	10	0
Temperature [K]	303.15	283.15	273.15
Reciprocal Temperature [1/K]	0.00330	0.00353	0.00366
ln(k_R Peak 948, 973 cm^{-1})	8.0379	9.0556	7.9496
k_R Peak 948, 973 cm^{-1} [ΔI/min]*	3096.2	8566.3	2834.3
ln(k_R Peak 995 cm^{-1})	8.3144	8.6647	7.8627
k_R Peak 995 cm^{-1} [ΔI/min]*	4082.4	5794.4	2598.5
ARRHENIUS parameter Peak 948, 973 cm^{-1} [K]	306.97		
ARRHENIUS parameter Peak 995 cm^{-1} [K]	-918.39		
ARRHENIUS parameter mean value [K]			
Activation energy Peak 948, 973 cm^{-1} [kJ/mol]	-2.5522		
Activation energy Peak 995 cm^{-1} [kJ/mol]	7.6355		
Activation energy (mean value) [kJ/mol]			
ln(A) Peak 948, 973 cm^{-1}	7.2742		
A Peak 948, 973 cm^{-1} [ΔI/min]*	1442.6		
ln(A) Peak 995 cm^{-1}	11.492		
A Peak 995 cm^{-1} [ΔI/min]*	97929		

Synthesis of $[PV_2Mo_{10}O_{40}]^{5-}$ via lacunary route

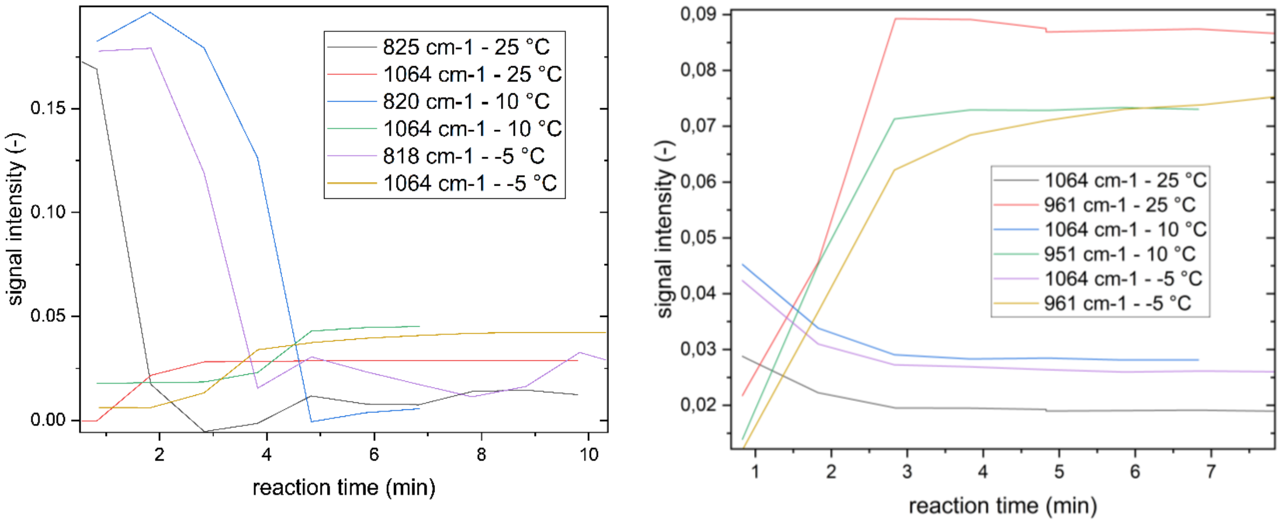

Figure S 22: Kinetic data for Lacunary formation kinetics (left) and Keggin Formation from Lacunary (right).

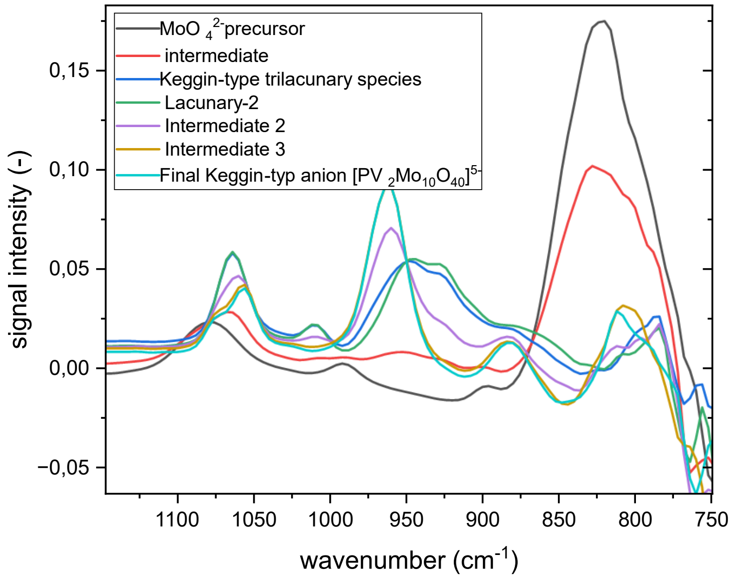

Figure S 23: IR spectra for Substitution experiment using the Lacunary Approach @10 °C

Table S 11: Results of the Arrhenius analysis – based on the IR data – for the Keggin-Lacunary-type $[PMo_9O_{34}]^{9-}$ in aqueous solution.

Temperature [°C]	25	10	-5
Temperature [K]	298.15	283.15	268.15
Reciprocal Temperature [1/K]	0.00335	0.00353	0.00373
ln(k_R Peak 818, 820 and 825 cm^{-1})	-1.8959	-2.0643	-2.5548
k_R Peak 818, 820 and 825 cm^{-1} [ΔI/min]*	0.15018	0.12691	0.07771
ln(k_R Peak 1064 cm^{-1})	-4.2426	-3.9145	-4.3788
k_R Peak 1064 cm^{-1} [ΔI/min]*	0.01437	0.01995	0.01254
ARRHENIUS parameter Peak 818, 820 and 825 cm^{-1} [K]	-1769.3		
ARRHENIUS parameter Peak 1064 cm^{-1} [K]	-399.93		
ARRHENIUS parameter mean value [K]			
Activation energy Peak 818, 820 and 825 cm^{-1} [kJ/mol]	16.479		
Activation energy Peak 1064 cm^{-1} [kJ/mol]	3.3250		
Activation energy (mean value) [kJ/mol]			
ln(A) Peak 818, 820 and 825 cm^{-1}	4.0888		
A Peak 818, 820 and 825 cm^{-1} [ΔI/min]*	59.668		
ln(A) Peak 1064 cm^{-1}	-2.7636		
A Peak 1064 cm^{-1} [ΔI/min]*	0.06306		

Table S 12: Results of the Arrhenius analysis for the $[PV_2Mo_{10}O_{40}]^{5-}$ Keggin-type formation in aqueous solution.

Temperature [°C]	25	10	-5
Temperature [K]	298.15	283.15	268.15
Reciprocal Temperature [1/K]	0.00335	0.00353	0.00373
ln(k_R Peak 1064 cm^{-1})	-5.0452	-4.4724	-4.4777
k_R Peak 1064 cm^{-1} [ΔI/min]*	0.00644	0.01142	0.01136
ln(k_R Peak 951 and 961 cm^{-1})	-3.4311	-3.5491	-3.6869
k_R Peak 951 and 961 cm^{-1} [ΔI/min]*	0.03235	0.02875	0.02505
ARRHENIUS parameter Peak 1064 cm^{-1} [K]	1484.0		
ARRHENIUS parameter Peak 951 and 961 cm^{-1} [K]	-681.83		
ARRHENIUS parameter mean value [K]			
Activation energy Peak 1064 cm^{-1} [kJ/mol]	-12.338		
Activation energy Peak 951 and 961 cm^{-1} [kJ/mol]	5.6687		
Activation energy (mean value) [kJ/mol]			
ln(A) Peak 1064 cm^{-1}	-9.9158		
A Peak 1064 cm^{-1} [ΔI/min]*	0.0000494		
ln(A) Peak 951 and 961 cm^{-1}	-1.1432		
A Peak 951 and 961 cm^{-1} [ΔI/min]*	0.31880		

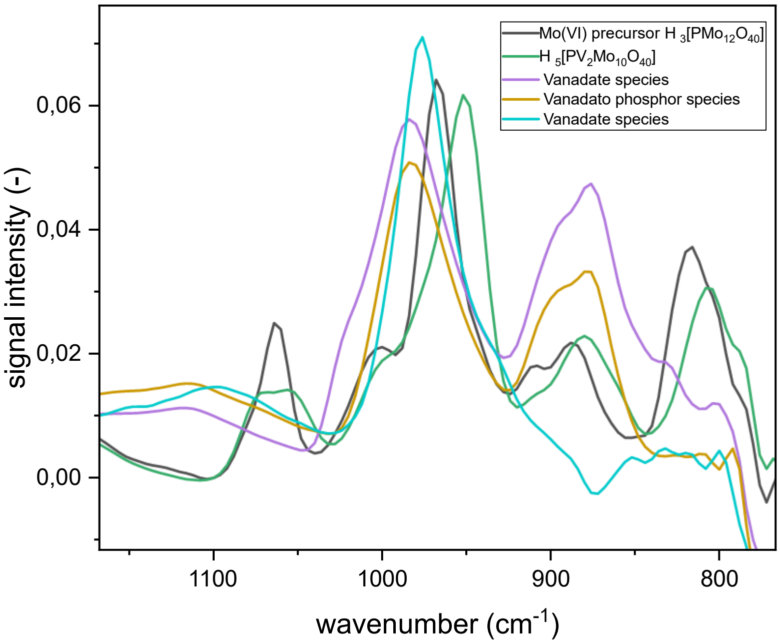

Figure S 24: Detailed IR spectra for synthesis of vanadium substituted Keggin Phosphomolybdate

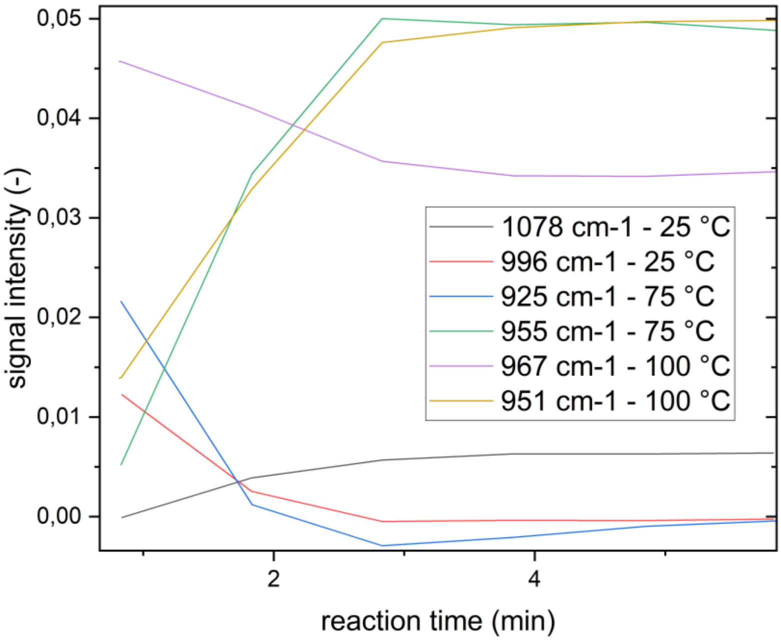

Figure S 25: Kinetic evaluation determined by IR spectroscopy for self-assembly formation.

Table S 13: Results of the Arrhenius analysis – based on the IR data – for the self-assembly of anion $[PV_2Mo_{10}O_{40}]^{5-}$ in aqueous solution.

Temperature [°C]	100	75	25
Temperature [K]	373.15	348.15	298.15
Reciprocal Temperature [1/K]	0.00268	0.00287	0.00335
ln(k_R Peak 948, 973 cm^{-1})	-5.3104	-3.8966	-4.6295
k_R Peak 948, 973 cm^{-1} [ΔI/min]*	0.00494	0.02031	0.00976
ln(k_R Peak 995 cm^{-1})	-4.0699	-3.7951	-5.8396
k_R Peak 995 cm^{-1} [ΔI/min]*	0.01708	0.02248	0.00291
ARRHENIUS parameter Peak 948, 973 cm^{-1} [K]	522.59		
ARRHENIUS parameter Peak 995 cm^{-1} [K]	-2937.1		
ARRHENIUS parameter mean value [K]			
Activation energy Peak 948, 973 cm^{-1} [kJ/mol]	-4.3448		
Activation energy Peak 995 cm^{-1} [kJ/mol]	24.419		
Activation energy (mean value) [kJ/mol]			
ln(A) Peak 948, 973 cm^{-1}	-6.1636		
A Peak 948, 973 cm^{-1} [ΔI/min]*	0.00210		
ln(A) Peak 995 cm^{-1}	4.1512		
A Peak 995 cm^{-1} [ΔI/min]*	63.510		

Spectroscopic Investigations (solid state)

Anderson Evans

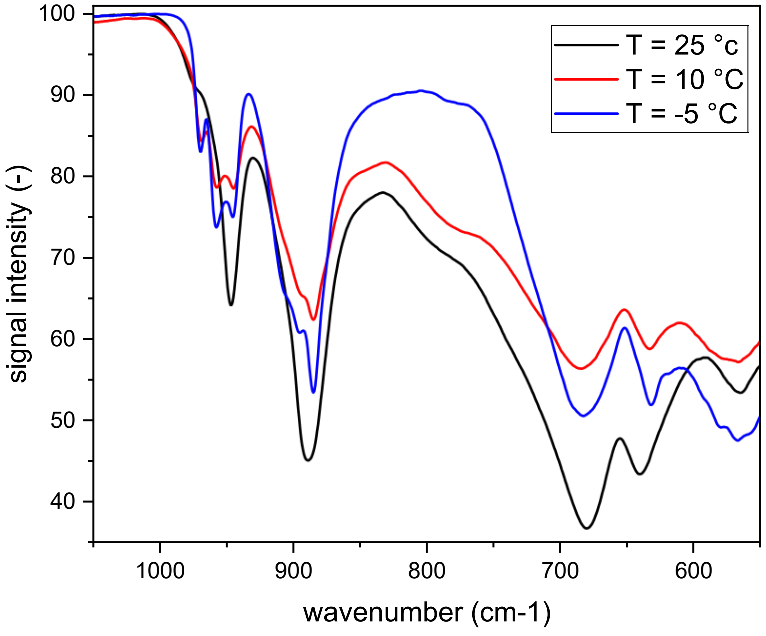

Figure S 26:IR-spectra of synthesized Anderson Evans structures (solid state)

Wells-Dawson

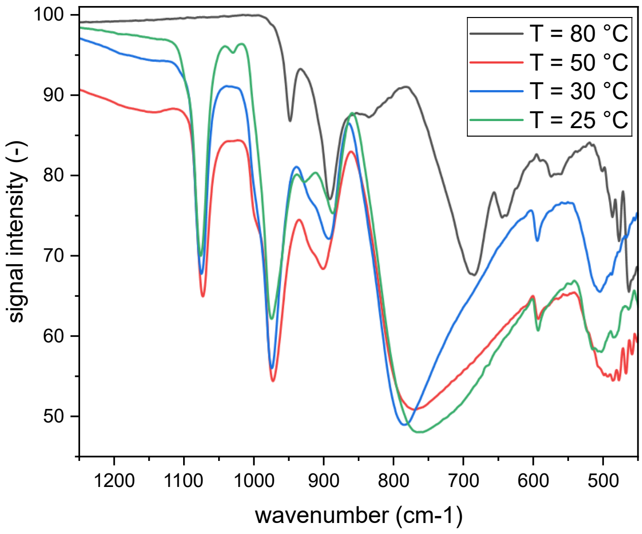

Figure S 27: Solid state IR-spectra of synthesized Wells-Dawson structures.

Keggin tungstate

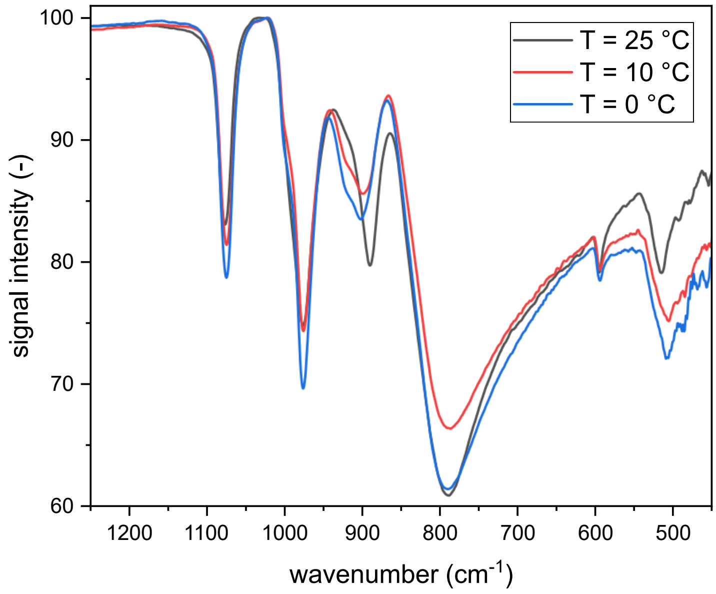

Figure S 28: Solid state IR-spectra of synthesized Keggin tungstate samples

Keggin molybdate

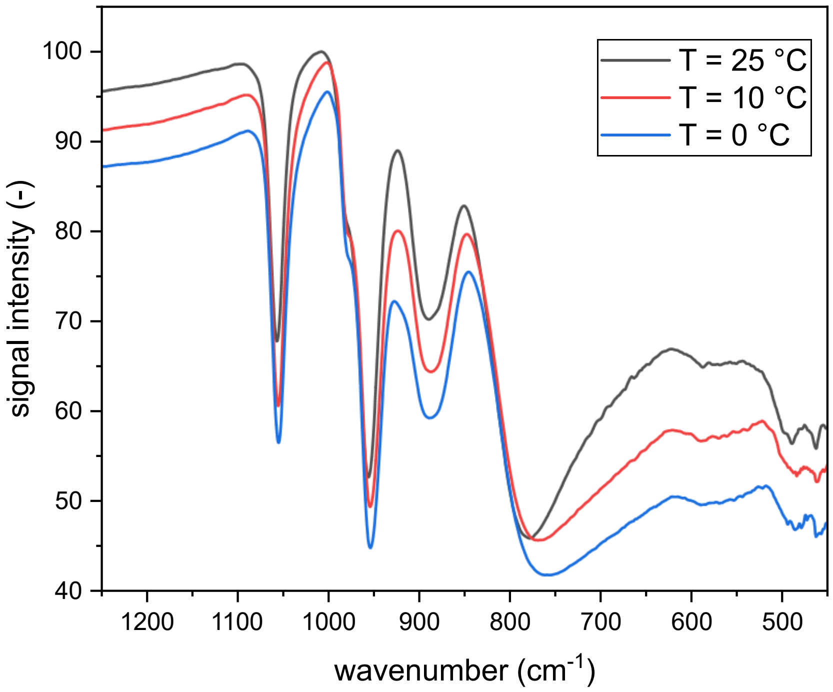

Figure S 29: Solid state IR-spectra of synthesized Keggin molybdate samples.

$[PV_2Mo_{10}O_{40}]^{5-}$ via self-assembly

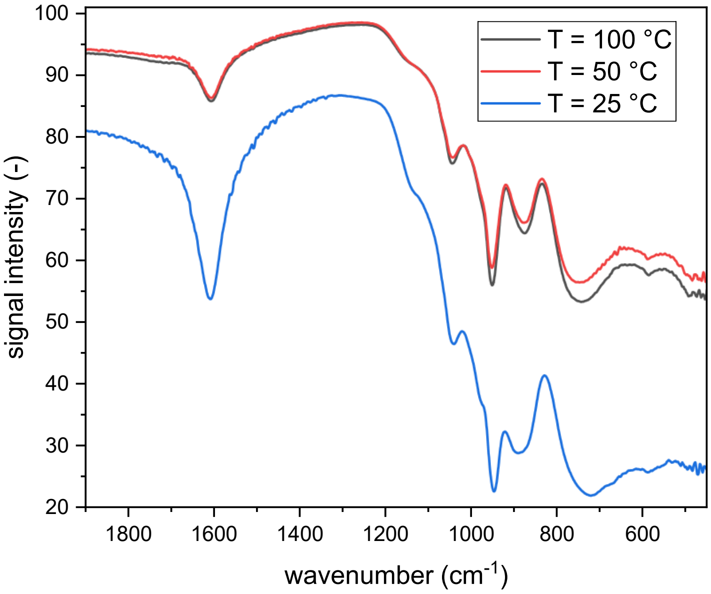

Figure S 30: Solid state IR-spectra of vanadium substituted Keggin molybdate synthesis by self-assembly.

$[PV_2Mo_{10}O_{40}]^{5-}$ via lacunary route

lacunary route

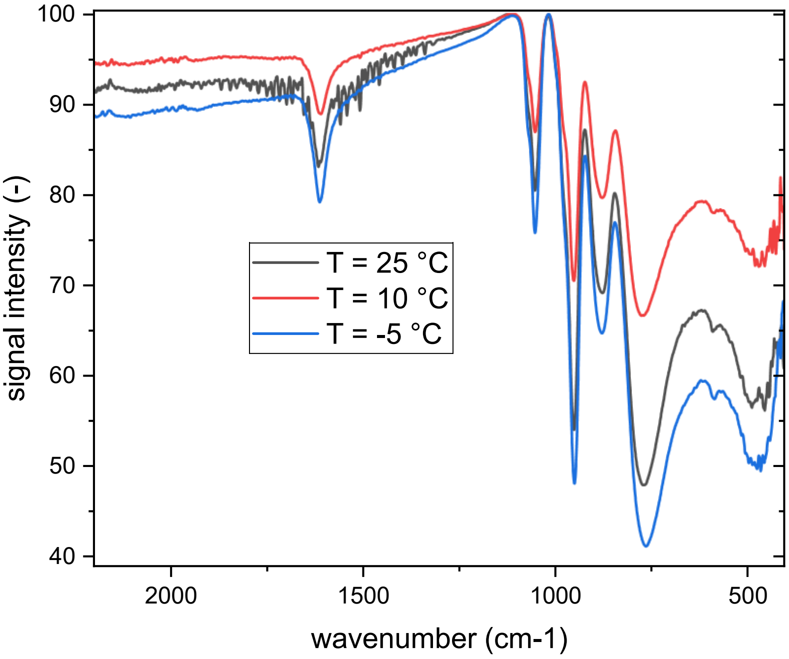

Figure S 31: Solid state IR-spectra of vanadium substituted Keggin molybdate synthesis by lacunary approach.

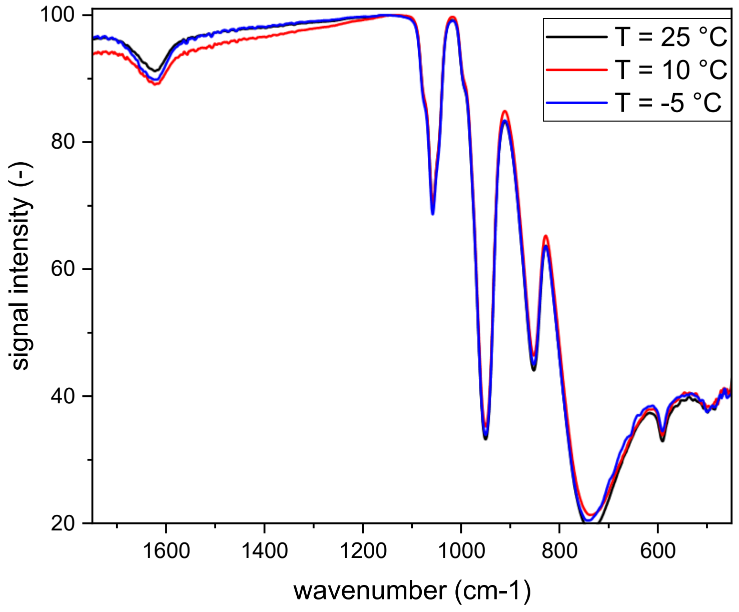

Figure S 32: Cs-precipitation of Lacunary approach synthesis.

Spectroscopic Investigations (liquid state)

Wells-Dawson

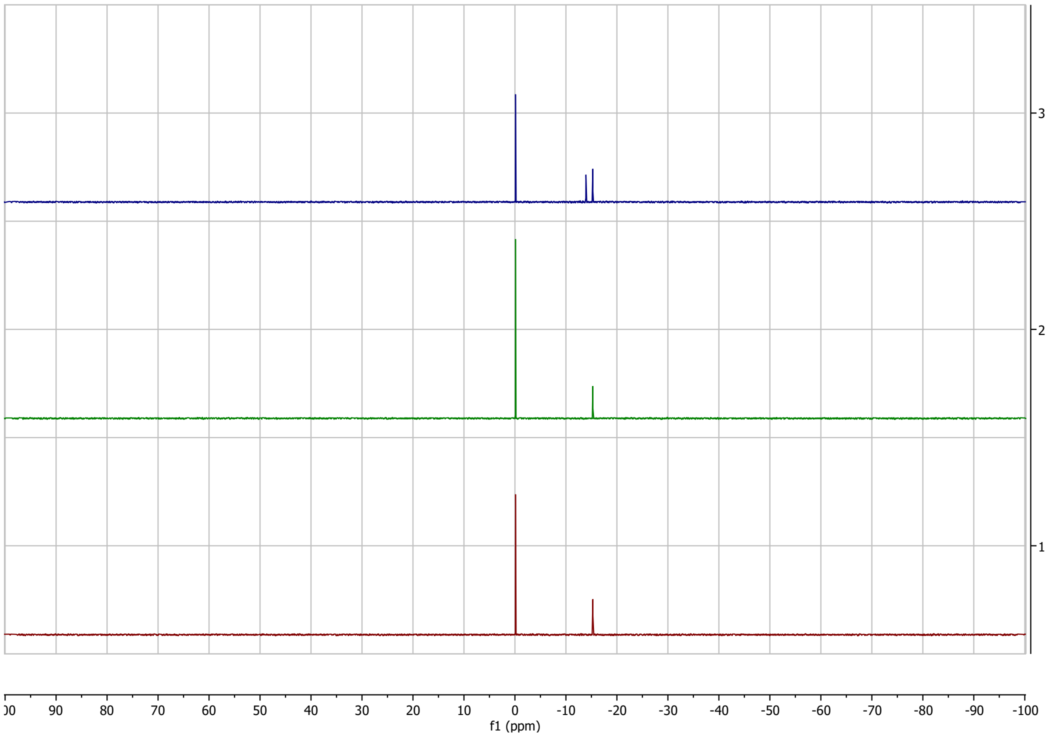

Figure S 33: ^{31}P-NMR of Wells-Dawson structure synthesized (Top = 25 °C, middle = 50 °C and bottom = 30 °C).

Keggin tungstate

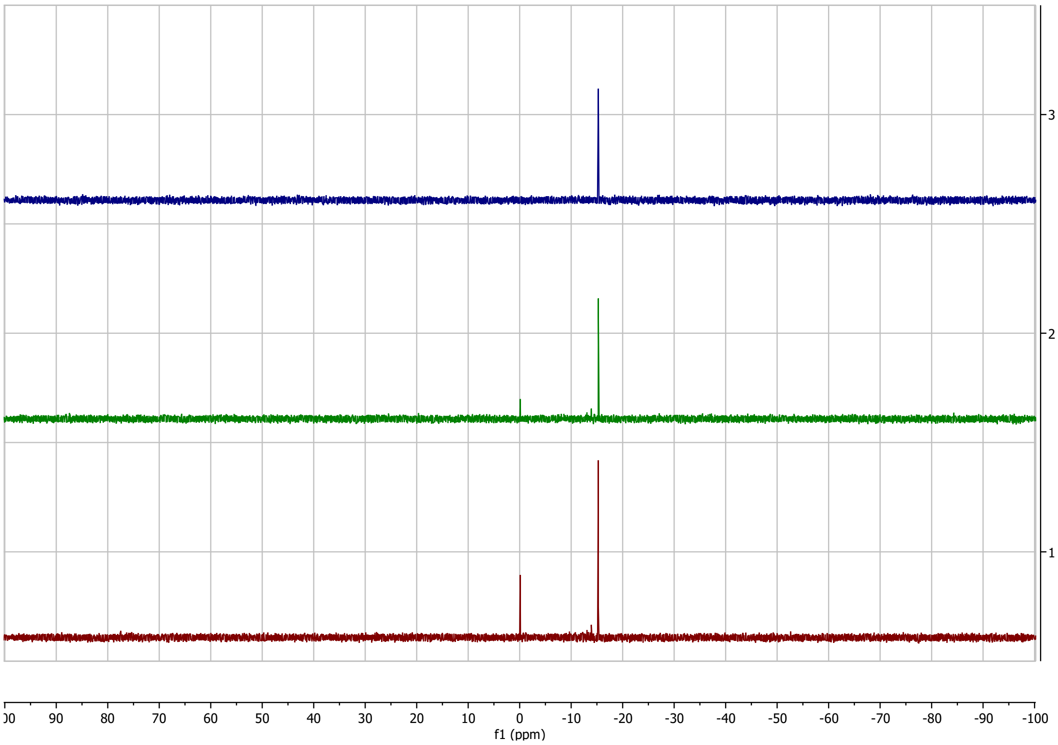

Figure S 34: ^{31}P-NMR spectra of Keggin molybdate synthesis (Top = 0 °C, middle = 10 °C and bottom = 25 °C).

Keggin molybdate

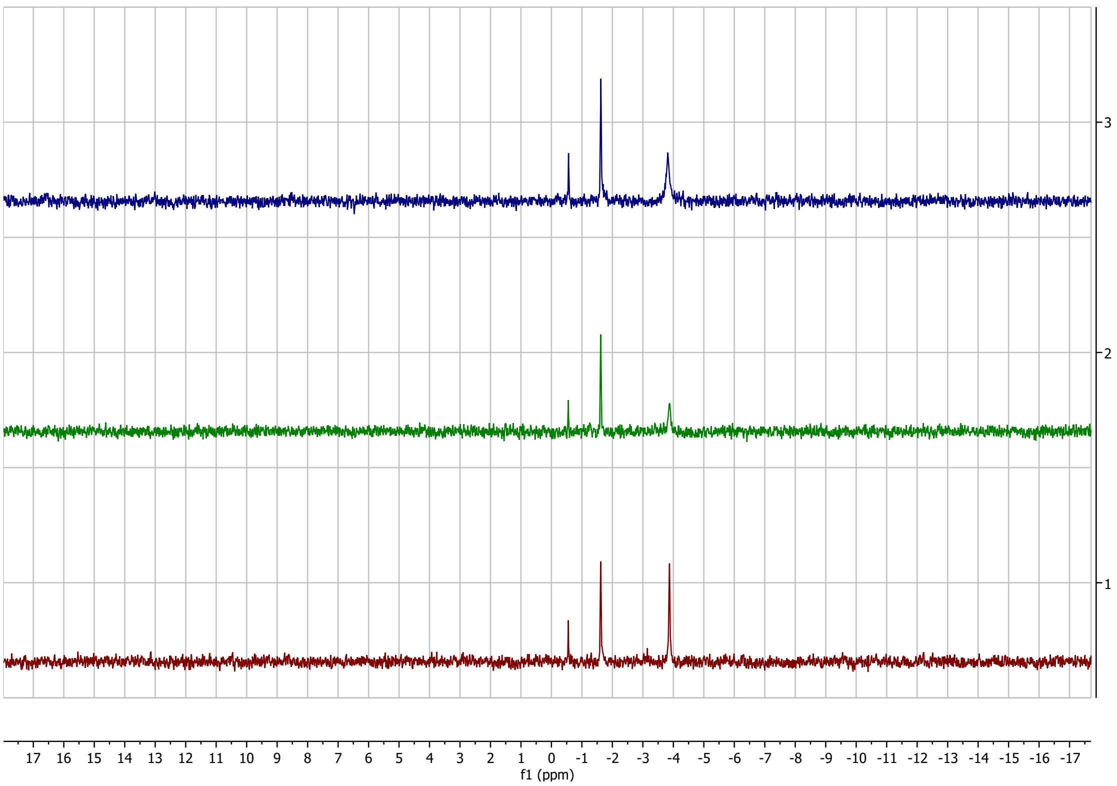

Figure S 35: ^{31}P-NMR spectra of Keggin molybdate synthesis (Top = 0 °C, middle = 10 °C and bottom = 25 °C).

$[PV_2Mo_{10}O_{40}]^{5-}$ via self-assembly

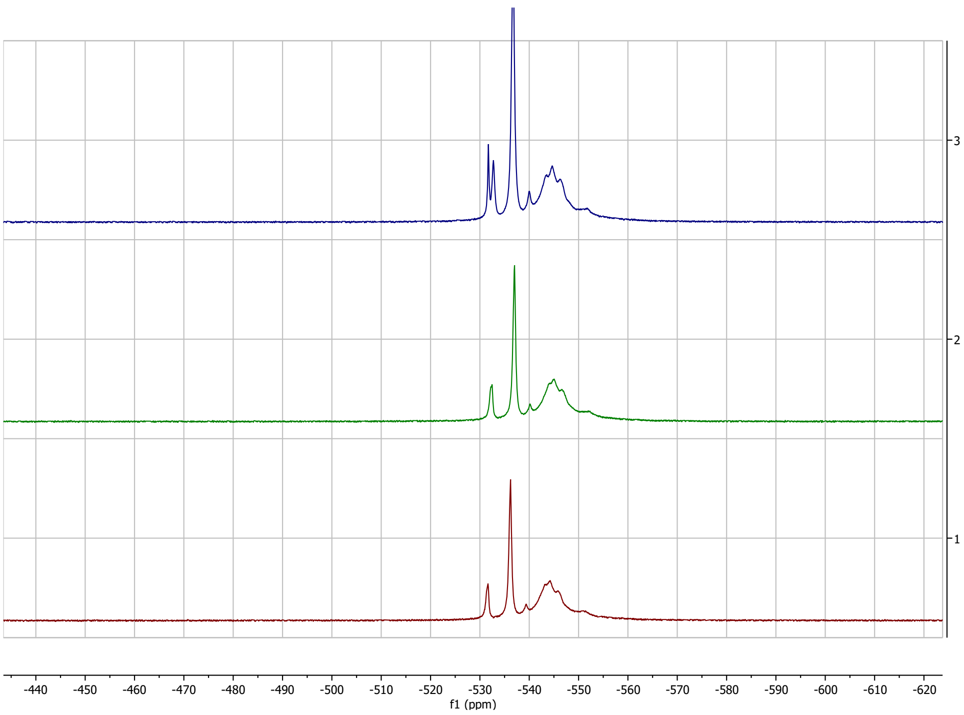

Figure S 36: ^{51}V-NMR spectra of vanadium substituted Keggin molybdate synthesis by self-assembly (Top = 25 °C, middle = 50 °C and bottom = 100 °C.

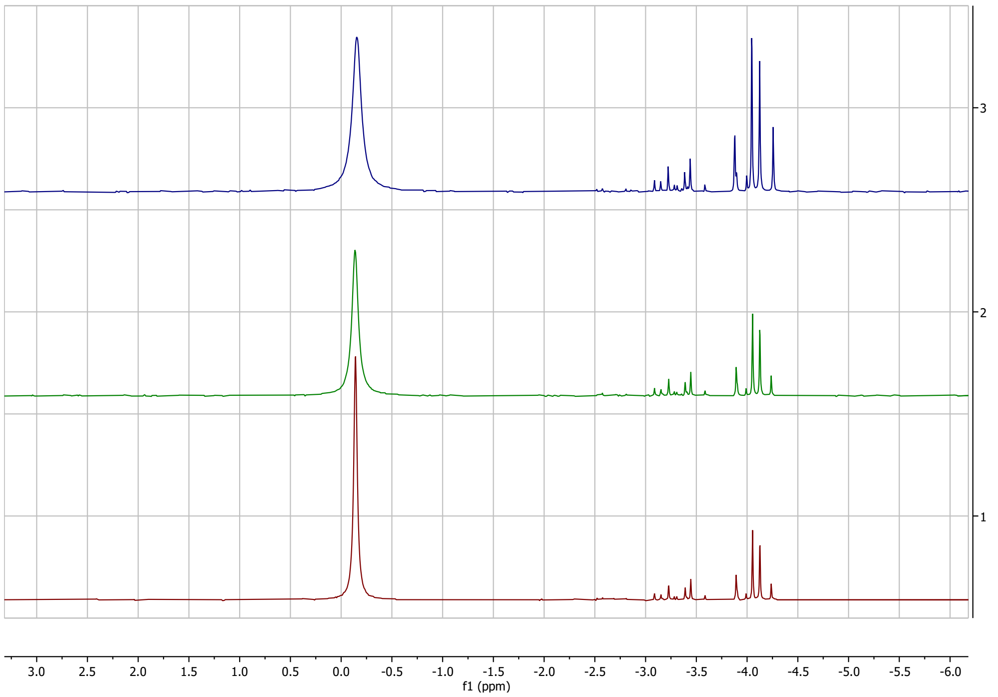

Figure S 37: ^{31}P-NMR spectra of vanadium substituted Keggin molybdate synthesis by self-assembly (Top = 25 °C, middle = 50 °C and bottom = 100 °C.

$[PV_2Mo_{10}O_{40}]^{5-}$ via lacunary route

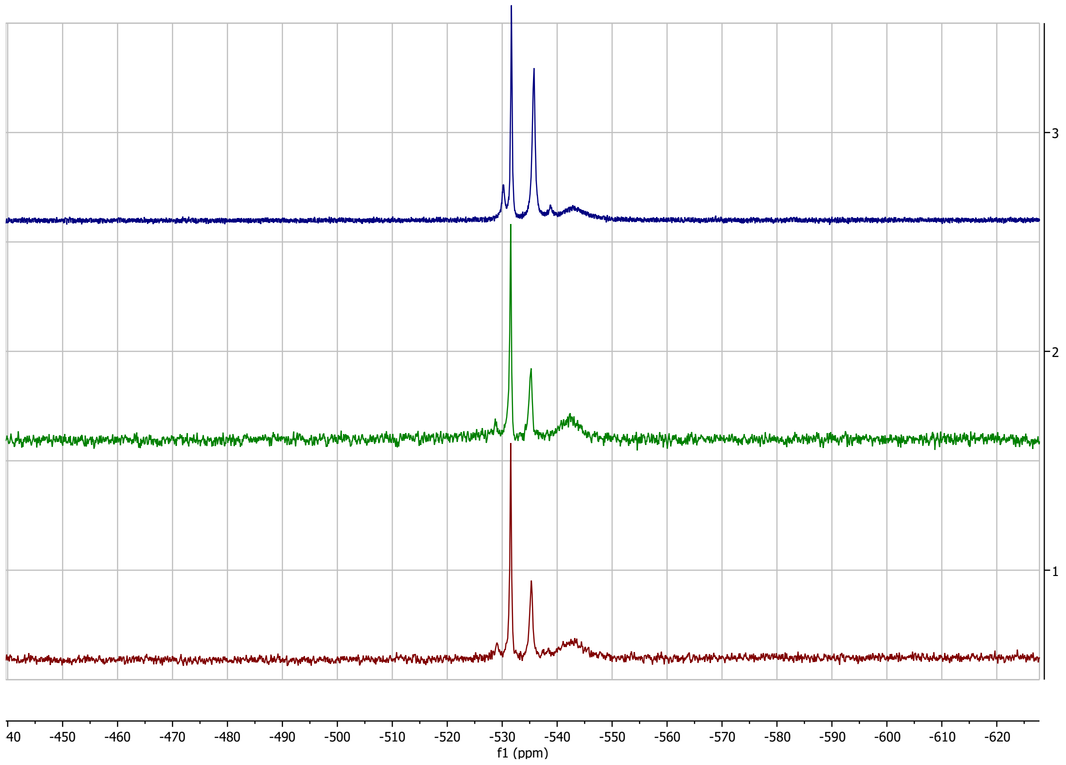

Figure S 38: ^{51}V-NMR spectra of vanadium substituted Keggin molybdate synthesis by Lacunary approach (Top = 25 °C, middle = 10 °C and bottom = -5 °C.

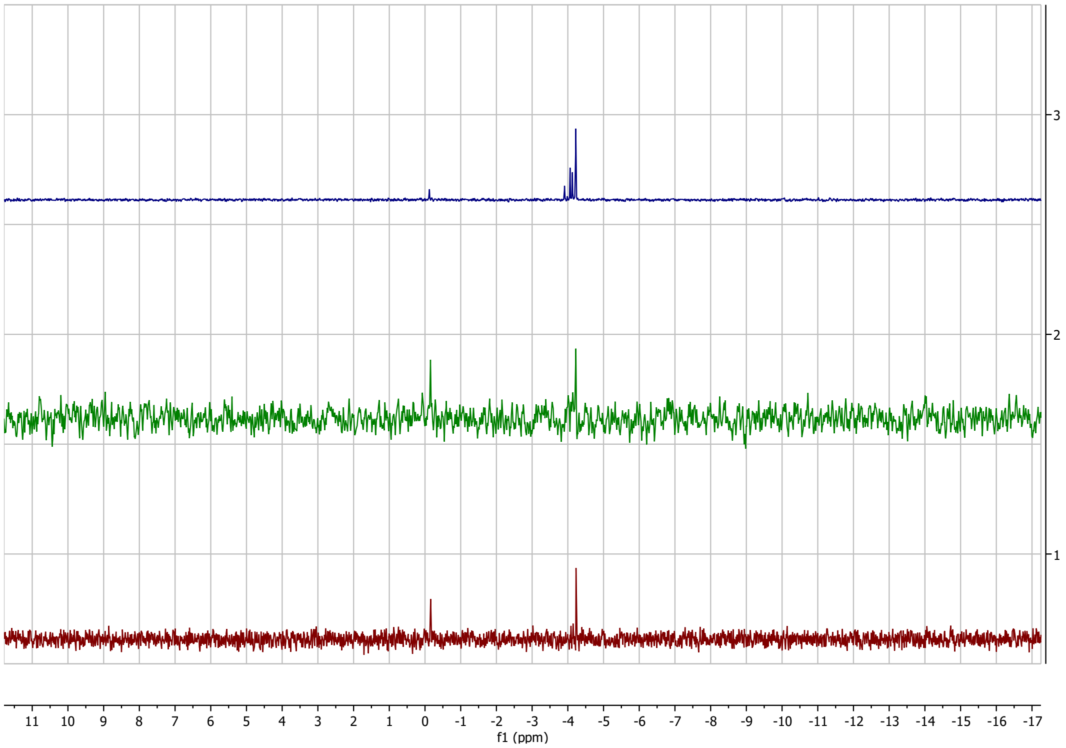

Figure S 39: ^{31}P-NMR spectra of vanadium substituted Keggin molybdate synthesis by Lacunary approach (Top = 25 °C, middle = 10 °C and bottom = -5 °C.

Elemental Analysis

Anderson-Evans

Table S 14: Elemental composition of synthesized compounds (ICP-OES)

Reaction conditions / measured element	Na	W	Te
T = 25 °C	10	6	1
T = 10 °C	13.9	6	1
T = -5 °C	10	6	1

Wells-Dawson

Table S 15: Elemental composition of synthesized compounds (ICP-OES)

Reaction conditions / measured element	Na	P	W
T = 25 °C (optimum pH)	41	2.2	18
T = 80 °C	30.6	2.8	18
T = 50 °C	26.8	2.8	18
T = 30 °C	41	3	18

Keggin tungstate

Table S 16: Elemental composition of synthesized compounds (ICP-OES)

Reaction conditions / measured element	Na / Na after membrane separation	P/ P after membrane separation	W / W after membrane separation
T = 25 °C	19 / -	1 / 1	12 / 12
T = 10 °C	14.6 / 5	1 / 1	12 / 12
T = 0 °C	30.7 / 6.5	1 / 1	12 / 12

Keggin molybdate

Table S 17: Elemental composition of synthesized compounds (ICP-OES)

Reaction conditions / measured element	Na / Na after membrane separation	P / P after membrane separation	Mo / Mo after membrane separation
T = 25 °C	24 / -	1.1 / 1	12 / 12
T = 10 °C	22.5 / 7	1.1 / 1	12 / 12
T = 0 °C	18 / 6	1.1 / 1	12 / 12

$[PV_2Mo_{10}O_{40}]^{5-}$ via self-assembly

Table S 18: Elemental composition of synthesized compounds (ICP-OES)

Reaction conditions / measured element	P	V	Mo
T = 100 °C	4.4	3.9	10
T = 50 °C	4.5	4	10
T = 25 °C	4.5	3.9	10

$[PV_2Mo_{10}O_{40}]^{5-}$ via lacunary route

Table S 19: Elemental composition of synthesized compounds (ICP-OES)

Reaction conditions / measured element	Na / Na after membrane separation	P / P after membrane separation	Mo / Mo after membrane separation	V / V after membrane separation
T = 25 °C	19.7 / 4	1.1 / 1	10 /10	2 / 2
T = 10 °C	17.8 / 5	1.1 / 1	10 / 10	1.9 / 2
T = - 5 °C	13.5 / 5	1 / 1	10 / 10	1.8 / 2

Additionally, iron was found after membrane separation experiments in the investigated samples. This was related to slight corrosion of the stainless steel membrane separation setup.

Spectroscopic data of $H_5PV_2Mo_{10}O_{40}$ (HPA-2) synthesized by optimized method

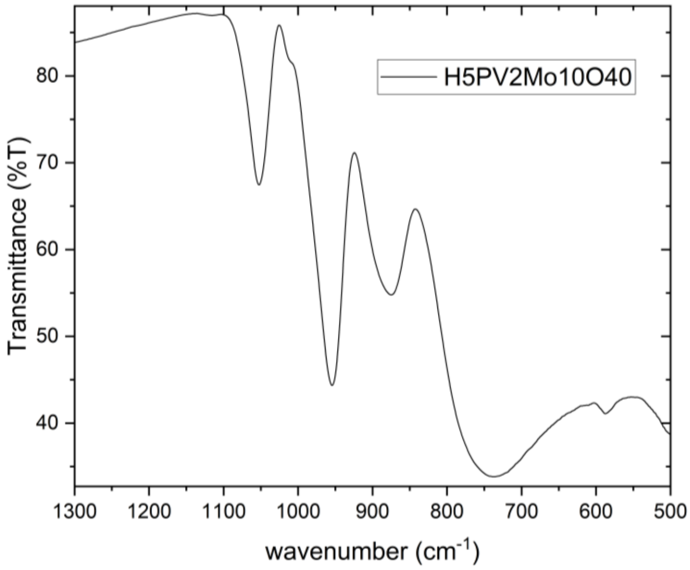

Figure S 40: IR spectrum (solid state) of synthesized HPA-2 by optimized synthesis procedure.

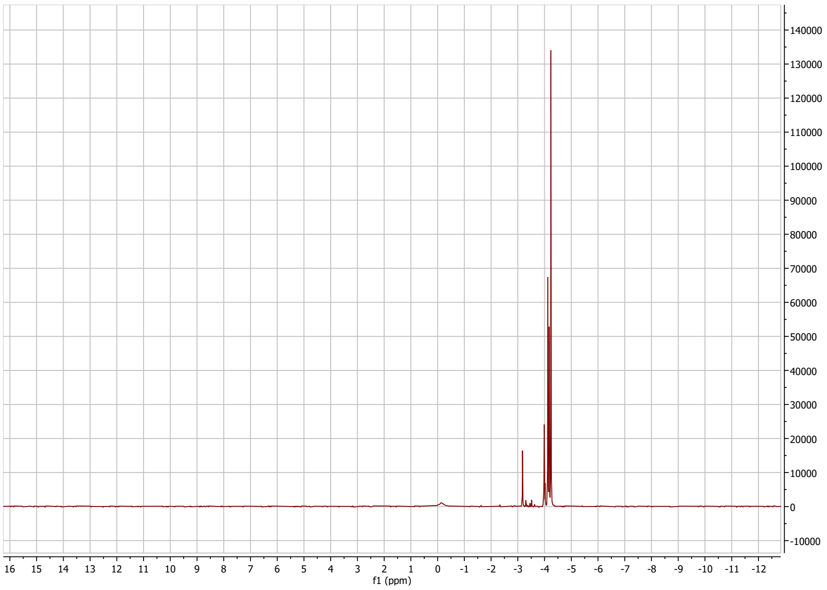

Figure S 41: ^{31}P-NMR spectrum of synthesized HPA-2 by optimized synthesis procedure.

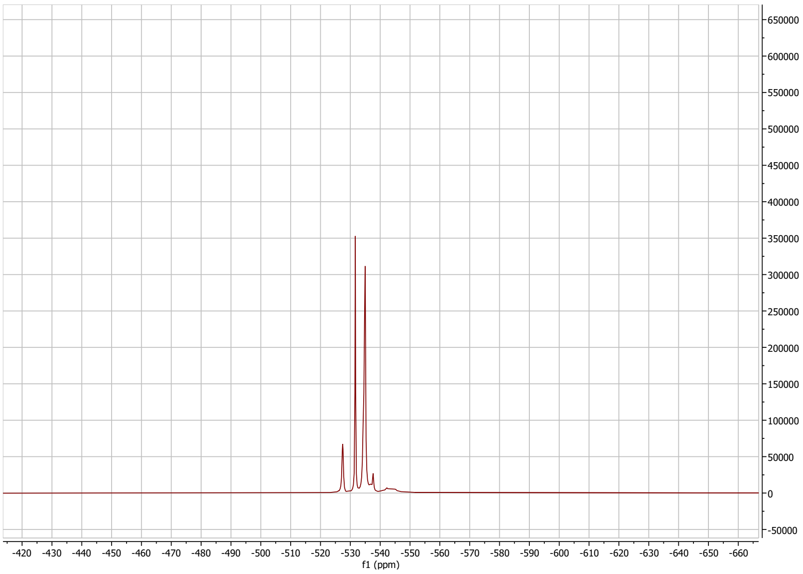

Figure S 42: ^{51}V-NMR spectrum of synthesized HPA-2 by optimized synthesis procedure.

Chemicals

- Sodium tungstate dihydrate: VWR chemicals (≥ 99 % for analysis)
- Sodium molybdate dihydrate: Carl Roth with a purity of 99.5 %
- Sodium vanadium oxide: Alfa Aesar with a purity of 96 %
- Molybdenum trioxide from Alfa Aesar, Haverhill, MA, USA
- Divanadium pentoxide: 99 %, Alfa Aesar
- Telluric acid: Sigma aldrich with a purity of 97 %
- Hydrogen peroxide: 30 % in water, VWR chemicals
- Phosphoric acid: was purchased as a 85 % solution in water from Grüssing
- 37 % Hydrochloric acid solution in water: 37 % VWR chemicals

Procedures, times and pH values for Anderson-Evans synthesis

	T = 25 °C		T = 10 °C			T = 0 °C
**Start Time**	**Action / Note / Sample**	**Start Time**	**Action / Note / Sample**		**Start Time**	**Action / Note / Sample**
00:00:00	Start of experiment on 24.01.2024 at 16:43:24 with thermostat off and stirrer off, Water=90 ml Details: Initially the operation started as 'First Fill and Safety Limits First fill of 90 ml of Water'	00:00:00	Start of experiment on 24.01.2024 at 17:15:57 with thermostat off and stirrer off, Water=90 ml Details: Initially the operation started as 'First Fill and Safety Limits First fill of 90 ml of Water'		00:00:00	Start of experiment on 25.01.2024 at 10:23:42 with thermostat off and stirrer off, Water=90 ml Details: Initially the operation started as 'First Fill and Safety Limits First fill of 90 ml of Water'
00:00:05	Ramp stirrer speed to 300 rpm over 10 sec	00:00:05	Ramp stirrer speed to 300 rpm over 10 sec		00:00:06	Ramp stirrer speed to 300 rpm over 10 sec
00:00:05	Heat Tr to 25 °C as fast as possible	00:00:05	Cool Tr to 10 °C as fast as possible		00:00:06	Cool Tr to -5 °C as fast as possible
00:00:18	Wait 2 min	00:00:17	Wait 2 min		00:00:17	Wait 2 min
00:00:27	Ramp stirrer speed to 0 rpm	00:02:17	Add 5 g of Na2 WO4 .2 H2O at once		00:02:17	Add 5 g of Na2 WO4 .2 H2O at once
00:00:46	Ramp stirrer speed to 300 rpm	00:09:41	pH 4.32		00:08:18	pH 4.79
00:02:18	Add 5 g of Na2 WO4 .2 H2O at once	00:09:47	#addition of wolframate		00:08:26	first problem with air bubble
00:04:22	pH 4.99	00:09:55	pH 9.81		00:08:34	but now nt anymore
00:04:41	start addition of Wolframat	00:10:06	Wait 2 min		00:10:08	addition of wolframate
00:05:22	pH 9.74	00:12:06	Add 0.58 g of TeO6H6 at once		00:10:16	Wait 2 min
00:06:03	pH 9.71	00:13:29	pH 9.84		00:10:35	pH 10.12
00:08:34	pH 9.68	00:14:28	Wait 2 min		00:11:21	pH 10.22
00:10:14	pH 9.66	00:14:31	addition of telluric acid		00:12:16	Add 0.58 g of TeO6H6 at once
00:10:50	addition of telluric acid	00:14:33	finished		00:13:39	pH 10.25
00:11:21	addition ended	00:14:37	pH 9.05		00:17:18	addition of telluric acid7
00:11:26	pH 8.74	00:16:02	pH 9.17		00:17:23	pH 9.51
00:11:30	Wait 2 min	00:16:28	Add 1.77 g of HCl at once		00:17:38	Wait 2 min
00:13:30	Add 0.58 g of TeO6H6 at once	00:16:36	pH 9.20		00:17:58	pH 9.29
00:15:07	pH 8.85	00:17:29	addition of HCl to adjust pH to 5		00:19:00	Ramp stirrer speed to 500 rpm
00:15:37	addition of HCl to adjust pH to 5	00:17:46	pH 7		00:19:38	Add 1.77 g of HCl at once
00:15:59	pH 2.86	00:17:49	pH 5		00:19:52	Heat Tr to 0 °C as fast as possible
00:16:07	Wait 2 min	00:17:54	pH 4.14		00:20:10	formation of ice in reactor
00:16:16	pH 2.97	00:18:02	pH 4.08		00:20:16	pH 9.43
00:16:36	pH 3.04	00:21:40	pH 4.15		00:20:26	freezing after addition of telluric acid
00:17:16	pH 3.12	00:23:18	pH 4.18		00:21:57	Heat Tr to 5 °C as fast as possible
00:18:07	Add 1.77 g of HCl at once	00:26:01	pH 4.21		00:24:26	Ramp stirrer speed to 300 rpm
00:19:37	pH 3.24	00:26:14	End of experiment on 24.01.2024 at 17:42:11 with thermostat off and stirrer off Details: Initially the operation started as 'End Experiment thermostat off and stirrer off'		00:28:34	Cool Tr to 2 °C as fast as possible
00:24:34	pH 3.37				00:38:32	pH 9.42

00:27:20	End of experiment on 24.01.2024 at 17:10:44 with thermostat off and R set to 100 rpm<br>Details:<br>Initially the operation started as 'End Experiment thermostat off and R set to 100 rpm'				00:38:40	Cool Tr to 0 °C as fast as possible
					00:41:59	pH 9.47
					00:42:02	addition of HCl
					00:42:42	addition finished
					00:42:48	turbidity increased directly
					00:42:52	pH 7.23
					00:43:07	reaction solution clear again
					00:43:37	pH 6.92
					00:44:14	pH 6.63
					00:47:01	anderson evans structure has formed
					00:52:37	End of experiment on 25.01.2024 at 11:16:19 with Tj set to 25 °C and R set to 100 rpm<br>Details:<br>Initially the operation started as 'End Experiment Tj set to 25 °C and R set to 100 rpm'

Procedures, times and pH values for Wells-Dawson synthesis

	T = 80 °C		T = 50 °C		T = 25 °C
**Start Time**	**Action / Note / Sample**	**Start Time**	**Action / Note / Sample**	**Start Time**	**Action / Note / Sample**
00:00:00	Start of experiment on 25.01.2024 at 14:19:32 with thermostat off and stirrer off, Water=90 ml Details: Initially the operation started as 'First Fill and Safety Limits First fill of 90 ml of Water'	00:00:00	Start of experiment on 25.01.2024 at 15:19:28 with thermostat off and stirrer off, Water=90 ml Details: Initially the operation started as 'First Fill and Safety Limits First fill of 90 ml of Water'	00:00:00	Start of experiment on 25.01.2024 at 16:18:59 with thermostat off and stirrer off, Water=90 ml Details: Initially the operation started as 'First Fill and Safety Limits First fill of 90 ml of Water'
00:00:05	Ramp stirrer speed to 300 rpm over 10 sec	00:00:05	Ramp stirrer speed to 300 rpm over 10 sec	00:00:05	Ramp stirrer speed to 300 rpm over 10 sec
00:00:05	Heat Tr to 80 °C as fast as possible	00:00:05	Heat Tr to 50 °C as fast as possible Details: Initially the operation started as 'Heat Tr to 80 °C as fast as possible' After 00:04:16, user changed the operation to 'Heat Tr to 50 °C as fast as possible'	00:00:05	Heat Tr to 30 °C as fast as possible
00:00:18	Add 5 g of Na2 WO4 .2 H2O at once	00:00:17	Add 5 g of Na2 WO4 .2 H2O at once	00:00:17	Add 5 g of Na2 WO4 .2 H2O at once
00:01:05	Ramp stirrer speed to 0 rpm	00:04:00	pH 4.95#	00:03:11	pH 4.07
00:01:20	Ramp stirrer speed to 100 rpm	00:08:37	pH 4.95	00:05:22	pH 4.20
00:01:22	Ramp stirrer speed to 100 rpm	00:11:06	pH 5.11	00:06:02	addition of wolframat
00:01:27	Ramp stirrer speed to 300 rpm	00:12:35	addition of wolframat	00:06:47	pH 7
00:06:40	water at 70 °C	00:13:22	addition finished	00:06:53	pH 9.85
00:06:57	pH 4.72	00:13:25	pH 9.61	00:06:59	pH 9.87
00:11:23	addition of wolframat	00:13:39	Wait 2 min	00:08:13	Wait 2 min
00:12:16	addition finished	00:15:06	pH 9.58	00:09:33	pH 9.84
00:12:19	pH 9.07	00:15:39	Add 4.2 ml of HCl at once	00:10:13	Add 4.2 ml of HCl at once
00:12:33	Wait 2 min	00:18:14	9.54	00:10:41	addition of HCl 1st time
00:13:25	pH 9.04	00:22:01	pH 9.50	00:10:42	end
00:14:33	Add 4.2 ml of HCl at once	00:22:09	addition of HCl	00:10:46	pH 7.72
00:15:15	pH 9.00	00:22:18	finished	00:10:52	pH 7.49
00:17:10	addition of HCl first time	00:22:23	pH 7.1	00:10:57	pH 7.47
00:17:37	Wait 2 min	00:22:31	Wait 2 min	00:11:02	Wait 2 min
00:17:48	pH 6.60	00:22:56	pH 7.03	00:11:52	pH 7.45
00:18:54	pH 6.55	00:24:31	Add 0.85 ml of phosphoric acid at once	00:13:02	Add 0.85 ml of phosphoric acid at once
00:19:37	Add 0.85 ml of phosphoric acid at once	00:27:31	pH 7.01	00:15:23	pH 7.44
00:21:26	pH 6.55	00:33:24	addition of H3PO4	00:19:23	addition of H3PO4
00:23:37	pH 6.56	00:33:37	finished	00:19:26	finito
00:24:06	addition of phosphoric acid	00:33:50	pH 7.02+pH 7.08	00:19:36	pH 7.35
00:24:10	Wait 2 min	00:33:55	pH 7.10	00:20:51	pH 7.49
00:24:14	addition finished	00:34:30	pH 7.14	00:20:58	Wait 2 min
00:24:24	pH 6.70	00:37:21	pH 7.21	00:21:47	pH 7.51

00:24:41	pH 6.75	00:42:21	Wait 2 min	00:22:50	pH 7.52
00:25:04	pH 6.78	00:42:47	pH 7.25	00:22:58	Add 20.6 g of HCl at once<br>Comment: to pH 1.15
00:26:10	Add 20.792 g of HCl at once<br>Comment: to pH 1.15<br>Details:<br>After 00:06:25, user changed the operation to 'Add 20.792 g of HCl at once'	00:44:21	Add 20.6 g of HCl at once<br>Comment: to pH 1.15	00:25:32	pH 7.55
00:27:06	pH 6.83	00:44:26	additionf of HCl 2nd	00:27:46	pH 7.56
00:29:10	pH 6.83	00:44:34	pH 1.30	00:29:25	addition of HCl
00:32:10	addition of 20 g HCl	00:44:39	pH 1.22	00:29:27	end
00:32:12	ended	00:49:26	pH 1.15	00:29:31	pH 1.9
00:32:19	pH1.25	00:54:09	pH 1.14	00:29:35	pH 1.37
00:32:22	pH 1.22	00:55:57	End of experiment on 25.01.2024 at 16:15:25 with Tj set to 25 °C and R set to 100 rpm<br>Details:<br>Initially the operation started as 'End Experiment Tj set to 25 °C and R set to 100 rpm'	00:29:40	pH 1.29
00:32:32	pH 1.17			00:29:45	pH 1.26
00:33:56	pH 1.12			00:30:08	pH 1.20
00:38:37	pH 1.07			00:30:40	pH 1.19
00:41:09	pH 1.06			00:33:14	pH 1.17
00:55:31	pH 1.00			00:34:43	pH 1.16
00:55:46	End of experiment on 25.01.2024 at 15:15:19 with Tj set to 25 °C and R set to 100 rpm<br>Details:<br>Initially the operation started as 'End Experiment Tj set to 25 °C and R set to 100 rpm'			00:37:23	pH 1.14
				00:44:58	pH 1.10
				00:45:19	End of experiment on 25.01.2024 at 17:04:18 with thermostat off and R set to 100 rpm<br>Details:<br>Initially the operation started as 'End Experiment thermostat off and R set to 100 rpm'

Procedures, times and pH values for Keggin tungstate synthesis

	T = 25 °C		T = 10 °C		T = 0 °C
**Start Time**	**Action / Note / Sample**	**Start Time**	**Action / Note / Sample**	**Start Time**	**Action / Note / Sample**
00:00:00	Start of experiment on 25.01.2024 at 17:16:15 with thermostat off and stirrer off, Water=90 ml Details: Initially the operation started as 'First Fill and Safety Limits First fill of 90 ml of Water'	00:00:00	Start of experiment on 26.01.2024 at 11:23:51 with thermostat off and stirrer off, Water=90 ml Details: Initially the operation started as 'First Fill and Safety Limits First fill of 90 ml of Water'	00:00:00	Start of experiment on 26.01.2024 at 12:10:58 with thermostat off and stirrer off, Water=90 ml Details: Initially the operation started as 'First Fill and Safety Limits First fill of 90 ml of Water'
00:00:06	Heat Tr to 25 °C as fast as possible	00:00:05	Cool Tr to 10 °C as fast as possible Details: Initially the operation started as 'Heat Tr to 25 °C as fast as possible' After 00:00:03, user changed the operation to 'Cool Tr to 10 °C as fast as possible'	00:00:05	Cool Tr to 0 °C as fast as possible
00:00:06	Ramp stirrer speed to 300 rpm over 10 sec	00:00:05	Ramp stirrer speed to 300 rpm over 10 sec	00:00:05	Ramp stirrer speed to 300 rpm over 10 sec
00:01:07	pH 4.17	00:01:20	Ramp stirrer speed to 0 rpm	00:02:23	Exposure time changed to 8.5 seconds
00:01:52	Add 5 g of Na2 WO4 .2 H2O at once	00:01:39	Ramp stirrer speed to 300 rpm	00:03:15	pH 4.58
00:02:19	Wait 2 min	00:02:23	Ramp stirrer speed to 700 rpm	00:07:34	Add 5 g of Na2 WO4 .2 H2O at once
00:02:25	pH (	00:02:31	Ramp stirrer speed to 300 rpm	00:08:10	addition of wolframate
00:02:25	pH (	00:05:05	Exposure time changed to 3.1 seconds	00:08:13	Wait 2 min
00:02:31	Ph):(/	00:05:06	Add 5 g of Na2 WO4 .2 H2O at once	00:08:22	pH 10.27
00:02:31	Ph):(/	00:10:33	pH 3.93	00:09:40	pH 10.30
00:02:41	pH 9.91	00:12:57	addition of wolframat pH 10.20	00:09:45	temp at 0 °C
00:02:41	pH 9.91	00:13:02	Wait 2 min	00:10:13	Add 1 g of phosphoric acid at once
00:03:23	addition of wolframat finished	00:15:02	Add 1 g of phosphoric acid at once	00:10:49	pH 10.32
00:03:23	addition of wolframat finished	00:17:39	pH 10.17	00:12:58	Wait 2 min
00:03:52	pH 9.92	00:17:40	pH 10.17	00:13:03	addition of H3PO4
00:03:53	pH 9.92	00:18:24	Wait 2 min	00:13:09	pH 8.62
00:04:03	addition of phosphoric acid	00:18:48	pH 8.41	00:13:12	pH 8.56
00:04:03	addition of phosphoric acid	00:18:48	pH 8.41	00:14:53	pH 8.49
00:04:09	pH 7.98	00:18:52	pH 8.30	00:14:58	Add 1.99 g of HCl at once
00:04:10	pH 7.98	00:18:52	pH 8.30	00:16:23	addition of first HCl
00:04:19	Add 1 g of phosphoric acid at once	00:19:42	pH 8.23	00:16:55	pH 7.7
00:04:22	Wait 2 min	00:19:42	pH 8.23	00:17:00	pH 7.3
00:05:17	pH 7.93	00:20:24	Add 1.99 g of HCl at once	00:17:03	pH 7.

00:05:17	pH 7.93	00:23:26	pH 8.24	00:17:07	pH 5.5
00:05:39	adjustement pH	00:23:26	pH 8.24	00:17:11	pH 4.36
00:05:39	adjustement pH	00:23:33	addition of HCl to pH 5	00:17:15	pH 4.26
00:05:47	adjust to pH 5	00:23:33	addition of HCl to pH 5	00:21:36	pH 4.94
00:05:48	adjust to pH 5	00:23:41	pH 7.5	00:21:46	Wait 2 min
00:05:57	pH 7.7	00:23:41	pH 7.5	00:23:47	Add 2.19 g of HCl at once
00:05:57	pH 7.7	00:23:44	pH 7.2	00:24:06	pH 5.18
00:05:57	pH 7.7	00:23:44	pH 7.2	00:24:15	addition of 2nd HCl
00:06:02	pH 7.6	00:23:48	pH 7	00:24:23	pH 4.9
00:06:02	pH 7.6	00:23:48	pH 7	00:24:25	pH 3.4
00:06:22	Add 1 g of HCl at once	00:23:51	pH 6.4	00:24:28	pH 1.8
00:10:10	00:00:00	00:23:51	pH 6.4	00:24:30	pH 1.4
00:10:10	00:00:00	00:23:55	pH 6.4	00:24:33	pH 1.28
00:11:39	5,85	00:23:55	pH 6.4	00:24:36	pH 1.23
00:11:39	5,85	00:23:59	pH 6.3	00:25:43	pH 1.09
00:11:39	5,85	00:24:00	pH 6.3	00:29:25	pH 0.98
00:15:13	pH 6.08	00:24:06	Wait 2 min	00:30:53	pH 0.96
00:15:13	pH 6.08	00:26:06	Add 2.19 g of HCl at once	00:38:45	End of experiment on 26.01.2024 at 12:49:43 with Tj set to 25 °C and R set to 100 rpm<br>Details:<br>Initially the operation started as 'End Experiment Tj set to 25 °C and R set to 100 rpm'
00:15:28	structure intermediate has formed	00:30:31	pH 6.63		
00:15:28	structure intermediate has formed	00:30:32	pH 6.63		
00:15:43	Wait 2 min	00:31:48	addition of HCl to pH 1		
00:15:53	pH 5.04	00:31:48	addition of HCl to pH 1		
00:15:53	pH 5.04	00:31:51	pH 3		
00:16:55	pH 5.38	00:31:51	pH 3		
00:16:55	pH 5.38	00:31:54	pH 2.06		
00:17:05	adjustement to pH 1	00:31:54	pH 2.06		
00:17:05	adjustement to pH 1	00:31:57	pH 1.47		
00:17:43	Add 1 g of HCl at once	00:31:57	pH 1.47		
00:18:04	pH 2.64	00:32:00	pH 1.33		
00:18:04	pH 2.64	00:32:00	pH 1.33		
00:18:23	pH 1.85	00:32:03	pH 1.22		
00:18:23	pH 1.85	00:32:04	pH 1.22		
00:18:30	pH 1.70	00:32:13	pH 1.19		
00:18:30	pH 1.70	00:33:03	pH 1.13		
00:18:30	pH 1.70	00:33:03	pH 1.13		
00:18:58	pH 1.48	00:34:01	pH 1.08		
00:18:58	pH 1.48	00:34:01	pH 1.08		
00:19:35	pH 1.34	00:41:57	End of experiment on 26.01.2024 at 12:05:49 with thermostat off and R set to 100 rpm<br>Details:<br>Initially the operation started as 'End Experiment thermostat off and R set to 100 rpm'		

00:20:02	pH 1.22				
00:20:02	pH 1.22				
00:20:59	pH 1.10				
00:21:00	pH 1.10				
00:21:21	pH 1.08				
00:21:22	pH 1.08				
00:21:42	pH 1.06				
00:21:42	pH 1.06				
00:22:08	pH 1.04				
00:22:08	pH 1.04				
00:25:10	pH 0.98				
00:25:10	pH 0.98				
00:27:39	End of experiment on 25.01.2024 at 17:43:54 with thermostat off and R set to 100 rpm<br>Details:<br>Initially the operation started as 'End Experiment thermostat off and R set to 100 rpm'				

Procedures, times and pH values for Keggin molybdate synthesis

	T = 30 °C		T = 10 °C		T = 0 °C
**Start Time**	**Action / Note / Sample**	**Start Time**	**Action / Note / Sample**	**Start Time**	**Action / Note / Sample**
00:00:00	Start of experiment on 26.01.2024 at 10:34:27 with thermostat off and stirrer off, Water=90 ml Details: Initially the operation started as 'First Fill and Safety Limits First fill of 90 ml of Water'	00:00:00	Start of experiment on 26.01.2024 at 13:43:28 with Tj set to 25 C and stirrer off, Water=90 ml Details: Initially the operation started as 'First Fill and Safety Limits First fill of 90 ml of Water'	00:00:00	Start of experiment on 26.01.2024 at 14:24:55 with Tj set to 25 C and stirrer off, Water=90 ml Details: Initially the operation started as 'First Fill and Safety Limits First fill of 90 ml of Water'
00:00:06	Heat Tr to 25 °C as fast as possible	00:00:05	Cool Tr to 10 °C as fast as possible	00:00:05	Cool Tr to 0 °C as fast as possible
00:00:06	Ramp stirrer speed to 300 rpm over 10 sec	00:00:05	Ramp stirrer speed to 300 rpm over 10 sec	00:00:05	Ramp stirrer speed to 300 rpm over 10 sec
00:01:21	Exposure time changed to 8.7 seconds	00:03:31	Exposure time changed to 9.7 seconds	00:00:50	Ramp stirrer speed to 500 rpm
00:01:45	Add 3.66 g of natrium molybdate dihydrate at once	00:04:53	Add 3.66 g of natrium molybdate dihydrate at once	00:01:04	Ramp stirrer speed to 300 rpm
00:04:05	Heat Tr to 30 °C as fast as possible	00:06:46	Exposure time changed to 8.6 seconds	00:03:22	Exposure time changed to 8.2 seconds
00:07:44	addition of Mo	00:07:34	pH 4.53	00:08:12	pH 4.53
00:08:52	pH 9.37	00:08:29	addition of molybdate	00:08:24	Add 3.66 g of natrium molybdate dihydrate at once
00:10:18	Wait 2 min	00:08:50	pH 6.37	00:08:54	addition of molybdate
00:12:18	Add 1 g of phosphoric acid at once	00:09:07	pH 7.55	00:09:05	pH 7
00:12:32	addition of H3PO4	00:09:11	Wait 2 min	00:09:08	pH 8
00:12:36	Wait 2 min	00:09:28	pH 8.7	00:09:12	pH 9
00:12:42	pH 6.3	00:09:31	pH 9.14	00:09:16	pH 9.41
00:12:47	pH 6.28	00:10:10	pH 9.7	00:09:18	pH 9.52
00:14:37	Add 1 g of HCl at once	00:11:11	Add 0.1456 g of phosphoric acid at once	00:09:23	pH 9.62
00:17:39	addition of HCl to pH 5	00:12:00	pH 9.69	00:09:25	pH 9.66
00:17:43	Wait 2 min	00:13:29	addition of H3PO4	00:09:29	Wait 2 min
00:18:09	pH 6.17	00:13:30	finished	00:10:21	pH 9.68
00:19:43	Add 1 g of HCl at once	00:13:32	pH 7	00:11:29	Add 0.1456 g of phosphoric acid at once
00:19:59	pH 5.93	00:13:35	pH 6.92	00:12:25	pH 9.69
00:22:30	pH 5.30	00:13:38	pH 6.67	00:12:55	addition of H3PO4
00:23:42	pH 5.13	00:13:41	pH 6.63	00:12:58	pH 9.5
00:24:30	pH 5.04	00:13:45	pH 6.61	00:13:01	p 8
00:26:01	pH 5.05	00:15:38	pH 6.68	00:13:03	pH 7
00:28:27	pH 4.8	00:19:44	pH 6.67	00:13:07	pH 6.88
00:28:34	pH 4.29	00:19:47	pH 5	00:13:13	pH 6.83
00:28:52	pH 4	00:19:52	pH 5.44	00:13:20	Wait 2 min
00:29:19	pH 2.20	00:19:59	pH 5.13	00:14:01	pH 6.81
00:29:23	pH 2.10	00:20:03	pH 5.07	00:15:20	Add 2.1287 g of HCl at once
00:30:05	pH 1.67	00:20:29	Wait 2 min	00:15:37	pH 6.85

00:30:10	pH 1.55	00:20:40	addition of 1st HCl	00:16:18	addition of 1st HCl
00:30:38	pH 1.47	00:22:29	Add 2.1287 g of HCl at once	00:16:26	pH 6.4
00:30:47	pH 1.46	00:22:34	Wait 2 min	00:16:28	pH 6.0
00:32:52	pH 1.16	00:23:04	pH 5.14	00:16:32	pH 5.7
00:38:16	solution turned yellow	00:24:34	Add 2.113 g of HCl at once	00:16:35	pH 5.12
00:38:45	pH 0,98	00:26:20	addition of 2nd HCl	00:16:39	pH 5.00
00:40:12	End of experiment on 26.01.2024 at 11:14:40 with Tj set to Tsafe and R set to 100 rpm<br>Details:<br>Initially the operation started as 'End Experiment Tj set to Tsafe and R set to 100 rpm'	00:26:22	pH 4	00:16:45	pH 4.96
		00:26:25	pH 3	00:16:51	pH 4.95
		00:26:27	pH 1.6	00:16:55	Wait 2 min
		00:26:29	pH 1.3	00:18:55	Add 2.113 g of HCl at once
		00:26:34	pH 1.23	00:25:43	addition of 2nd HCl
		00:26:37	pH 1.20	00:25:45	finis
		00:27:13	solution turned yellow instantaneously	00:25:47	pH 3
		00:29:27	pH 1.05	00:25:48	pH 2.5
		00:35:27	pH 1.00	00:25:51	pH 1.6
		00:36:47	End of experiment on 26.01.2024 at 14:20:16 with Tj set to 25 °C and R set to 100 rpm<br>Details:<br>Initially the operation started as 'End Experiment Tj set to 25 °C and R set to 100 rpm'	00:25:53	pH 1.4
				00:25:55	pH 1.3
				00:26:01	pH 1.24
				00:26:05	pH 1.21
				00:32:20	pH 0.98
				00:34:43	End of experiment on 26.01.2024 at 14:59:38 with Tj set to 25 °C and R set to 100 rpm<br>Details:<br>Initially the operation started as 'End Experiment Tj set to 25 °C and R set to 100 rpm'

Procedures, times and pH values for synthesis of $[PV_2Mo_{10}O_{40}]^{5-}$ via lacunary route

	T = 25 °C		T = 10 °C		T = -5 °C
**Start Time**	**Action / Note / Sample**	**Start Time**	**Action / Note / Sample**	**Start Time**	**Action / Note / Sample**
00:00:00	Start of experiment on 24.01.2024 at 13:21:23 with Tj set to 25 C and stirrer off, Water=90 ml Details: Initially the operation started as 'First Fill and Safety Limits First fill of 90 ml of Water'	00:00:00	Start of experiment on 24.01.2024 at 14:18:18 with thermostat off and stirrer off, Water=90 ml Details: Initially the operation started as 'First Fill and Safety Limits First fill of 90 ml of Water'	00:00:00	Start of experiment on 24.01.2024 at 15:13:14 with thermostat off and stirrer off, Water=90 ml, Details: Initially the operation started as 'First Fill and Safety Limits First fill of 90 ml of Water'
00:00:06	Heat Tr to 25 °C as fast as possible	00:00:05	Cool Tr to 10 °C as fast as possible	00:00:05	Cool Tr to -5 °C as fast as possible, Details:Initially the operation started as 'Cool Tr to 0 °C as fast as possible', After 00:00:32, user changed the operation to 'Cool Tr to -5 °C as fast as possible'
00:00:06	Ramp stirrer speed to 300 rpm over 10 sec	00:00:05	Ramp stirrer speed to 300 rpm over 10 sec	00:00:05	Ramp stirrer speed to 300 rpm over 10 sec
00:01:10	Add 5 g of natrium molybdate dihydrate at once	00:04:42	Add 5 g of natrium molybdate dihydrate at once	00:01:16	pH 5.0
00:02:41	pH 5.28	00:06:00	pH 4.54	00:05:53	pH 4.69
00:03:32	temperature 25 °C reached	00:07:55	pH 4.47	00:06:10	addition of molybdate at 5 °C
00:04:01	Wait 5 min	00:08:24	addition of molybdate	00:06:34	pH 7.8
00:04:35	addition of molybdate	00:08:47	Wait 5 min	00:06:39	pH 8.2
00:04:48	with 0.5 mL h2o gespült	00:08:56	addition finished	00:06:44	pH 8.5
00:05:21	pH 8.50	00:09:01	pH 7.92	00:06:54	addition finished
00:07:45	pH 8.26	00:09:10	pH 8.40	00:06:58	spülen mit tropfen wasser
00:09:01	Add 1 ml of dinatrium hydrogenphosphate at once Details: After 00:02:24, user changed the operation to 'Add 1 ml of dinatrium hydrogenphosphate at once'	00:09:21	pH 8.60	00:07:29	pH 8.69
00:10:41	pH 8.12	00:09:30	spülen mit paar Tropfen H2O	00:09:42	pH 8.56
00:10:55	addition of phosphate	00:11:32	pH 8.50	00:09:51	Add 5 g of natrium molybdate dihydrate at once
00:11:26	Wait 5 min	00:12:36	pH 8.43	00:10:05	Wait 5 min

00:11:38	addition completed	00:13:47	Add 1 g of dinatrium hydrogenphosphate at once	00:13:09	pH 8.39
00:11:45	pH 9´8.92	00:15:29	pH 8.30	00:15:05	Add 1 g of dinatrium hydrogenphosphate at once
00:16:26	Add 1 g of Natrium vanadate at once	00:16:02	addition of phosphate	00:16:17	pH 8.24
00:17:33	pH 8.92	00:16:07	Wait 5 min	00:16:34	addition of phosphate
00:17:41	next step addition of HCl	00:16:17	phosphate addition finished	00:16:44	addition finished
00:18:16	addition of HCl finished	00:16:24	plus addition of some water	00:16:51	additional water
00:18:20	pH 1.01	00:16:49	pH 9.01	00:16:59	pH9.02
00:18:58	colour change to yellow	00:21:07	Add 1 g of HCl at once	00:17:05	Wait 5 min
00:20:24	pH 0.92	00:24:06	pH 9.02	00:19:18	pH 9.05
00:26:47	pH 0.87	00:24:28	addition of HCl	00:21:45	pH 9.02
00:26:53	addition of vanadate	00:24:34	addition finished	00:22:05	Add 1 g of HCl at once
00:27:29	addition finisehd	00:24:41	pH 1.22	00:24:56	pH 9.06
00:27:33	colour change to orange	00:24:49	pH 1.10	00:25:04	steady state before addition of HCl
00:27:52	pH 1.01	00:24:56	solution turned yellow directly	00:25:27	addition of HCl
00:28:09	PV2Mo9OX formation	00:25:01	pH 1.06	00:25:32	addition finisehd
00:28:51	Wait 5min	00:29:07	pH 0.93	00:25:38	pH 2.
00:30:20	pH=1,06	00:30:07	Add 1 g of Natrium vanadate at once	00:25:40	1.5
00:33:51	Add 5 g of natrium molybdate dihydrate at once<br>Comment: 1 Eq. zum füllen der letzten Lacunary Lücke	00:31:00	addition of vanadate	00:25:42	1.4
00:39:43	pH 1.06	00:31:07	addition finished	00:25:45	1.3
00:39:57	addition of additional molybdate to finish keggin structure	00:31:12	Wait 5min	00:25:50	pH 1.26
00:40:24	finish addition	00:31:26	addition finished	00:25:56	turning slowly yellow
00:40:48	pH 1.26	00:31:30	pH 1.04	00:25:59	pH 1.18
00:45:39	pH 1.26	00:34:16	solution orange	00:28:44	pH 0.98

00:50:10	pH 1.25	00:34:18	pH 1.04		00:31:10	Add 1 g of Natrium vanadate at once
00:50:17	no change visible anymore	00:36:12	Add 5 g of natrium molybdate dihydrate at once Comment: 1 Eq. zum füllen der letzten Lacunary Lücke		00:32:54	pH 0.9
00:50:36	End of experiment on 24.01.2024 at 14:11:59 with thermostat off and R set to 100 rpm Details: Initially the operation started as 'End Experiment thermostat off and R set to 100 rpm'	00:39:00	addition of additional molybdate solution to finish keggin structure		00:33:19	solution colourless
		00:39:24	addition finished		00:33:59	addition of vanadate
		00:39:30	pH 1.29		00:34:06	addition finished
		00:49:27	End of experiment on 24.01.2024 at 15:07:45 with thermostat off and R set to 100 rpm Details: Initially the operation started as 'End Experiment thermostat off and R set to 100 rpm'		00:34:11	solution orange again
					00:34:20	pH 1.04
					00:34:55	Wait 5min
					00:39:55	Add 5 g of natrium molybdate dihydrate at once
					00:42:23	addition of Molybdate to finish Keggin
					00:42:28	pH 0.99
					00:42:34	addition start
					00:42:38	addition finished
					00:44:43	pH 1.18
					00:48:03	pH 1.18
					00:52:27	End of experiment on 24.01.2024 at 16:05:42 with Tj set to 25 °C and R set to 100 rpm Details: Initially the operation started as 'End Experiment Tj set to 25 °C and R set to 100 rpm'
